# The Flavone Cirsiliol from *Salvia x jamensis* Binds the F_1_ Moiety of ATP Synthase, Modulating Free Radical Production

**DOI:** 10.3390/cells11193169

**Published:** 2022-10-09

**Authors:** Lavinia Carlini, Gabriele Tancreda, Valeria Iobbi, Federico Caicci, Silvia Bruno, Alfonso Esposito, Daniela Calzia, Stefano Benini, Angela Bisio, Lucia Manni, Anna Schito, Carlo Enrico Traverso, Silvia Ravera, Isabella Panfoli

**Affiliations:** 1 Faculty of Science and Technology, Free University of Bolzano, 39100 Bolzano, Italy; 2 Dipartimento di Farmacia (DIFAR), University of Genoa, 16132 Genova, Italy; 3 Department of Biology, Università di Padova, 35121 Padova, Italy; 4 Department of Experimental Medicine, University of Genoa, 16132 Genoa, Italy; 5 International Centre for Genetic Engineering and Biotechnology (ICGEB), 34149 Trieste, Italy; 6 Sezione di Microbiologia, Dipartimento di Scienze Chirurgiche e Diagnostiche Integrate (DISC), Università di Genova, 16145 Genova, Italy; 7 Clinica Oculistica (DINOGMI), University of Genoa, 16132 Genova, Italy

**Keywords:** F_1_F_o_-ATP synthase, cirsiliol, light, oxidative phosphorylation, quercetin, respiratory chain complexes, resveratrol, rod outer segment, transmission electron microscopy

## Abstract

Several studies have shown that mammalian retinal rod outer segments (OS) are peculiar structures devoid of mitochondria, characterized by ectopic expression of the molecular machinery for oxidative phosphorylation. Such ectopic aerobic metabolism would provide the chemical energy for the phototransduction taking place in the OS. Natural polyphenols include a large variety of molecules having pleiotropic effects, ranging from anti-inflammatory to antioxidant and others. Our goal in the present study was to investigate the potential of the flavonoid cirsiliol, a trihydroxy-6,7-dimethoxyflavone extracted from *Salvia x jamensis*, in modulating reactive oxygen species production by the ectopic oxidative phosphorylation taking place in the OS. Our molecular docking analysis identified cirsiliol binding sites inside the F1 moiety of the nanomotor F_1_F_o_-ATP synthase. The experimental approach was based on luminometry, spectrophotometry and cytofluorimetry to evaluate ATP synthesis, respiratory chain complex activity and H_2_O_2_ production, respectively. The results showed significant dose-dependent inhibition of ATP production by cirsiliol. Moreover, cirsiliol was effective in reducing the free radical production by the OS exposed to ambient light. We report a considerable protective effect of cirsiliol on the structural stability of rod OS, suggesting it may be considered a promising compound against oxidative stress.

## 1. Introduction

As already widely established, polyphenols include a large variety of molecules exerting pleiotropic effects on different cellular and systemic targets of the human body [1,2]. Polyphenols, characterized by multiple phenolic rings, can be found mostly in plant-based foods and are divided into sub-categories, among which are phenolic acids, lignans, stilbenes, flavonoids and others. Important to mention is the role of these bioactive components in human health, playing a crucial role in prevention and modulation of several pathological pathways and onset of diseases [1,3,4].

A number of studies mostly conducted in vitro but also in vivo have highlighted the anti-inflammatory and antioxidant effects of polyphenols and their role in the prevention of cardiovascular and neurodegenerative diseases and cancer, as well as modulation of the cell aging process [5,6,7]. Furthermore, polyphenols display positive effects on the eye [6]. An important property of these molecules is their action in preventing cardiovascular and neurodegenerative diseases by lowering oxidative stress [4]. It was shown that polyphenols bind the F_1_ moiety of the F_1_F_o_-ATP synthase (ATP synthase), with high binding energy [8].

In the present study, we have focused on the mammalian retinal rod outer segments (OS), which were shown to express ectopic functional mitochondrial electron transport chains (ETCs), ATP synthase and tricarboxylic acid cycle enzymes and conduct oxidative phosphorylation (OxPhos) [9,10,11,12,13]. The rod OS is an organelle devoid of mitochondria and specialized in phototransduction, a process requiring a continual chemical energy supply [14,15], likely supplied by an ectopic OxPhos [11]. The ATP synthase ectopically expressed in the OS is inhibited by polyphenols [16], which modulate the oxidative stress by-products of the OxPhos [17]. In fact, upon light-exposure, the purified rod OS in vitro produce free radicals in a manner proportional to the light intensity [17]. Besides the stilbene resveratrol, two terpenoids extracted from *Salvia tingitana* [18] (manool and sclareol) also exhibited inhibitory effects on the ATP synthase and free radical production in the OS [19,20].

*Salvia* is the largest genus of the Lamiaceae family, consisting of about 980 species [21,22,23,24]. Further, 6-hydroxyflavones are the flavonoids that characterize *Salvia* spp.

Cirsiliol (3′,4′,5-trihydroxy-6,7-dimethoxyflavone) is a polyphenol belonging to the cited subcategory of flavones [25], first isolated from *Achillea fragrantissima* [26], whose presence in *S. officinalis* has also been reported. We isolated and characterized this metabolite from *Salvia x jamensis* [27] as a yellowish-green substance. We selected this flavone as our subject of analysis following our studies on how plant bioactive compounds interact and can affect OxPhos and modulate the resulting oxidative stress. It has been reported that cirsiliol has several bioactivities, such as antibacterial, antiproliferative and anti-inflammatory functions [28,29]. The anti-inflammatory action of cirsiliol would be exerted through its ability to modulate the signaling pathways concerning IL-6-induced activity by suppressing expression of the marker genes involved in the inflammatory response [28]. Furthermore, a wealth of recent scientific literature has established the properties of cirsiliol and its growth-inhibitory activities against various cancer cells [30]. Its potential as a bioactive phytochemical against malignant melanoma was recently explored [31]. Cirsiliol inhibited the activity and expression of matrix metalloproteinase-9 (MMP-9), as well as of the phosphatidylinositol-3-kinase (PI3K) signaling pathway [31]. Cirsiliol was able to directly bind the tyrosine kinase 2 (TYK2), targeting its signaling through STAT3, thus suppressing esophageal squamous cell carcinoma growth [32]. Worth mentioning as well are the sedative and hypnotic effects of cirsiliol, related to its role as a ligand for the central benzodiazepine receptors [33].

X-ray crystallography showed that resveratrol and related polyphenols bind to the F_1_ moiety of the nanomotor ATP synthase, inhibiting its rotary catalysis [8]. In this work, we carried out molecular docking analysis of the binding of cirsiliol to the ATP synthase and were able to identify the specific sites of interaction of the flavone responsible with the enzyme. Consistently, we showed that cirsiliol inhibited the ectopic rod OS ATP synthase and ETC in uncoupled conditions. Moreover, cirsiliol was able to prevent the structural and oxidative damage caused by free radical production consequent to light exposure in the OS.

## 2. Materials and Methods

### 2.1. General Experimental Procedures

All chemical compounds were of the highest chemical grade. In the course of the experimental work reported, all mandatory laboratory health and safety procedures were complied with.

#### 2.1.1. Plant Material

Aerial parts of *S. x jamensis* J. Compton were obtained from Centro Regionale di Sperimentazione ed Assistenza Agricola (Albenga, Italy). The species has been identified by Dr. Gemma Bramley, and a voucher specimen is deposited in Kew Herbarium (K).

#### 2.1.2. Extraction and Isolation of Cirsiliol

Extraction of cirsiliol from leaf surface constituents of *S. x jamensis* was performed as previously described [27]. Briefly, the exudate (18 g) was chromatographed in portions of 1.5 g on Sephadex LH-20 column (60 × 3 cm) using CHCl_3_/MeOH (7:3) as an eluent to give, in order of elution, four fraction groups. Fraction group IV (0.2 g) (from 295 mL to 365 mL), containing cirsiliol, was then purified with semi-preparative RP-HPLC, affording 20 mg of cirsiliol, identified by comparison of its spectroscopic data with those reported in literature [34].

Silica gel 60 (Merck230-400 mesh) was used for column chromatography; aluminum sheets of silica gel 60 F254 (Merck 0.2 mm thick) with CHCl_3_/MeOH/HCOOH (10:0.5:0.1) as an eluent were used for TLC, and the spots were detected by spraying 50% H_2_SO_4_, followed by heating. Semi-preparative HPLC was carried out using a Waters W600 pump (Waters Corporation, Milford, MA, USA) equipped with a Rheodyne Delta 600 Injector (with a 100 μL loop) and a Waters 2414 Refractive Index detector. Semi-preparative reversed-phase chromatography was performed at room temperature on a chemically bonded stationary phase 10 μm μ Bondapack C18 column (7.8 × 300 mm ID) (Waters). The elution mixture (helium-degassed) was composed of CH_3_OH/H_2_O 40:60. The flow rate was 2.0 mL/min. NMR experiments were performed on a Bruker DRX-600 spectrometer (Bruker BioSpin GmBH, Rheinstetten, Germany) equipped with a Bruker 5 mm TCI CryoProbe at 300 K and a Bruker DRX-400 spectrometer. All 2D NMR spectra were acquired in CDCl*3*, and standard pulse sequences and phase cycling were used for TOCSY, COSY, ROESY, NOESY, HSQC and HMBC spectra. The NMR data were processed using Bruker TOPSPIN 4/winNMR/UXNMR software. The ROESY spectra were acquired with tmix = 400 ms.

### 2.2. Extraction of Retinas

Retinas were extracted as previously described [35]. Briefly, eye semi-cups from freshly enucleated bovine eyes (from a local certified slaughterhouse) deprived of the anterior chamber and vitreous were incubated for 5 min with Mammalian Ringer (MR, 0.157 M NaCl, 5 mM KCl, 7 mM Na_2_HPO_4_, 8 mM NaH_2_PO_4_, 0.5 mM MgCl_2_, 2 mM CaCl_2_ pH 6.9) plus protease inhibitor cocktail (Sigma-Aldrich, St. Louis, MO, USA) and 50 μg/mL Ampicillin. The free-floating retinas were then collected.

### 2.3. Purified Bovine Rod OS Preparations and Treatments

Rod OS were isolated under red light at 4 °C from 20 bovine retinas by ultracentrifugation (one hour at 100,000× *g*) on a sucrose/Ficoll continuous gradient formed by a Light Medium (60 mM sucrose, 4% Ficoll *w*/*v*, 10 mM glucose, 10 mM ascorbic acid, 1 mM CaCl_2_, 20 mM Tris-HCl pH 7.4) and a Heavy Medium (20% sucrose *w*/*w*, 16% Ficoll *w*/*w*) [36,37]. The lower band corresponding to the sealed isolated OS was diluted with two volumes of 600 mM sucrose, 200 mM Tris-HCl pH 7.4, centrifuged at 5000× *g* for 20 min at 4 °C, collected, routinely characterized for integrity of plasma membrane [37] and stored at −80 °C. Before use, OS are homogenized by diluting the OS 1:4 (*v*/*v*) in ultrapure water (Milli-Q^®,^ Millipore, Billerica, MA, USA) and subjecting the suspensions to at least 10 passages through a needle (25 gauge) on ice. To induce an oxidative stress production in the samples, prior to the ATP synthesis assay, aliquots of purified OS were exposed to ambient light for 40 min, at 4 °C, in the presence of respiring substrates (0.6 mM NADH or 10 mM Na succinate) and ADP (0.1 mM). Purified OS aliquots kept at 4 °C in complete darkness treated in the same conditions were the corresponding controls.

### 2.4. Transmission Electron Microscopy

In order to perform double labelling experiments, bovine eye semi-cups (from a local certified slaughterhouse) were filled with 4% paraformaldehyde and 0.1% glutaraldehyde in PBS buffer (ON at 4 °C.). Semi-cups were then processed to remove the fixed retinas that were reduced into small pieces, dehydrated, embedded in LR White Resin and polymerized at 58 °C. For the post-embedding immunogold procedure, ultrathin sections were cut and treated on nickel grids with blocking solution (1% BSA, 0.1% Tween 20, PBS). Incubation was performed with mouse monoclonal anti-rhodopsin (1:100) (Sigma Aldrich, St. Louis, MO, USA) and rabbit polyclonal anti-ATP synthase β-subunit (diluted 1:50) (Sigma–Aldrich) overnight at 4 °C. As secondary antibodies, goat anti-mouse IgG (Sigma Aldrich) (diluted 1:100) coupled to gold particles (40 nm) and goat anti-rabbit IgG (Sigma Aldrich) (diluted 1:100) coupled to gold particles (10 nm) were used. Sections were analyzed at a FEI Tecnai G^2^ transmission electron microscope operating at 100 KV. To verify the labelling specificity, the preimmune serum was applied to the sections instead of the specific primary Ab. This control experiment resulted in absence of cross-reactivity (data not shown). Images were acquired with OSIS Veleta cameras, collected and typeset in Corel Draw X8.

### 2.5. ATP Synthesis Assay

ATP formation from ADP and inorganic phosphate was assayed in purified OS as previously described [36]. 100 µL OS aliquots (diluted to 1.8 mg/mL) were preincubated with either 1.0 mg/mL cirsiliol or the same volume of vehicle for controls (DMSO) for 1 h in the dark at 25 °C. Then, aliquots of suspensions were incubated for 5 min at 37 °C in 50 mM Tris-HCl pH 7.4, 50 mM KCl, 1 mM EGTA, 2 mM MgCl_2_, 0.6 mM ouabain, 0.25 mM di(adenosine)-5-penta-phosphate (Ap5A, adenylate kinase inhibitor) and 25µg/mL ampicillin to a final protein concentration of 0.012 mg protein/0.1 mL, plus respiratory substrates (0.6 mM NADH and 10 mM Na succinate). ATP synthesis was induced by addition of 5 mM KH_2_PO_4_ and 0.1 mM ADP at the same pH of the mixture and ATP formation, followed for 1 min in a luminometer (GloMax^®^ 20/20, Promega Corp. Madison, Fitchburg, WI, USA) by the luciferin/luciferase chemiluminescent method (Roche Diagnostics Corp., Indianapolis, IN, USA). Calibration curve was obtained with ATP standard solutions (Roche Diagnostics Corp., Indianapolis, IN, USA) between 10^−9^ and 10^−7^ M in the same solution as the experiments.

### 2.6. Assay of the ETC Complexes

ETC complexes I, II, III and IV were assayed as previously described [38,39], either in coupled or uncoupled conditions, the latter obtained by adding 0.01 mM nigericin plus 0.01 mM valinomycin. In all cases, OS homogenate aliquots (1.8 mg/mL) were preincubated with either 1.0 mg/mL cirsiliol or the same volume of vehicle for controls (DMSO) for 1 h in the dark at 25 °C. When necessary, uncoupling was conducted.

Then, suspensions were utilized for the assays:

Complex I (NADH-ubiquinone oxidoreductase) activity was measured spectrophotometrically on 0.04 mg/mL of total OS homogenate protein, following the reduction of ferricyanide at 420 nm, considering ε_mM_ for FeCN^-^ = 1.0 M^−1^⋅cm^−1^. The assay medium contained: 50 mM Tris–HCl pH 7.4, 50 mM KCl, 5 mM MgCl_2,_ 1 mM EGTA 0.6 mM NADH, 0.8 mM ferricyanide and 50 µM Antimycin A.

Complex II (succinate dehydrogenase) activity was measured spectrophotometrically on 0.04 mg/mL of total OS homogenate protein, following the reduction of ferricyanide at 420 nm, considering ε_mM_ for FeCN^-^ = 1.0 M^−1^⋅cm^−1^. The assay medium contained: 50 mM Tris–HCl pH 7.4, 50 mM KCl, 5 mM MgCl_2_ 1, mM EGTA, 10 mM succinate, 0.8 mM ferricyanide and 50 µM Antimycin A.

Complex III (cytochrome *c* reductase) activity was measured spectrophotometrically on 0.04 mg/mL of OS homogenate protein, following the reduction of oxidized cytochrome c (Cyt *c*) at 550 nm, considering ε_mM_ for Cyt *c* = 20 M^−1^⋅cm^−1^. The assay medium contained: 50 mM Tris-HCl pH 7.4, 5 mM KCl, 2 mM MgCl_2_, 0.5 M NaCN, 0.03% oxidized Cyt *c*, 0.6 mM NADH, 20 mM succinate.

Complex IV (cytochrome *c* oxidase) activity was measured spectrophotometrically on 0.04 mg/mL of OS homogenate protein, following the oxidation of 0.3 mM Cyt *c* (reduced by diluting it in a 0.1 mM Na ascorbate solution) at 550 nm, considering ε_mM_ for Cyt *c* = 20 M^−1^⋅cm^−1^. The assay medium contained: 50 mM Tris-HCl pH 7.4, 5 mM KCl, 2 mM MgCl_2_, 0.03% oxidized Cyt *c*, 0.2 mM NADH, 20 mM succinate.

### 2.7. Cytofluorimetric Assay

H_2_O_2_ production by the OS was assessed by flow cytometric analysis. The OS homogenate preparations were first incubated for one hour with 1 mg/mL cirsiliol or the same volume of DMSO (used as control) for 1 h at room temperature. Then, aliquots of suspensions were adjusted to 0.05 mg/mL of protein by adding 10 mM HEPES, 135 mM NaCl, 5 mM CaCl_2_, 2 mM MgCl_2_ and exposed to light to elicit the light-dependent development of oxidative stress [39], which were stained with 2′,7′-dichloro-dihydrofluorescein diacetate (H_2_DCFDA) 5 μM for 15 min. Samples were treated with 0.1 mM ADP, plus 0.2 mM NADH and 10 mM succinate as respiring substrates, just before measurements on a FacsCalibur flow cytometer (Becton Dickinson, Milan, Italy) equipped with a 488 nm laser for proper H_2_DCFDA excitation. The OS-induced forward (FSC) and side (SSC) laser light scattering signals were used to identify structurally undamaged OS and separate them from degraded OS and debris.

### 2.8. Molecular Modeling

A molecular docking analysis was performed to evaluate the feasible binding geometries of cirsiliol with the known three-dimensional structure of the F_1_ moiety of ATP synthase [8]. Two different docking programs were chosen for the analysis: Autodock Tools v1.5.7 ADT program and FRED (Fast Rigid Exhaustive Docking) of OpenEye suite [40,41].

The NCBI PubChem SMILES code for cirsiliol (https://pubchem.ncbi.nlm.nih.gov/compound/Cirsiliol accessed on 8 August 2022) was converted into Protein Data Bank (PDB) format with the *ad-hoc* converter from NIH (https://cactus.nci.nih.gov/translate/ accessed on 8 August 2022). Bovine mitochondrial F1-ATP synthase three-dimensional structure, solved by X-ray crystallography by Gledhill et al. [8], was retrieved from the Protein Data Bank (PDB) database (https://www.rcsb.org accessed on 8 August 2022), (search terms 2JIZ and 2JJ2). To define the correct ionization and tautomeric states of each amino acid residue, we removed the ligand and added the H-atoms to the ATP synthase molecular structure. Steric clashes were alleviated using the suitable force field in a correction of the missing side chains and in a minimization of the protein structure.

The three-dimensional grid for mapping the ligand on the outer and inner surface was generated by autogrid4 of ADT program using all default parameters. The grid box consisted of 126 points for each dimension and has centered on the binding site of quercetin (adjustment parameters: X= −7.005; Y= 25.913; Z= 9.847), with a resulting resolution of 0.375 Å. Autodock4 v1.5.7 software was used for the analysis, setting the Lamarckian genetic algorithm for the flexible ligand–receptor docking step. The results were displayed with the same program, and the images were reprocessed using USCF Chimera.

A systematic examination of all possible protein–ligand conformations was then performed with FRED. Filters for shape complementarity and custom restraints were added using SPRUCE and MakeReceptor tools, generating a design unit of volume ca 4000 Å^3^ centered on the ligand position. Considering all tautomers and chiralities for all the ionization states, 16 possible conformations of the potential ligand cirsiliol were prepared involving OMEGA, as implemented in with the OpenEye suite programs. Selection and optimization of the final poses was performed using the Chemgauss4 scoring function [41], and then, thanks to the Grapheme toolkit, a detailed scoring analysis was provided.

### 2.9. Statistical Analysis

The statistical significance was tested using a two-tailed Student’s *t*-test. Statistical tests were performed using Prism (GraphPad 9.4.1 software). Data are expressed as mean ± SD, and *p* scores are indicated as * *p* < 0.05, ** *p* < 0.01 and **** *p* < 0.0001 and ns for not significant (*p* > 0.05).

## 3. Results

### 3.1. Cirsiliol Binds the F_1_F_o_-ATP Synthase F1 Moiety

**Figure 1:**
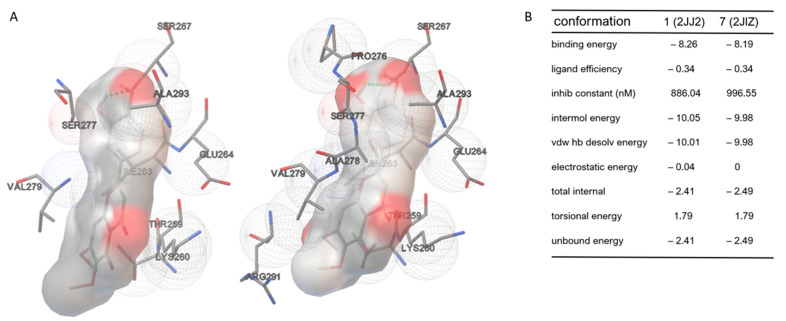
Molecular docking analysis of cirsiliol on F_1_ moiety of the F_1_F_o_-ATP synthase (ATP synthase). (**A**) Molecular docking (left: conformation 1 using 2JJ2 (see also Supplementary Video S1); right: conformation 7 using 2JIZ (see also Supplementary Video S2)). Both conformations displayed hydrogen bonds (highlighted as green dots) with Ser-267.G. (**B**) Energy parameters assessed for conformations bearing the highest binding energy. The data show the elevated inhibition constants.

In consideration of its polyphenolic nature, an in silico molecular docking analysis of the binding of cirsiliol to ATP synthase was conducted. We asked whether this polyphenol could also directly interact with the molecular structure of the F1 subunit of ATP synthase, similar to resveratrol and quercetin [8]. Molecular docking analyses with Autodock revealed a strong interaction of cirsiliol with the target enzyme. Figure 1A,B (and Supplementary Videos S1 and S2) show the two conformations with the higher binding energy, and the side table summarizes the energy parameters assessed. The ligand molecule is bound in a slightly distorted planar conformation by means of hydrophobic interactions and H-bonds that involve mainly the residues of Lys-260.G, Ile-263.G, Ser-277.F, Val-279.F and Ser-267.G. Moreover, the side-chain regions of Thr-259.G, Glu-264.G, Arg-291.B and Ala-293.C appear to contribute to the interaction.

Considering its flexibility, using the FRED tool [41], we further explored the different possible conformations of cirsiliol fitting into the established binding site (see Supplementary Data S1 and S2). The calculated energy for the assessed conformations makes the molecular docking simulations more reliable, supporting that cirsiliol binds the F1 moiety of ATP synthase.

Cirsiliol binds to ATP synthase in a similar way as quercetin and resveratrol (Supplementary Figure S1). Superimposition of the inhibitor binding sites in the three structures shows that the chains involved in the binding are the same, and most of the interacting amino acids also recur in the binding of cirsiliol. Even when enlarging the grid considered for subsequent molecular docking calculations, cirsiliol was preferentially positioned where the two other polyphenols were found in the crystallographic structure.

### 3.2. Rhodopsin and the β-Subunit of ATP Synthase Colocalize in the Rod Outer Segment

**Figure 2:**
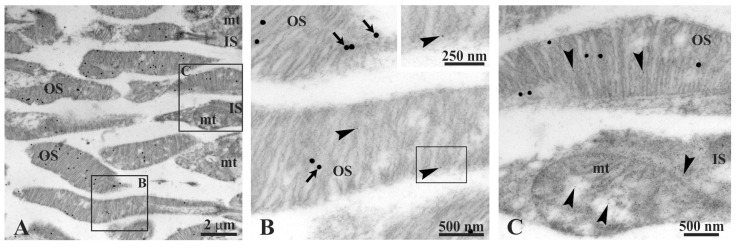
TEM of bovine retina. (**A**) The retina exhibits photoreceptorinner (IS) and outer (OS) segments. Squared areas B and C are enlarged in panels B and C, respectively. (**B**) Part of three OS belonging to close photoreceptors. Largest gold particles (40 nm width, arrows) reveal the Ab against anti-rhodopsin; smaller gold particles (10 nm width, arrowheads) evidence the Ab against anti-ATP synthase β-subunit. A gold particle is enlarged in inset. (**C**) Details of an IS close to a OS. Note the numerous small gold particles in the mitochondrion (mt) of the IS. In the OS, two small gold particles are recognizable.

Experiments of double immunogold labelling were performed on retinal sections at TEM in order to evidence the colocalization of rhodopsin and ATP synthase in OS. The colocalization was clearly recognizable using antibodies (Ab) against rhodopsin (Rh) (40 nm diameter gold particles) and β-subunit of ATP synthase (10 nm diameter gold particles) (Figure 2). Meanwhile, large gold particles, detecting the Ab against rhodopsin, were spread exclusively on OS; small gold particles, showing the Ab against ATP synthase, were easily found in mitochondria of IS and in OS. In Figure 2, panel A shows a typical retina field, with portions of numerous OS and IS. Panels B and C allow recognition of numerous small gold particles (Ab against ATP synthase); note that they are very numerous in an IS mitochondrion (Figure 2C).

### 3.3. F_1_F_o_-ATP Synthase Activity in Rod OS

**Figure 3:**
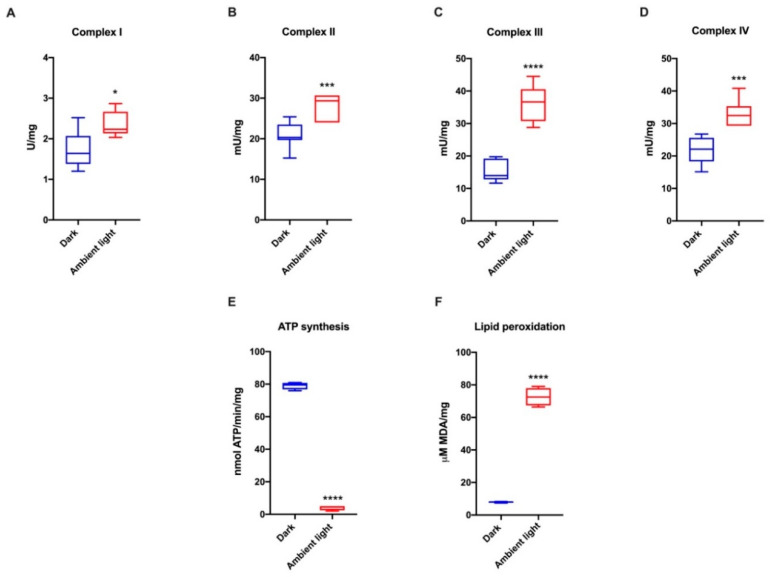
Respiratory complexes activity, ATP synthesis and lipid peroxidation in the rod OS. All experiments were carried out on rod OS maintained either in the dark or in ambient light in the presence of 0.6 mM NADH, 10 mM succinate and 0.1 mM ADP. (**A**–**D**) Respiratory complexes activity. (**E**) ATP synthesis. (**F**) MDA levels as a lipid peroxidation marker. Data are representative of at least three independent experiments. *, *** and **** indicate a significant difference for *p* < 0.05, 0.001 and 0.0001, respectively, between the rod OS kept in the dark or in ambient light.

To evaluate the effect of light exposure on the OxPhos, the respiratory complexes activity, ATP synthesis and lipid peroxidation were evaluated in rod OS incubated prior with respiratory substrates and ADP in the dark or exposed for 30 min to ambient light, plus substrates and ADP. The data show that the activity of respiratory complexes increased in the rod OS treated with ambient light compared to those kept in the dark (Figure 3A–D). Conversely, ATP synthesis was impaired in the rod OS treated with light (Figure 3E). This apparent discrepancy could be explained considering that the light exposure in the presence of substrates and ADP could cause overworking of OxPhos and relative oxidative stress production. This hypothesis appears confirmed by the lipid peroxidation increment observed in the same conditions (Figure 3F). On the other hand, uncoupled conditions are characterized by a decrement in ATP synthesis due to proton gradient dissipation and the increment of respiratory complexes activity no more under the control of ATP synthase.

### 3.4. Cirsiliol Inhibits OS ATP Synthesis

**Figure 4:**
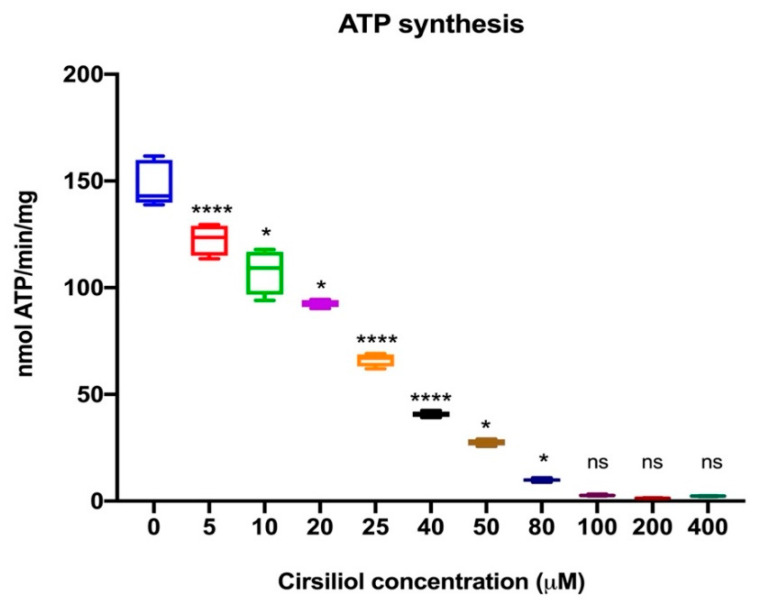
Effect of cirsiliol on F_1_F_o_ -ATP synthase activity in rod OS. ATP synthesis in the rod OS after preincubation of the sample with the indicated cirsiliol concentrations (see Materials and Methods section). Data are representative of at least three independent experiments and are indicated as mean ± SD. * and **** indicate a significant difference for *p* < 0.005 or *p* < 0.0001, respectively, between the subsequent samples. ns indicates a non-significant statistical difference.

Considering that the molecular docking analysis suggests a direct interaction between cirsiliol and ATP synthase, we evaluated its effect on ATP production, assayed by luminometry, by the OS homogenates. As reported in Figure 4, cirsiliol induced a decrement in ATP production in the presence of NADH and succinate as respiring substrates to fuel both Complex I–III–IV and Complex II–III–IV pathways (*p* < 0.001). Figure 4 shows a significant dose-dependent inhibition operated by cirsiliol towards ATP synthesis.

### 3.5. Cirsiliol Inhibits the Activity of the OS Respiratory Complexes Exclusively When in Coupled Conditions

Since the ETC is the main source of oxidative stress production, the effect of cirsiliol on the activity of the four respiratory complexes of the electron transfer chain (ETC) ectopically expressed in the rod OS was evaluated.

**Figure 5:**
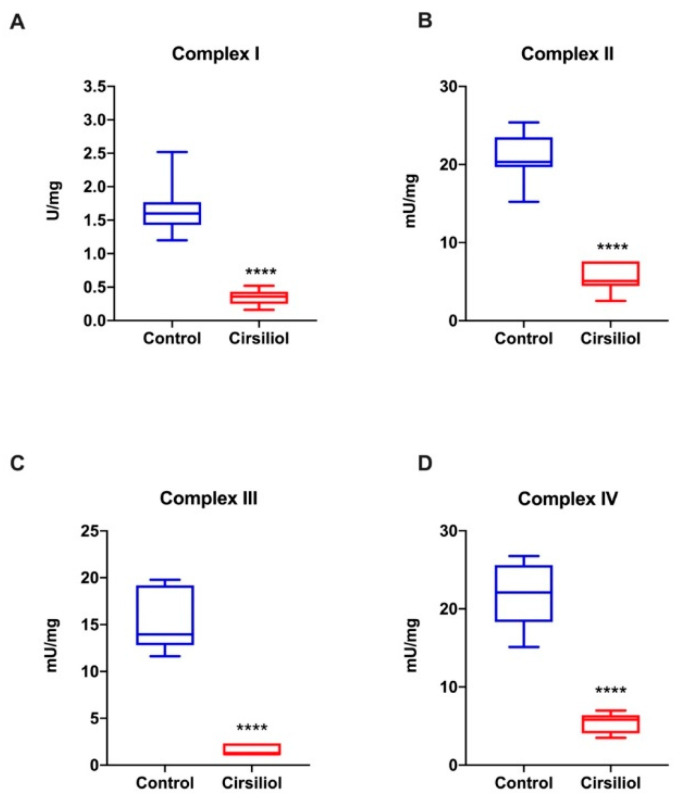
Effect of cirsiliol on the respiratory complex activity in the rod OS in coupled conditions. Figure shows the activity of the four respiratory complexes. Panels (**A**–**D**) report the activity of Complexes I to IV in coupled conditions in the absence or in the presence of cirsiliol. Data are from *n* = 5 independent experiments and are expressed as mean ± SD. Results show a non-statistically significant difference in activity. **** indicates a significant difference for *p* < 0.0001 between treated and untreated samples in coupled conditions.

As reported in Figure 5A–D, all respiratory complexes were inhibited by the cirsiliol treatment.

**Figure 6:**
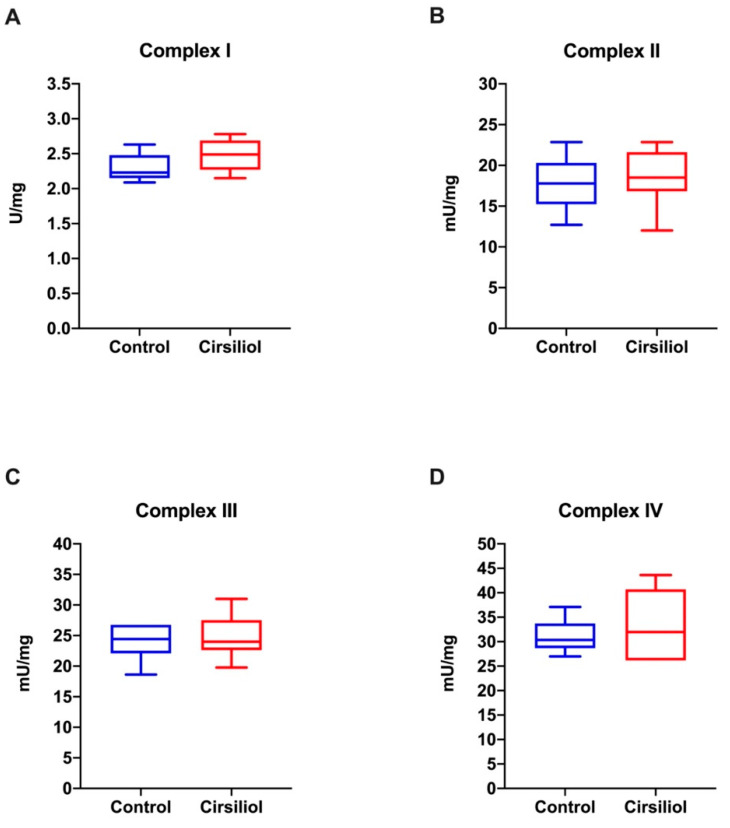
Effect of cirsiliol on the respiratory complex activity in the rod OS in uncoupled conditions. Figure shows the activity of the four respiratory complexes in the absence or in the presence of cirsiliol. Panels (**A**–**D**) report the activity of Complexes I to IV in uncoupled conditions (i.e., after addition of 0.01 mM nigericin plus 0.01 mM valinomycin) in the absence or in the presence of cirsiliol. Data are expressed as mean ± SD and are from *n* = 5 independent experiments. A non-statistically significant difference is observed.

By contrast, the inhibitory effect of cirsiliol on ETC activity is undetectable in uncoupled conditions (Figure 6A,B), suggesting that the effect on the ETC depends on ATP synthase inhibition. In other words, there would not be a direct interaction between cirsiliol and respiratory complexes.

### 3.6. Cirsiliol Decreased Reactive Oxygen Intermediates Production in the Light-Exposed Rod OS, Lowering the Oxidative and Structural Damage

**Figure 7:**
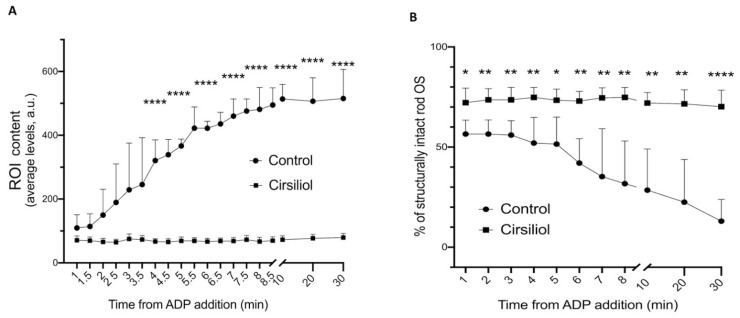
Effect of cirsiliol on H_2_O_2_ production and rod OS integrity. (**A**) H_2_O_2_ production in rod OS treated (black squares) or untreated (black circles) with 50 μM cirsiliol during 40 min of ambient light exposure. (**B**) Percentage of structure integrity of rod OS in the same condition of Panel A. *, **, **** indicate a significant difference for *p* < 0.05, 0.01, 0.0001, respectively, between treated and untreated samples in coupled conditions. Data are expressed as mean ± SD.

The ETC expressed in the rod OS disks produces a considerable amount of free radicals in vitro when the sample is exposed to light in conditions favoring OxPhos functioning, i.e., in the presence of respiring substrates and ADP [16,17]. Thus, to evaluate an antioxidant effect of cirsiliol, a flow cytometric analysis was conducted on intact rod OS utilizing an H_2_DCFDA probe to evaluate the H_2_O_2_ concentration. Cirsiliol inhibited the progressive production of H_2_O_2_ consequent to 40 min ambient light exposure (Figure 7A). Controls were kept in the dark for the same length of time. Furthermore, the instrument was also able to detect the percentage of intact OS. Interestingly, cirsiliol also protected from oxidative-stress-mediated progressive degradation of rod OS without cirsiliol, whose integrity was compromised, as highlighted by the preservation of structural integrity when rod OS were pretreated with cirsiliol (Figure 7B).

## 4. Discussion

The present study evaluated binding of flavone cirsiliol to ATP synthase by molecular docking analysis. This compound was chosen in consideration of its polyphenolic nature [8] and the effect of such a specific interaction on the OxPhos, utilizing the rod OS as an experimental model. Cirsiliol was extracted from *S. x jamensis*. The rod OS disk sidedness is similar to that of the inverted mitochondrial vesicles, as demonstrated by previous fluorescence confocal microscopy imaging, suggestive of the fact that the latter is accessible to the OS cytosol [13]. In fact, as confirmed by the double immunogold labelling experiment at TEM, an ectopic ETC and ATP synthase are expressed in the OS disk membranes [11,12,13]. It was shown that the ATP synthase is ectopically expressed on the plasma membrane of some normal cell types, such as hepatocytes [42], endothelial cells [43] and adipocytes [44], but also in myelin sheath [45], exosomes [46] and microvesicles [47]. In tumor cells, ATP synthase ectopic expression on the plasma membrane [48] is proportional to the malignancy potential of cell types [49,50]. The mechanism by which the ATP synthase and ETC are transferred from the inner mitochondrial membrane to their ectopic locations has not yet been established [51].

The in silico docking data show that cirsiliol binds the ATP synthase F_1_ moiety in a similar way as shown for resveratrol and quercetin. Docking analysis was also conducted on TYK2, and it was found that cirsiliol binds the protein with an equilibrium dissociation constant (KD) of 0.8 μM and a binding affinity increasing with time and concentration, inhibiting it [32]. Interestingly, cirsiliol binds in the ATP binding pocket of the kinase [32].

Besides the docking data, the results of the biochemical analyses also suggest that the actual target of cirsiliol is ATP synthase; in fact, cirsiliol inhibited OS ATP synthesis in a dose-dependent manner and apparently inhibited the four ETC complexes, but only in a coupled condition, while, in the presence of nigericin and valinomycin (uncoupled conditions), cirsiliol was unable to affect the ETC. Other studies have also reported inhibitory action of polyphenols on ATP synthase activity [8,52]. The results reported here show that cirsiliol extracted from *S. x jamensis* lowers both H_2_O_2_ production in the OS from exposure to sustained ambient light and the massive structural OS damage due to oxidation. In the OS, the aerobic metabolism appears linked to light absorption, suggestive of the fact that the former plays a key role in the production of chemical energy for the phototransduction process [53]. The results confirm the ability of OS to self-oxidate upon light exposure. Excess oxidative stress production in OS is driven in the presence of respiring substrates and ADP, with light acting as a “switch” [17], as shown by the overproduction of MDA in light (Figure 3B). The loss of ATP synthetic ability by the OS exposed to saturating ambient light in the presence of respiratory substrates and ADP, as compared to dark-adapted OS, may be due to an uncoupling of the ATP synthase from the ETC, as suggested by the increased oxidative stress production and respiratory complexes activity. Conceivably, in vitro, the OS would produce excess oxidative stress upon exposure to light as they lose the dioptric media of the eye and the physiological antioxidant concentrations. In fact, the ETC is a main source of oxidative stress [54].

It was shown for the first time in this study that oxidative stress causes considerable structural damage to OS in vitro. Overworking of oxidative phosphorylation due to the incremental ATP demand from phototransduction would result in oxidative production. Similar results were obtained by exposure of eye cultures ex vivo to blue light (405 nm) for 6 h [55], which resulted in oxidative damage higher in the rod OS than the IS [56].

Notably, our experimental model also represents the portion of a photoreceptor foremostly involved in phototransduction [15]. Therefore, the study of the mechanism of oxidative stress production in this model is significant for the field of the study of retinopathies and degenerative retinal damage in consideration of the known pathogenic role of oxidative stress as a triggering factor related to arising of AMD [57,58], DR [59] and especially rod-driven retinopathies [60]. The end products of lipid oxidation, such as 4-hydroxynonenal (4-HNE), are found primarily in OS [55,61]. Notably, photoreceptors consume about four times more O_2_ than the other central nervous system tissues on a weight basis [62].

In the spectrum of the multiple and diversified effects of flavones, the present data uphold cirsiliol’s anti-radical and antioxidant effects by reducing free radical production by the ETC of rod OS following light exposure of the membranes in the presence of respiring substrates and ADP. The examined compound operates a significant dose-dependent inhibition of the production of ATP by binding and inserting itself among the α and β subunits of the ectopic ATP synthase F_1_ moiety. Thus, it induces a temporary and transitory deceleration in the rotational kinetics of the nano enzyme, slowing down the entire OxPhos process and modulating the general inflammatory state of the biological model. Notwithstanding the current need to investigate deeper cirsiliol bioavailability and interaction with other polyphenols and micronutrients, it appears appropriate to suggest its implementation in nutritional plans and food supplements through plant-based food (*Salvia* spp., *Aloysiatriphylla*, *Leonotislepetifolia* and others) or nutraceutical preparations able to supply it.

## 5. Conclusions

The data show that, similarly to other polyphenols [8], cirsiliol specifically binds the ATP synthase with a high binding energy. The effect of this inhibition is advantageous as it lowers the free radical production by the ectopic OS ETC, limiting the detrimental effect of oxidation to the structure of the rod OS rich in polyunsaturated fatty acids (PUFA). These results are suggestive of the possibility of protecting the whole retina from oxidative damage triggering retinal degenerative diseases [16] by taking advantage of polyphenols. The data also suggest that ATP synthase may be one of multiple molecular targets of the antioxidant action of *Salvia* spp., establishing the basis for development of promising plant-based drugs.

## Data Availability

The analyzed data supporting the conclusions of this article are included within this article and its additional files.

## Supplementary Materials

The following are available online at https://www.mdpi.com/article/10.3390/cells11193169/s1, Figure S1: Comparison of the interacting amino acids in the binding of the three different polyphenols with bovine F1 moiety of ATP synthase. Video S1: Molecular docking analysis of cirsiliol on F1 moiety of ATP synthase using 2jj2; Video S2: Molecular docking analysis of cirsiliol on F1 moiety of ATP synthase using 2jjz. Data S1: FRED tool data using 2jj2; Data S2: FRED tool data using 2jjz.

## Abbreviations

Ap5A: di(adenosine)-5-penta-phosphate; ATP synthase: F_1_F_o_-ATP synthase; ETC: electron transport chain; OS: outer segment; OxPhos: oxidative phosphorylation; TEM: transmission electron microscopy.
