# Supplementary material for: The Flavone Cirsiliol from Salvia x jamensis Binds the F1 Moiety of ATP Synthase, Modulating Free Radical Production
Source: Cells. 2022 Oct 9;11(19):3169. doi: 10.3390/cells11193169 (PMC9562182; doi:10.3390/cells11193169)

Molecule Name	omega_1_16
Molecular Weight	330.3
XLogP	1.2
PSA	109.4
Heavy Atoms	24
Acceptor Count	7
Donor Count	3
Chelator Count	3
Pose Index	1

Total Score -11.29

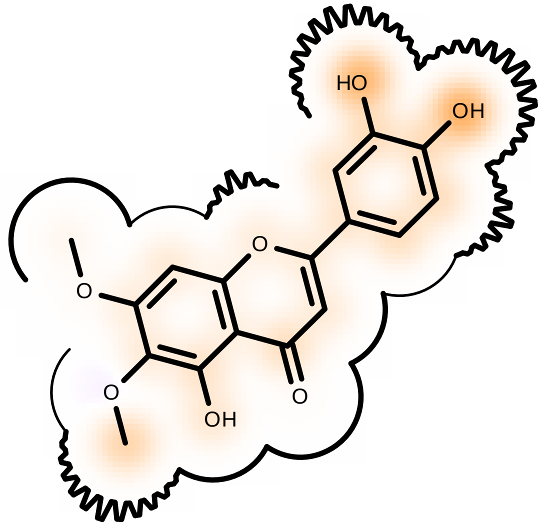

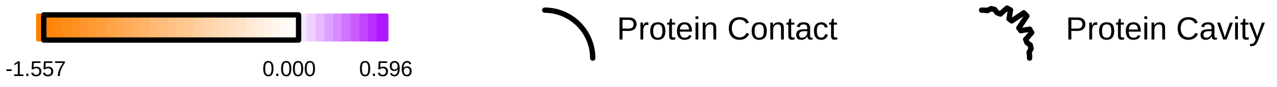

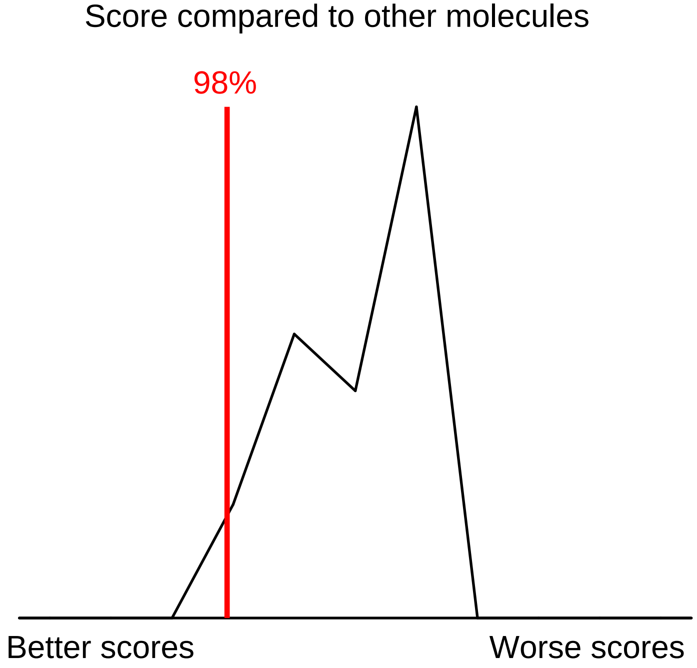

Residue Fingerprint

ALA256G	ALA278E
ALA278F	ALA293B
ALA293C	ALA336B
ARG252G	ARG291B
ASP316F	ASP333B
GLN255G	GLU264G
GLU292B	GLU292C
GLY290B	ILE263G
LYS260G	THR259G
VAL279E	VAL279F

Shape -12.36

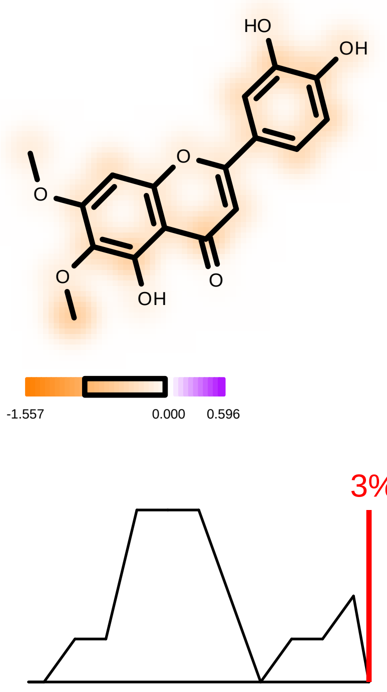

Hydrogen Bond -4.51

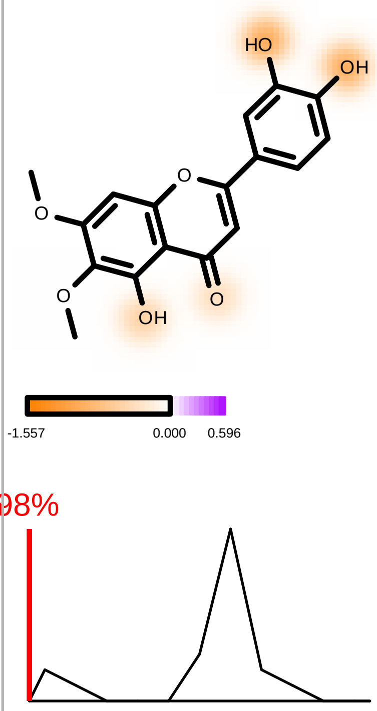

Protein Desolvation 3.09

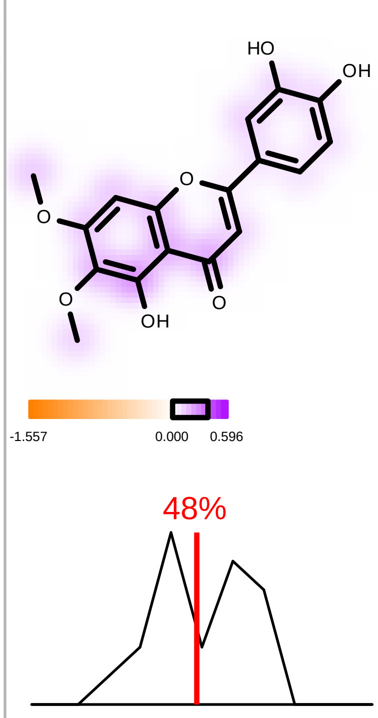

Ligand Desolvation 2.49

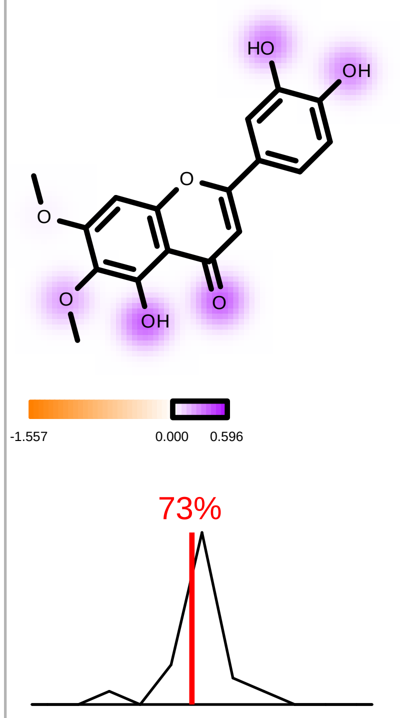

Acceptor	Donor
Metal	Contact

Molecule Name	omega_1_10
Molecular Weight	330.3
XLogP	1.2
PSA	109.4
Heavy Atoms	24
Acceptor Count	7
Donor Count	3
Chelator Count	3
Pose Index	2

Total Score -11.24

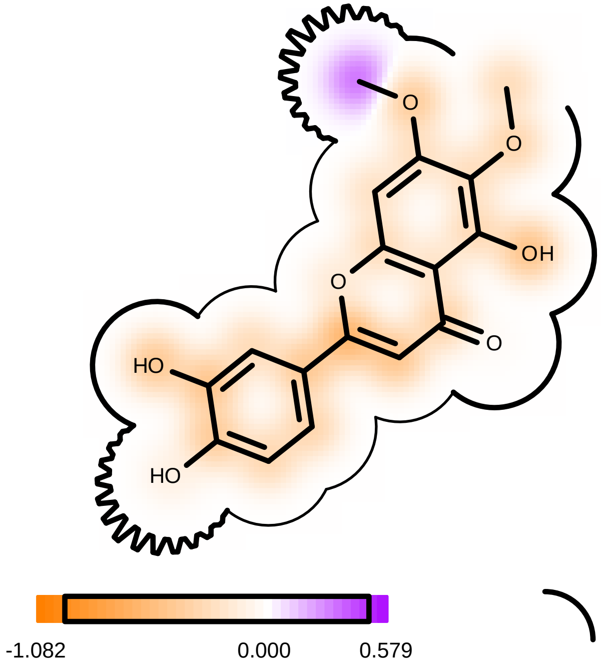

Score compared to other molecules

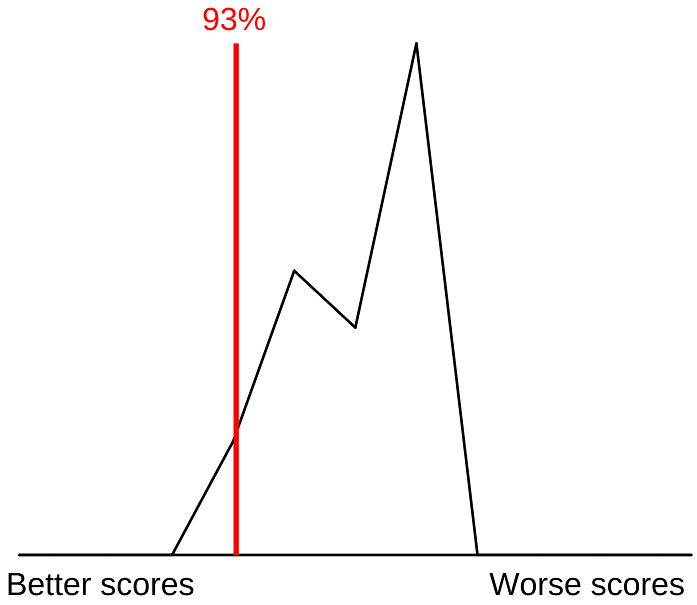

Protein Contact

Protein Cavity

Residue Fingerprint

ALA256G	ALA278E
ALA278F	ALA293B
ALA293C	ALA336B
ARG252G	**ARG291B**
ASP316F	ASP333B
GLN255G	**GLU264G**
**GLU292B**	GLU292C
GLY290B	ILE263G
LYS260G	THR259G
VAL279E	VAL279F

Acceptor	Donor
Metal	Contact

Shape -14.64

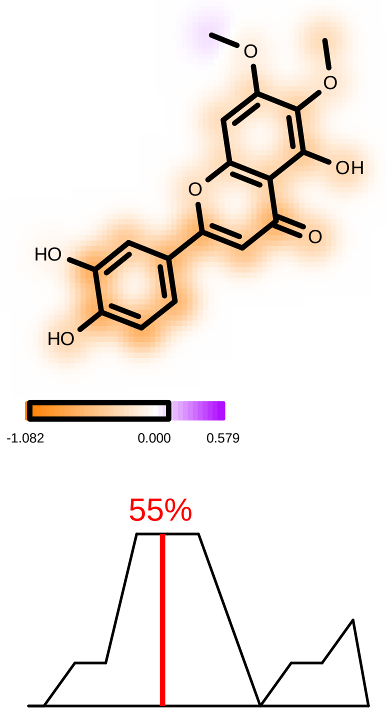

Hydrogen Bond -3.50

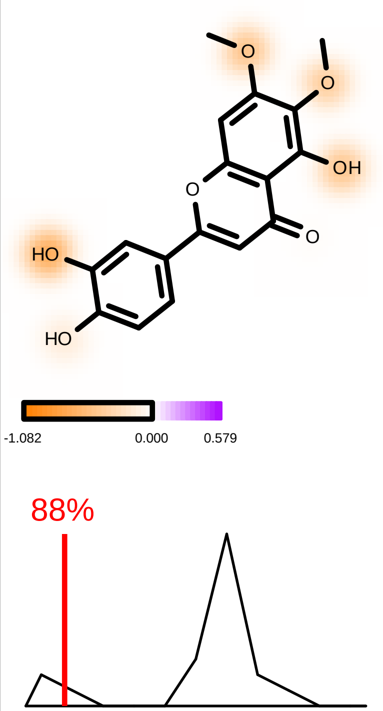

Protein Desolvation 3.91

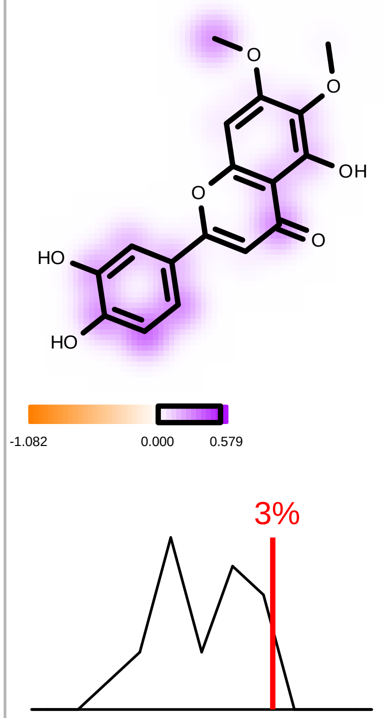

Ligand Desolvation 2.99

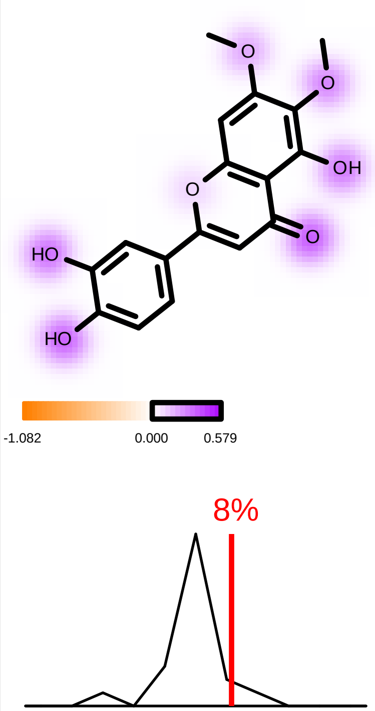

Molecule Name	omega_1_4
Molecular Weight	330.3
XLogP	1.2
PSA	109.4
Heavy Atoms	24
Acceptor Count	7
Donor Count	3
Chelator Count	3
Pose Index	3

Total Score -10.92

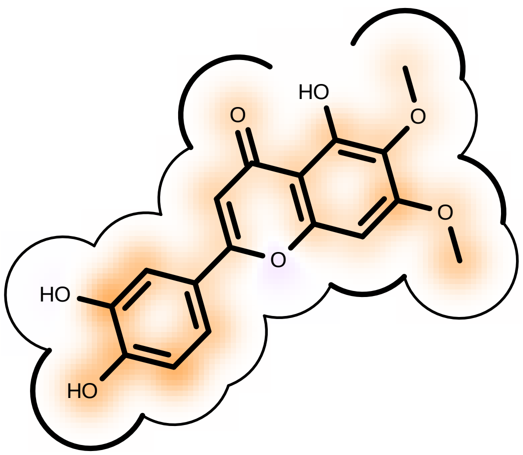

Score compared to other molecules

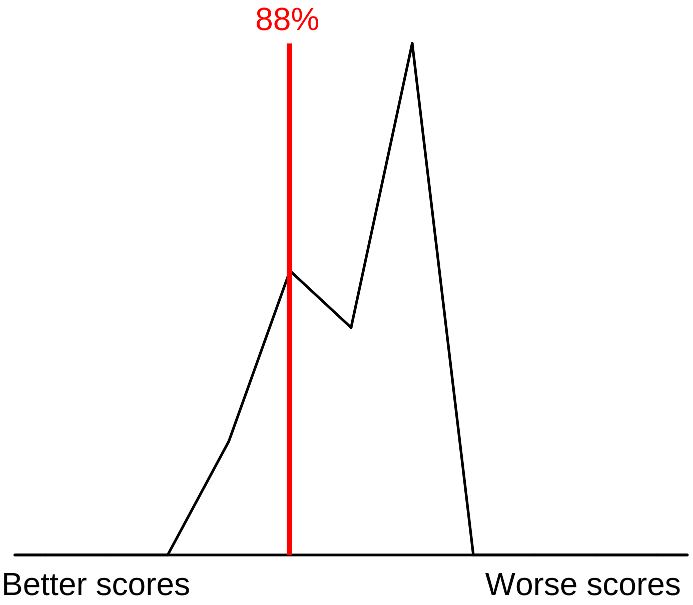

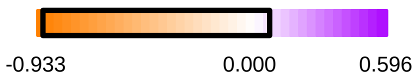

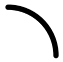 Protein Contact

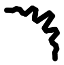 Protein Cavity

Residue Fingerprint

ALA256G	ALA278E
ALA278F	ALA293B
ALA293C	ALA336B
ARG252G	ARG291B
ASP316F	ASP333B
GLN255G	GLU264G
GLU292B	GLU292C
GLY290B	ILE263G
LYS260G	**THR259G**
VAL279E	**VAL279F**

Acceptor	Donor
Metal	Contact

Shape -14.71

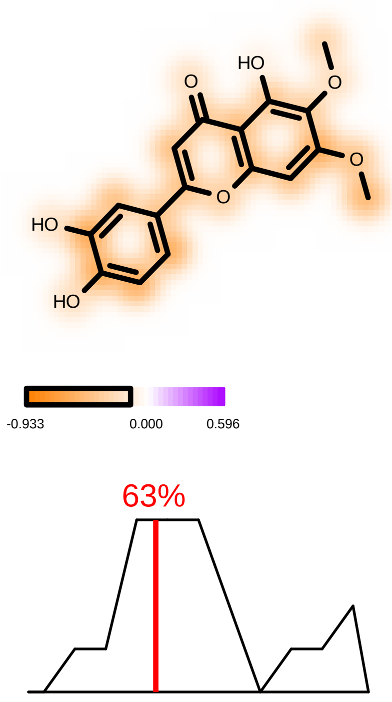

Hydrogen Bond -1.59

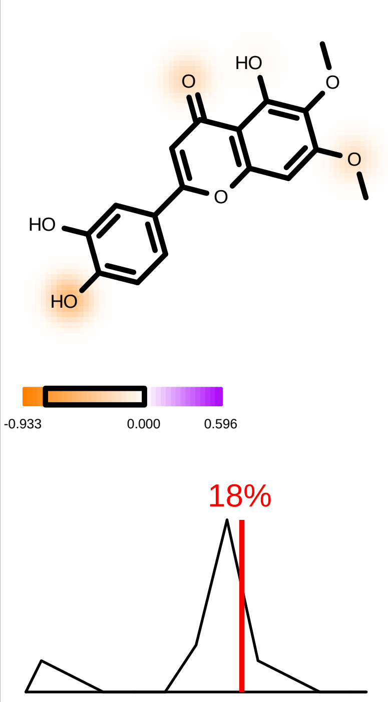

Protein Desolvation 2.93

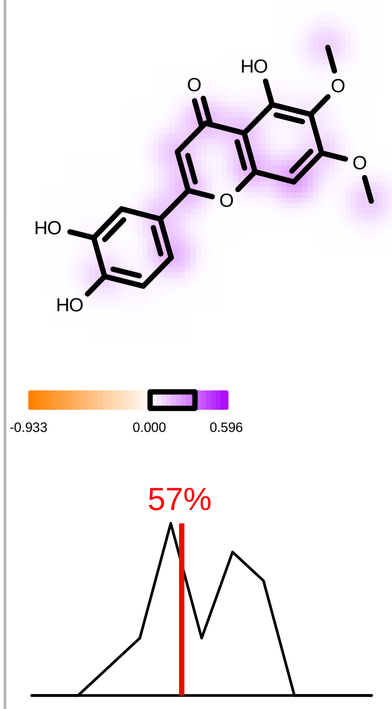

Ligand Desolvation 2.45

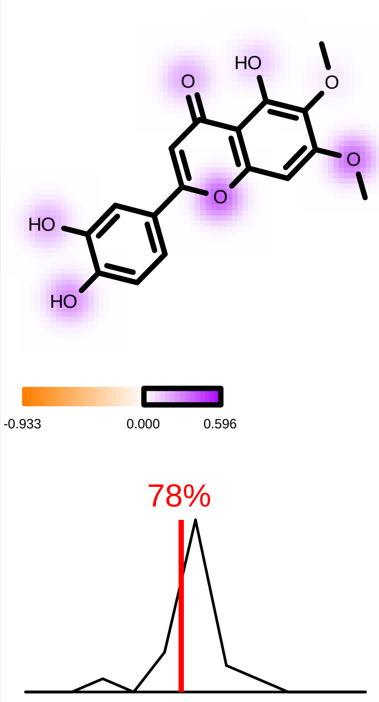

Molecule Name	omega_1_13
Molecular Weight	330.3
XLogP	1.2
PSA	109.4
Heavy Atoms	24
Acceptor Count	7
Donor Count	3
Chelator Count	3
Pose Index	4

Total Score -10.90

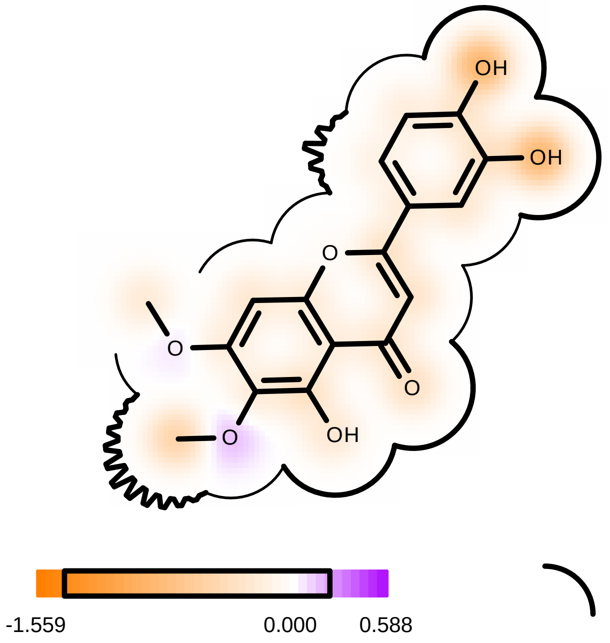

Score compared to other molecules

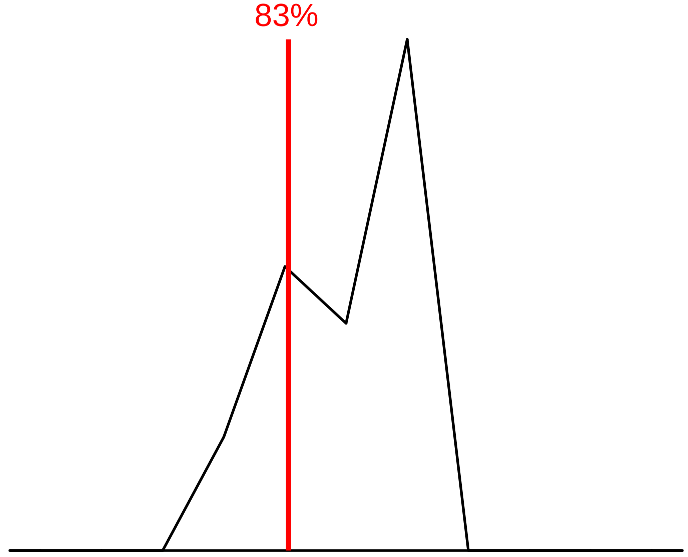

Residue Fingerprint

ALA256G	ALA278E
ALA278F	ALA293B
ALA293C	ALA336B
ARG252G	ARG291B
ASP316F	ASP333B
GLN255G	GLU264G
GLU292B	GLU292C
GLY290B	ILE263G
LYS260G	THR259G
VAL279E	VAL279F

Acceptor	Donor
Metal	Contact

Shape -12.59

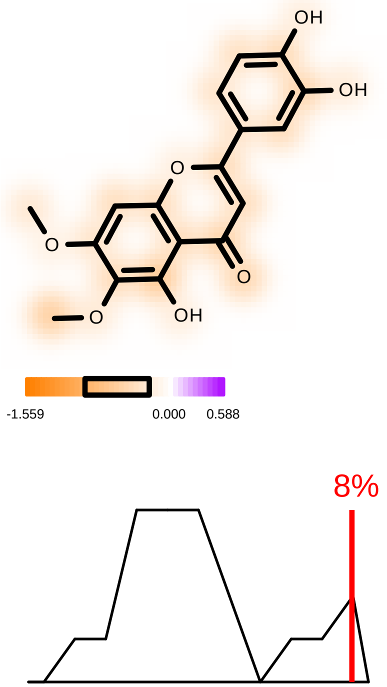

Hydrogen Bond -4.19

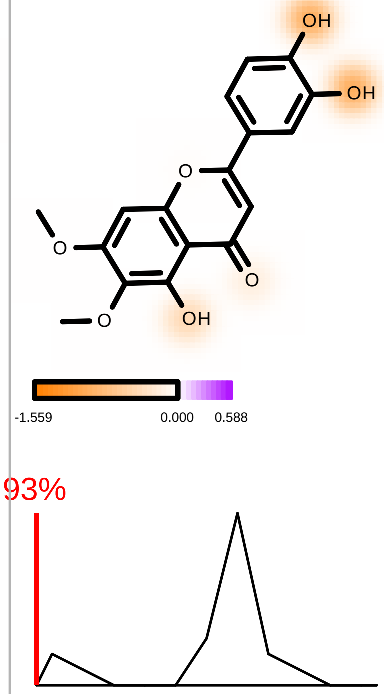

Protein Desolvation 2.75

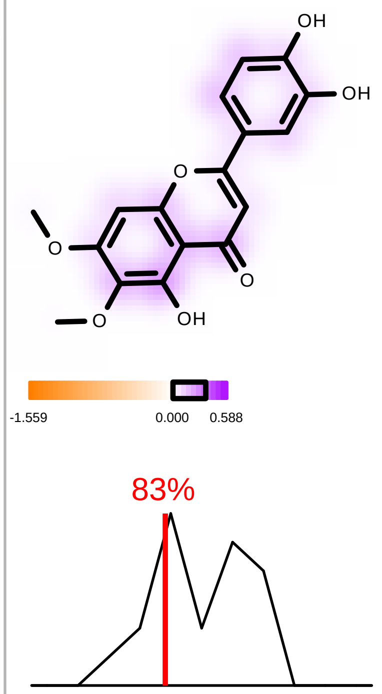

Ligand Desolvation 3.13

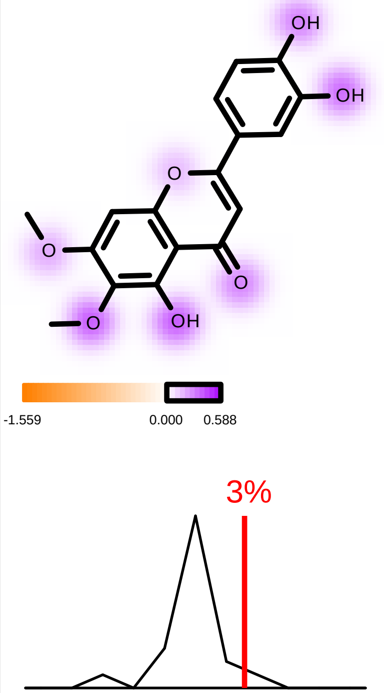

Molecule Name	omega_1_14
Molecular Weight	330.3
XLogP	1.2
PSA	109.4
Heavy Atoms	24
Acceptor Count	7
Donor Count	3
Chelator Count	3
Pose Index	5

Total Score -10.80

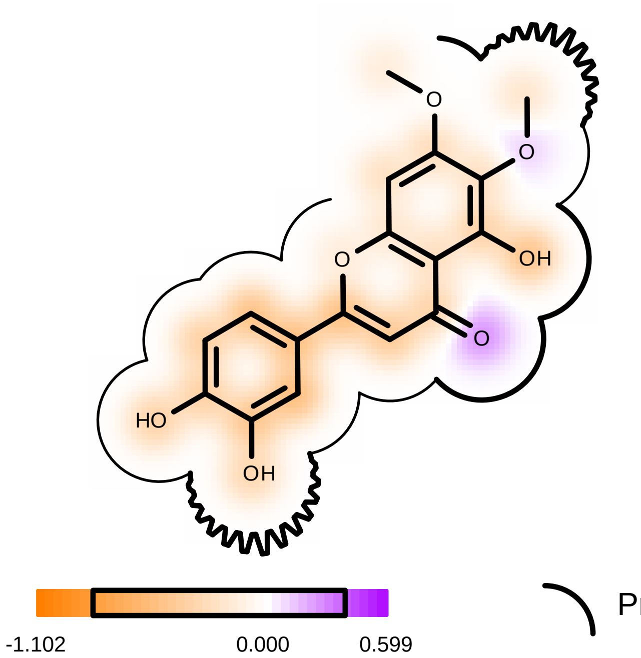

Score compared to other molecules

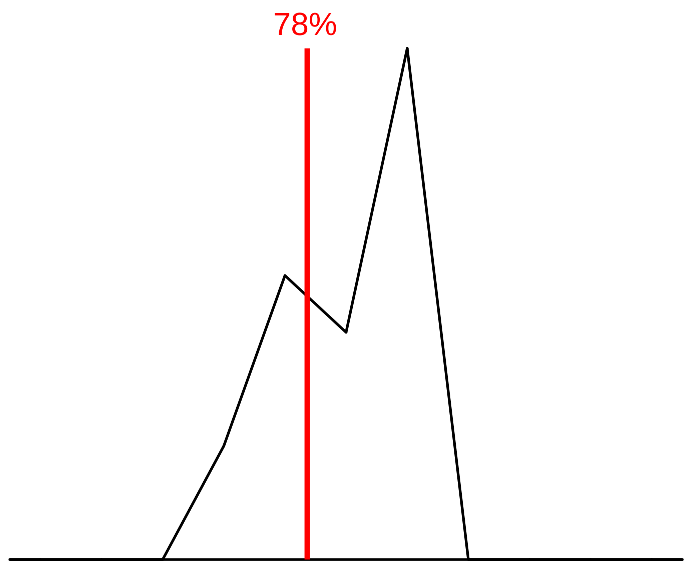

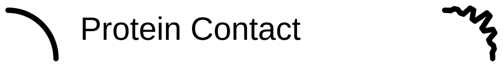

Residue Fingerprint

ALA256G	ALA278E
ALA278F	ALA293B
ALA293C	ALA336B
ARG252G	ARG291B
ASP316F	ASP333B
GLN255G	GLU264G
GLU292B	GLU292C
GLY290B	ILE263G
LYS260G	THR259G
VAL279E	VAL279F

Acceptor Donor
Metal Contact

Shape -15.27

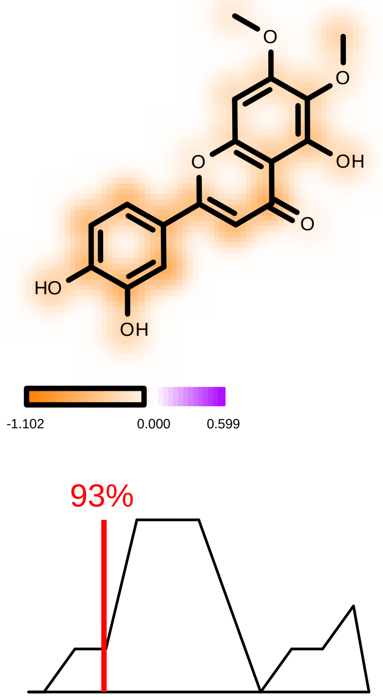

Hydrogen Bond -1.86

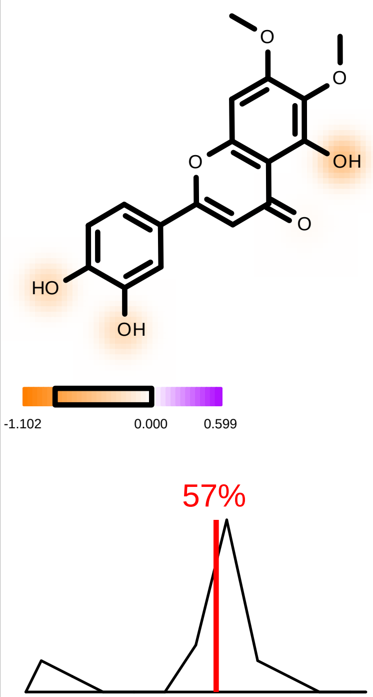

Protein Desolvation 3.65

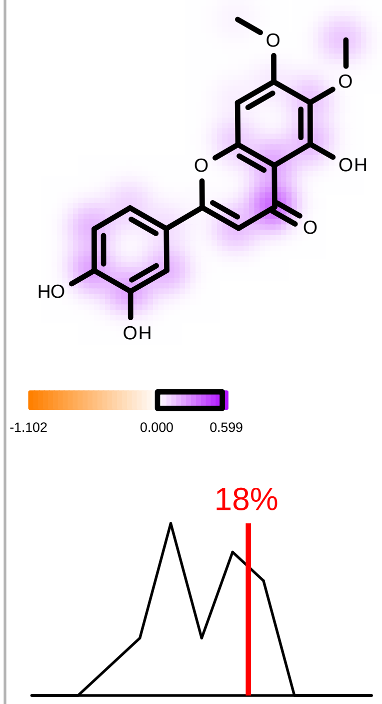

Ligand Desolvation 2.69

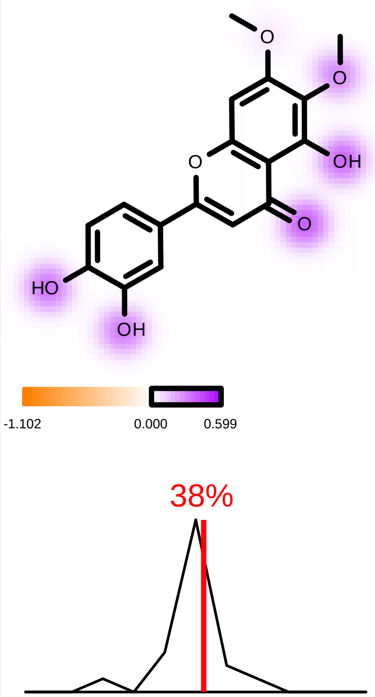

Molecule Name	omega_1_12
Molecular Weight	330.3
XLogP	1.2
PSA	109.4
Heavy Atoms	24
Acceptor Count	7
Donor Count	3
Chelator Count	3
Pose Index	6

Total Score -10.75

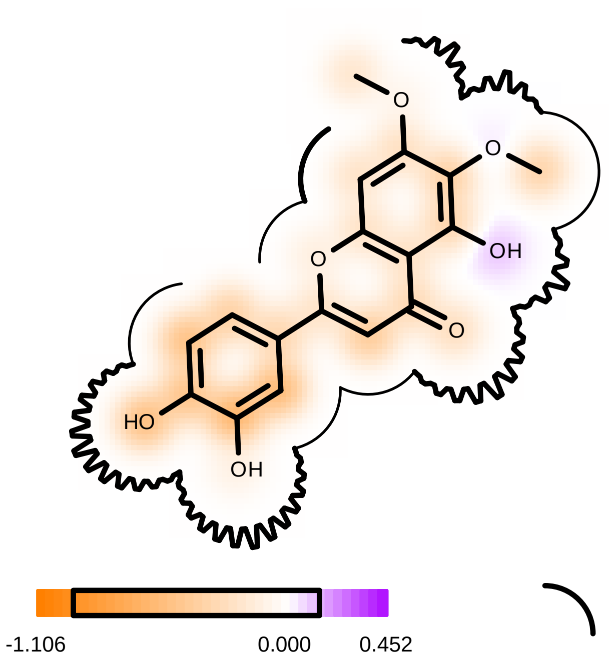

Score compared to other molecules

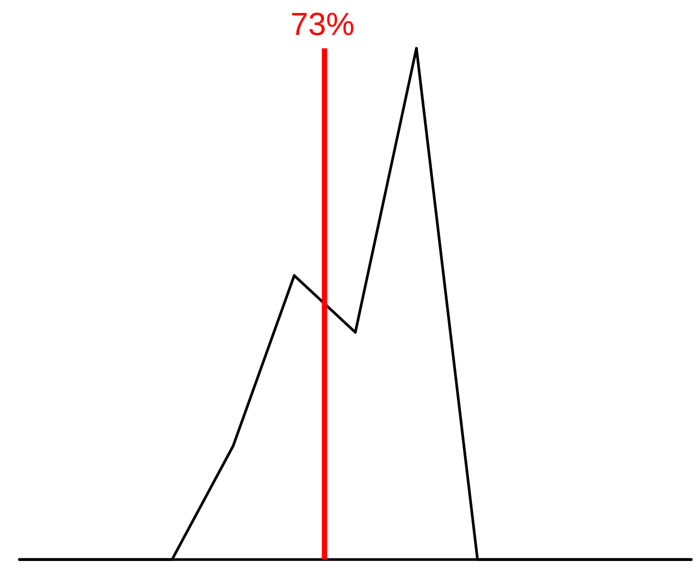

Residue Fingerprint

ALA256G	ALA278E
ALA278F	ALA293B
ALA293C	ALA336B
ARG252G	ARG291B
ASP316F	ASP333B
GLN255G	GLU264G
GLU292B	GLU292C
GLY290B	ILE263G
LYS260G	THR259G
VAL279E	VAL279F

Acceptor Donor
Metal Contact

Shape -14.16

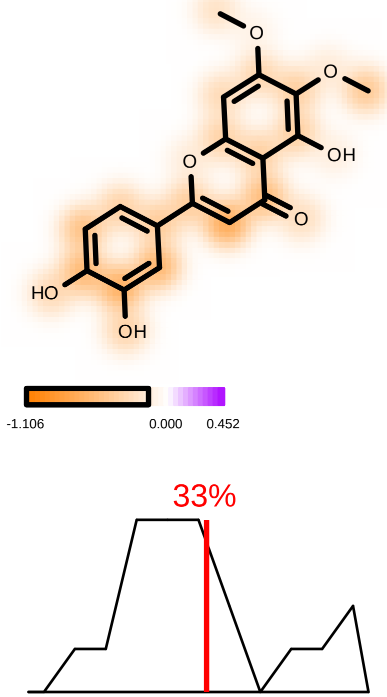

Hydrogen Bond -1.56

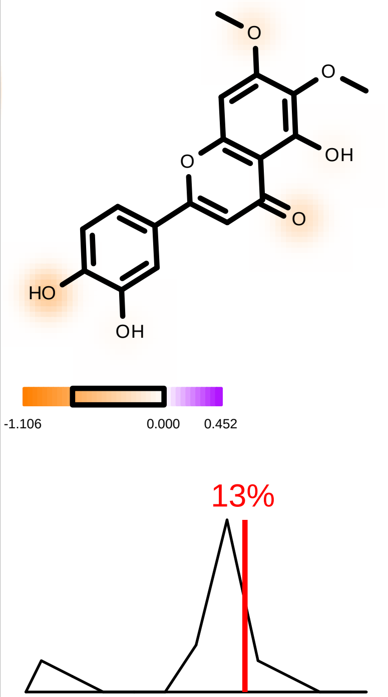

Protein Desolvation 2.62

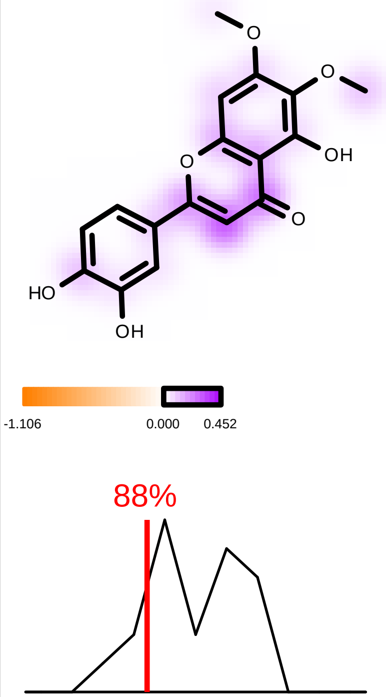

Ligand Desolvation 2.34

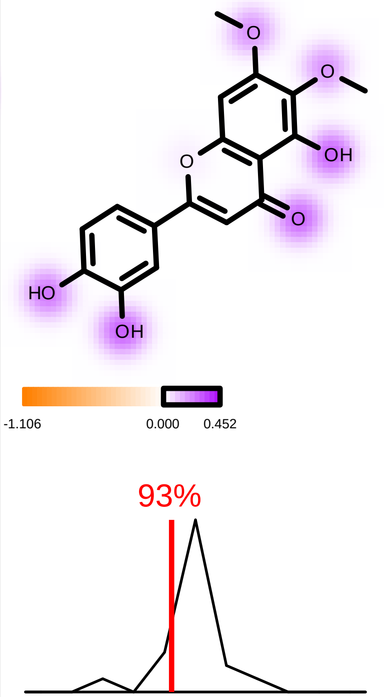

Molecule Name	omega_1_12
Molecular Weight	330.3
XLogP	1.2
PSA	109.4
Heavy Atoms	24
Acceptor Count	7
Donor Count	3
Chelator Count	3
Pose Index	7

Total Score -10.75

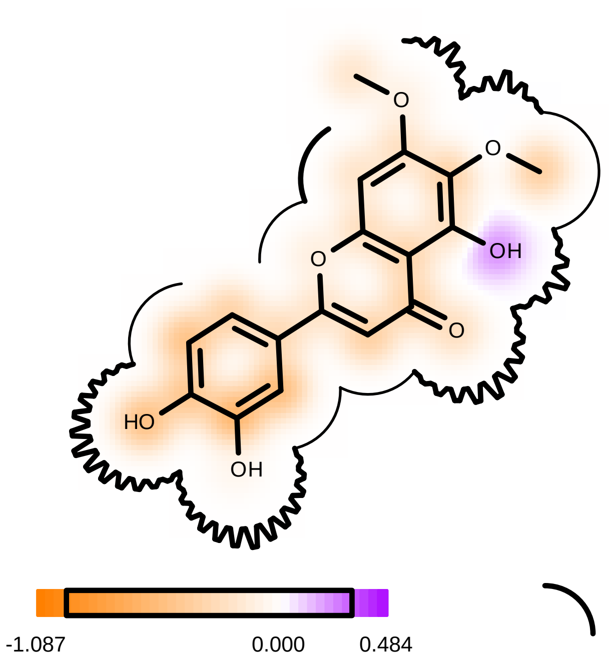

Score compared to other molecules

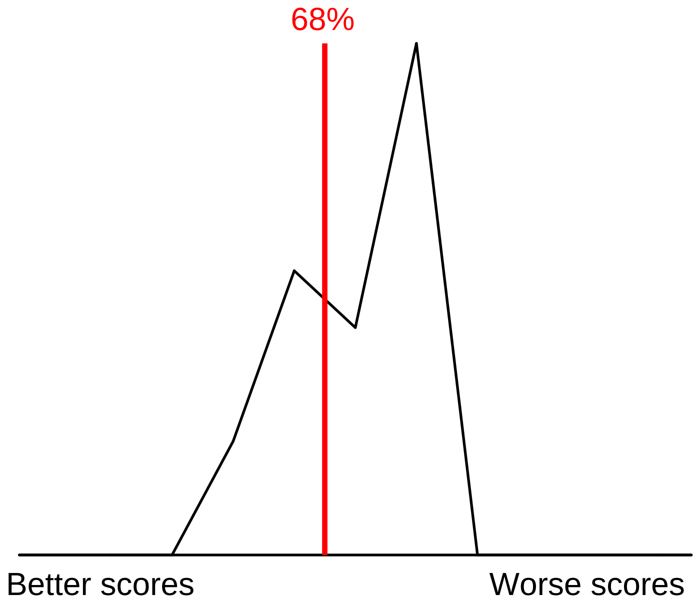

Residue Fingerprint

ALA256G	ALA278E
ALA278F	ALA293B
ALA293C	ALA336B
ARG252G	ARG291B
ASP316F	ASP333B
GLN255G	GLU264G
GLU292B	GLU292C
GLY290B	ILE263G
LYS260G	THR259G
VAL279E	VAL279F

Acceptor Donor
Metal Contact

Shape -14.38

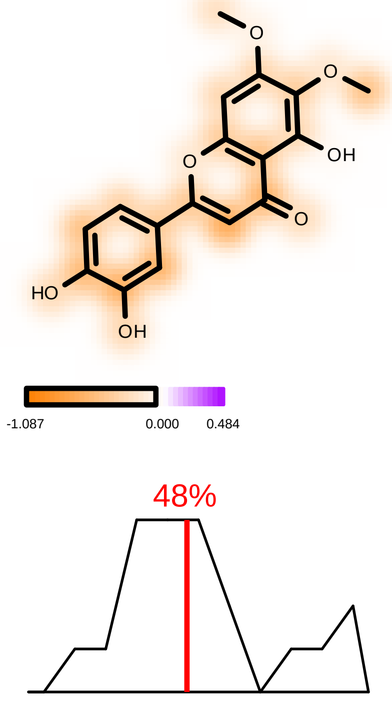

Hydrogen Bond -1.60

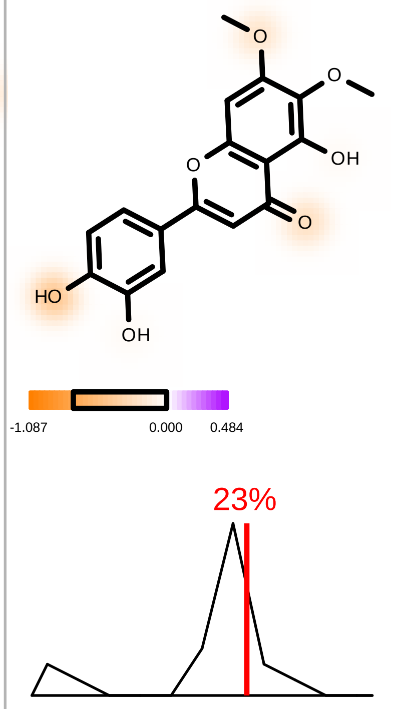

Protein Desolvation 2.82

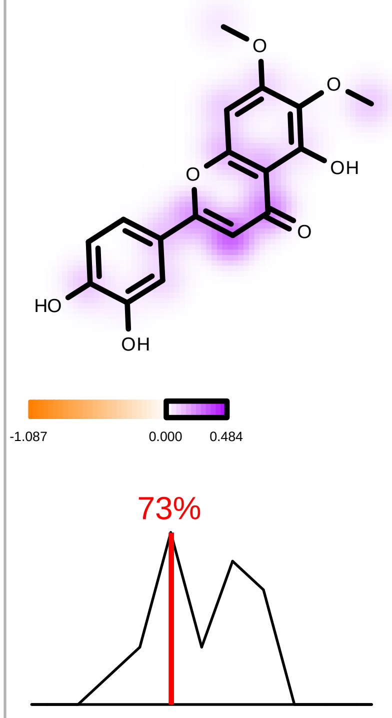

Ligand Desolvation 2.41

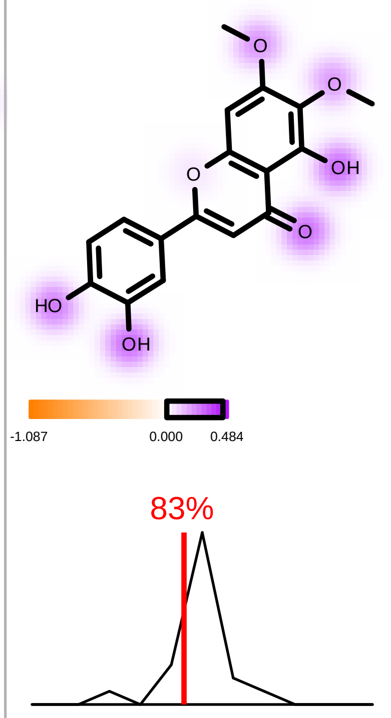

Molecule Name	omega_1_16
Molecular Weight	330.3
XLogP	1.2
PSA	109.4
Heavy Atoms	24
Acceptor Count	7
Donor Count	3
Chelator Count	3
Pose Index	8

Total Score -10.74

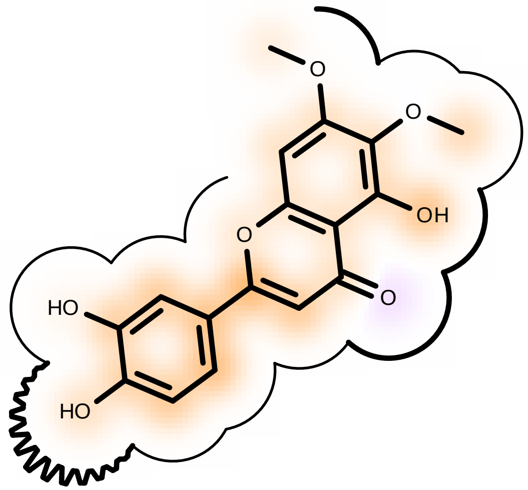

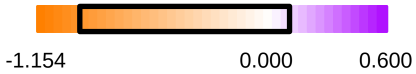

Score compared to other molecules

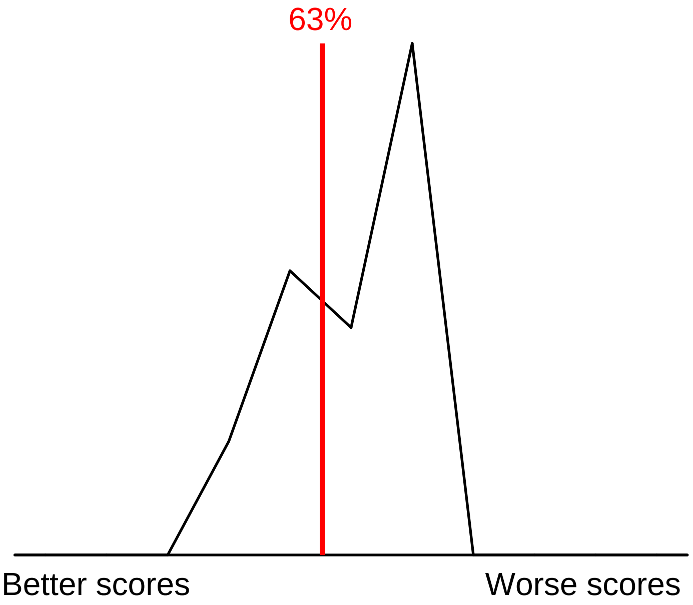

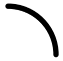 Protein Contact

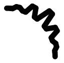 Protein Cavity

Residue Fingerprint

ALA256G	ALA278E
ALA278F	**ALA293B**
ALA293C	ALA336B
ARG252G	ARG291B
ASP316F	ASP333B
GLN255G	GLU264G
**GLU292B**	GLU292C
GLY290B	ILE263G
LYS260G	THR259G
VAL279E	VAL279F

Acceptor	Donor
Metal	Contact

Shape -15.55

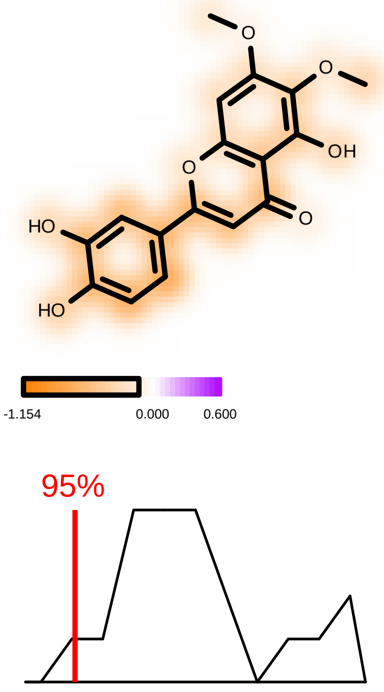

Hydrogen Bond -1.65

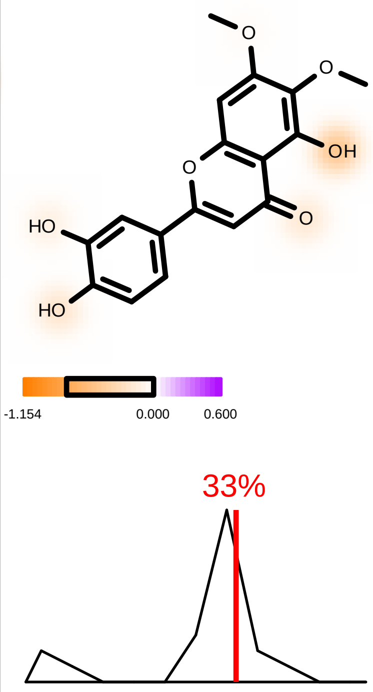

Protein Desolvation 3.74

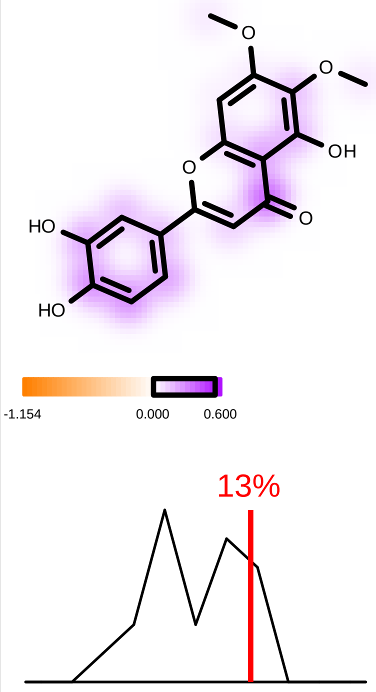

Ligand Desolvation 2.72

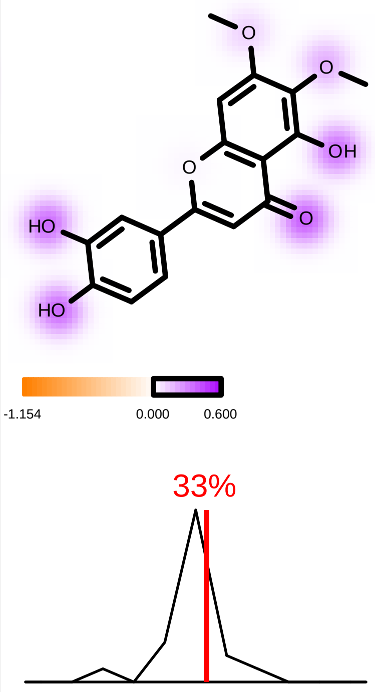

Molecule Name	omega_1_16
Molecular Weight	330.3
XLogP	1.2
PSA	109.4
Heavy Atoms	24
Acceptor Count	7
Donor Count	3
Chelator Count	3
Pose Index	9

Total Score -10.70

Score compared to other molecules

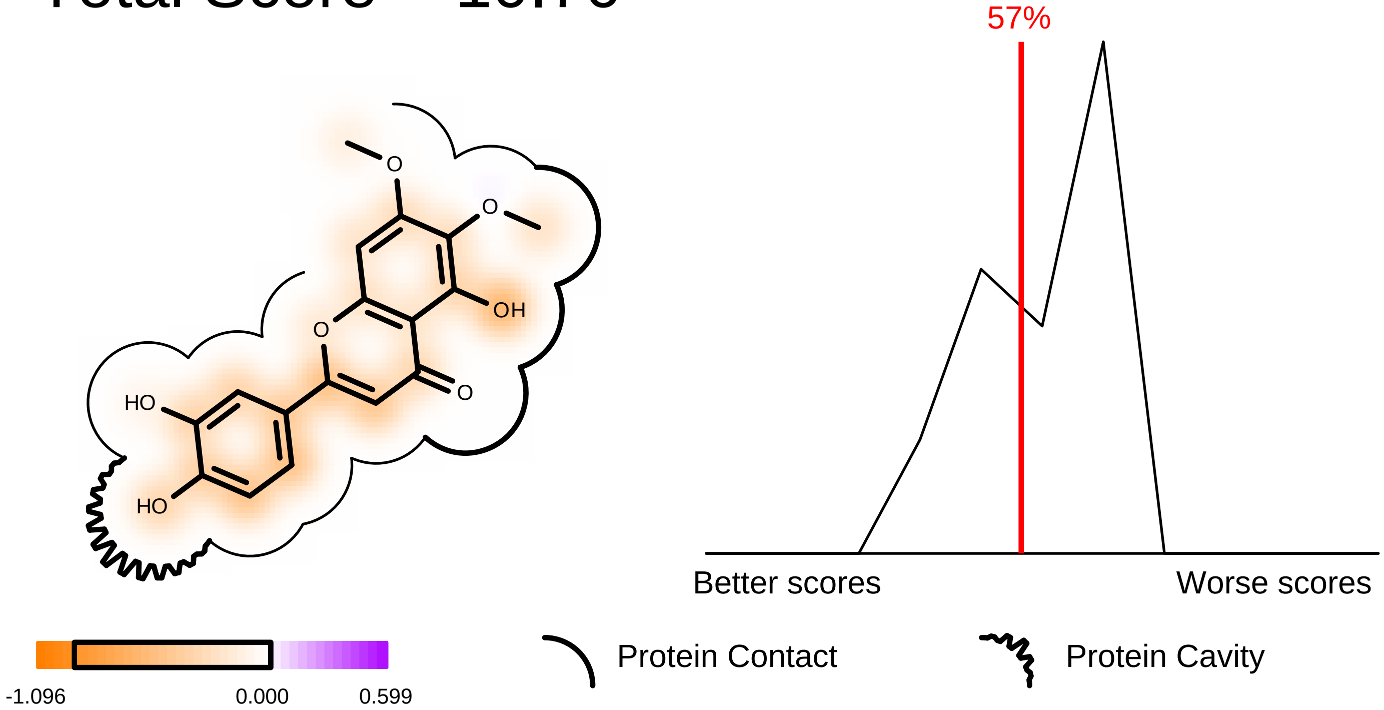

Residue Fingerprint

ALA256G	ALA278E
ALA278F	ALA293B
ALA293C	ALA336B
ARG252G	ARG291B
ASP316F	ASP333B
GLN255G	GLU264G
GLU292B	GLU292C
GLY290B	ILE263G
LYS260G	THR259G
VAL279E	VAL279F

Acceptor Donor
Metal Contact

Shape -14.83

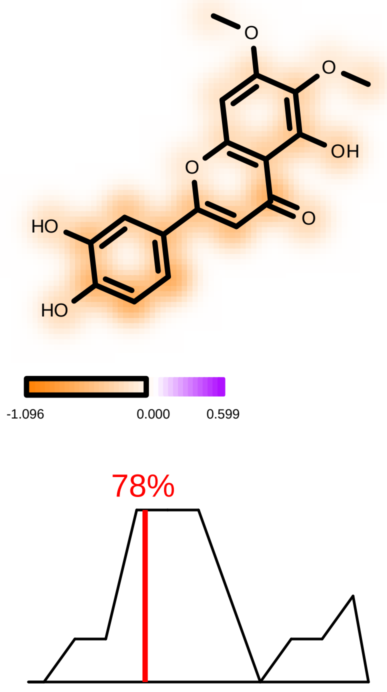

Hydrogen Bond -1.82

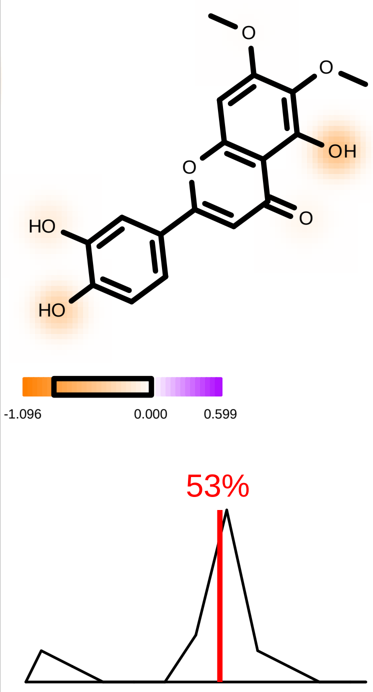

Protein Desolvation 3.35

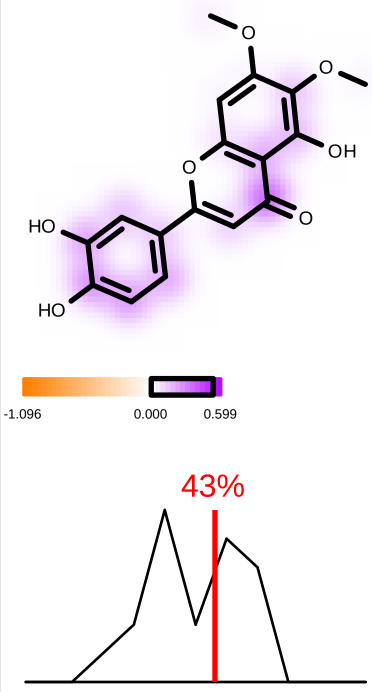

Ligand Desolvation 2.60

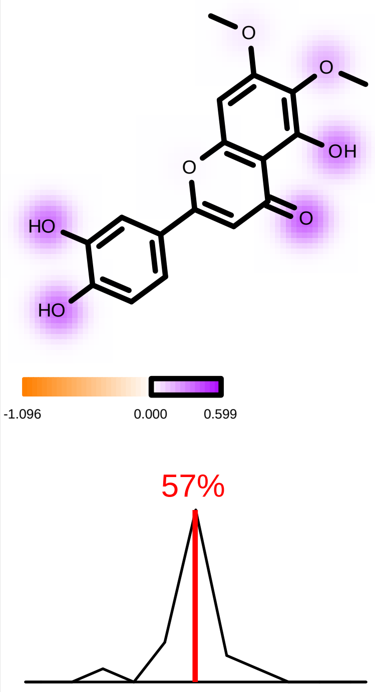

Molecule Name	omega_1_14
Molecular Weight	330.3
XLogP	1.2
PSA	109.4
Heavy Atoms	24
Acceptor Count	7
Donor Count	3
Chelator Count	3
Pose Index	10

Total Score -10.64

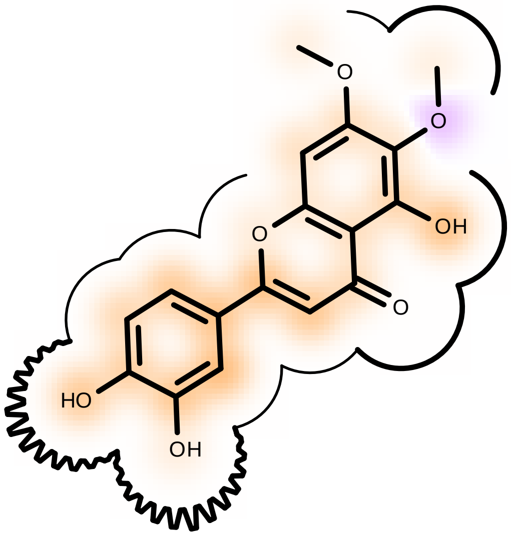

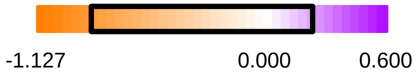

Score compared to other molecules

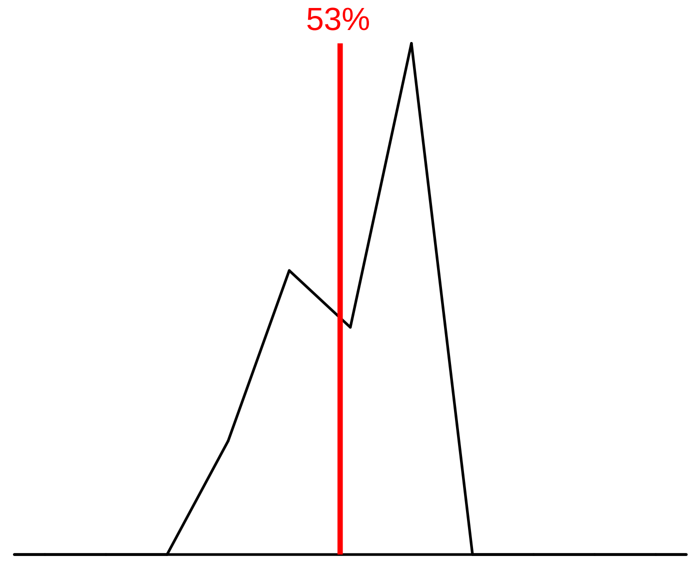

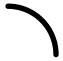 Protein Contact

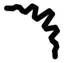 Protein Cavity

Residue Fingerprint

ALA256G	ALA278E
ALA278F	**ALA293B**
ALA293C	ALA336B
ARG252G	ARG291B
ASP316F	ASP333B
GLN255G	**GLU264G**
**GLU292B**	GLU292C
GLY290B	ILE263G
**LYS260G**	THR259G
VAL279E	VAL279F

Acceptor Donor
Metal Contact

Shape -14.85

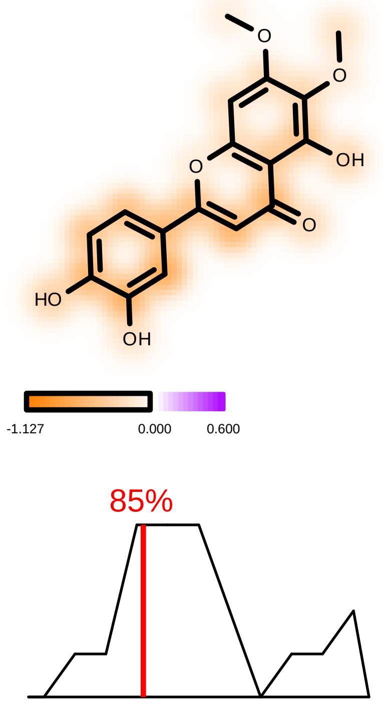

Hydrogen Bond -1.89

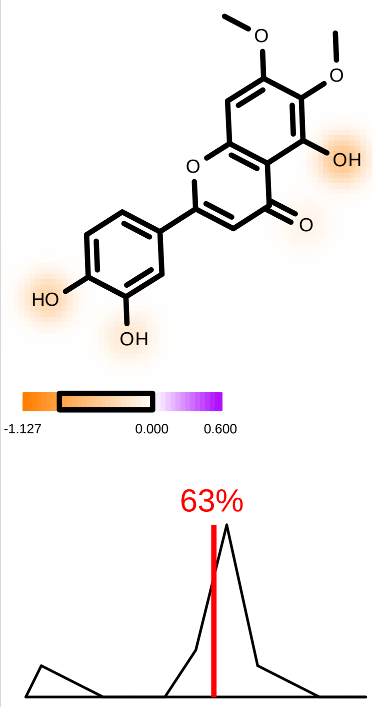

Protein Desolvation 3.48

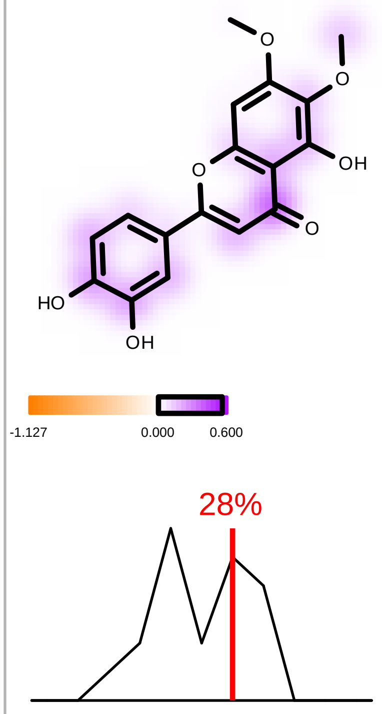

Ligand Desolvation 2.62

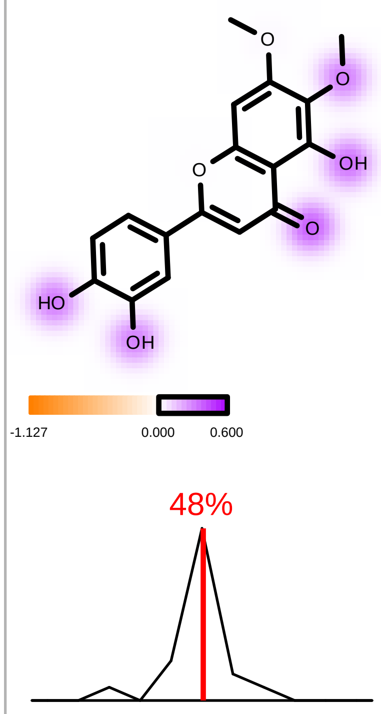

Molecule Name	omega_1_16
Molecular Weight	330.3
XLogP	1.2
PSA	109.4
Heavy Atoms	24
Acceptor Count	7
Donor Count	3
Chelator Count	3
Pose Index	11

Total Score -10.62

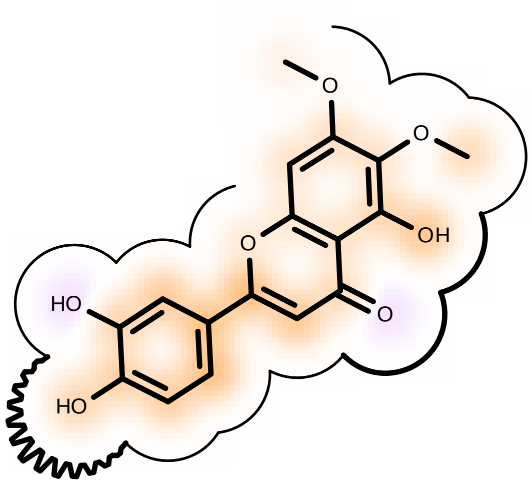

Score compared to other molecules

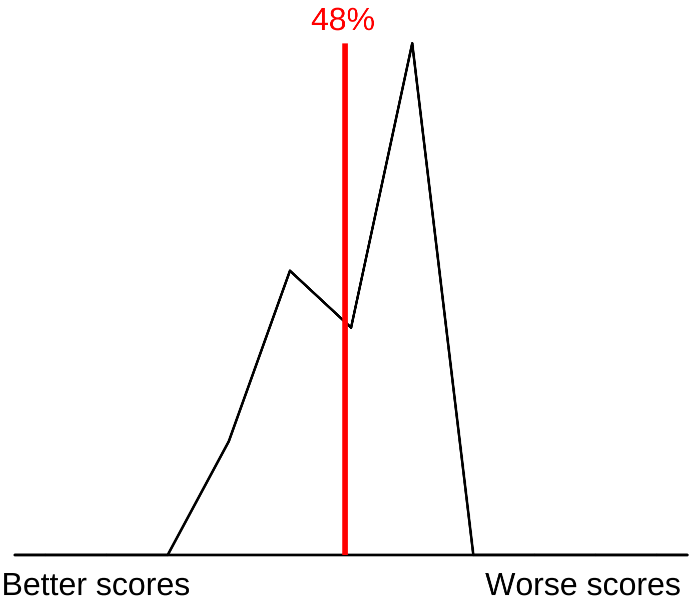

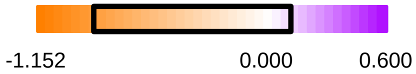

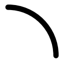 Protein Contact

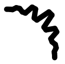 Protein Cavity

Residue Fingerprint

ALA256G	ALA278E
ALA278F	**ALA293B**
ALA293C	ALA336B
ARG252G	ARG291B
ASP316F	ASP333B
GLN255G	**GLU264G**
**GLU292B**	GLU292C
GLY290B	ILE263G
LYS260G	THR259G
VAL279E	VAL279F

Acceptor	Donor
Metal	Contact

Shape -15.04

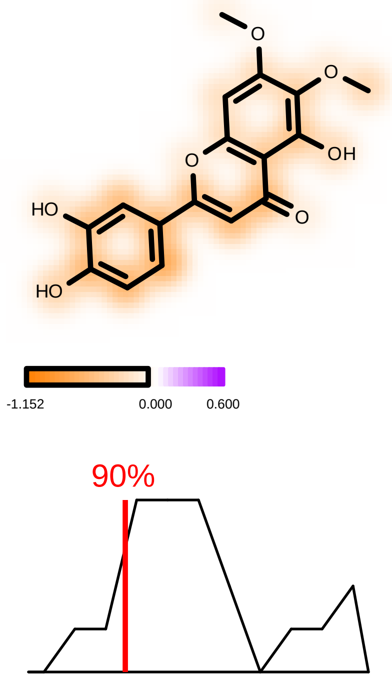

Hydrogen Bond -1.72

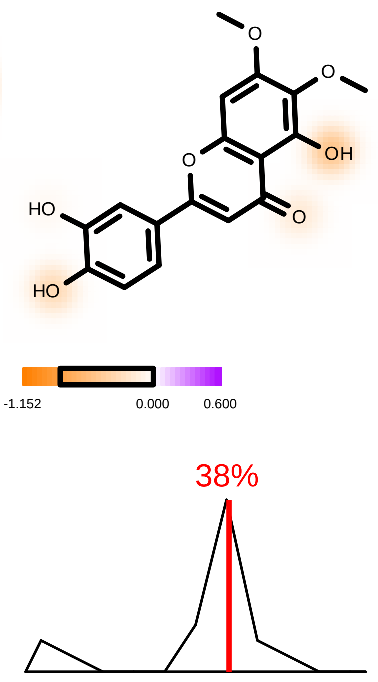

Protein Desolvation 3.59

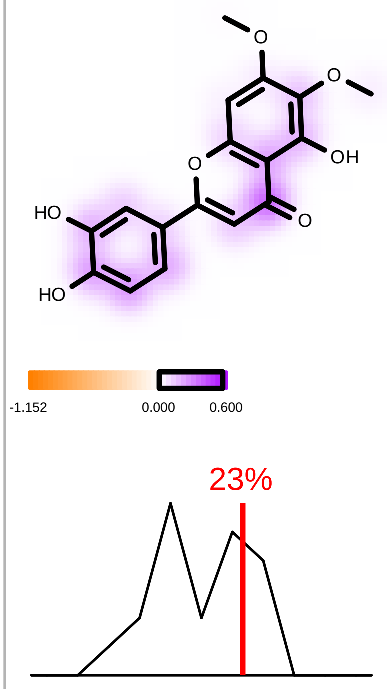

Ligand Desolvation 2.55

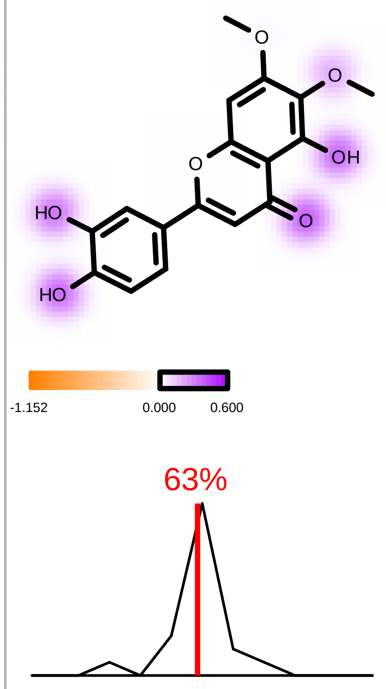

Molecule Name	omega_1_16
Molecular Weight	330.3
XLogP	1.2
PSA	109.4
Heavy Atoms	24
Acceptor Count	7
Donor Count	3
Chelator Count	3
Pose Index	12

Total Score -10.36

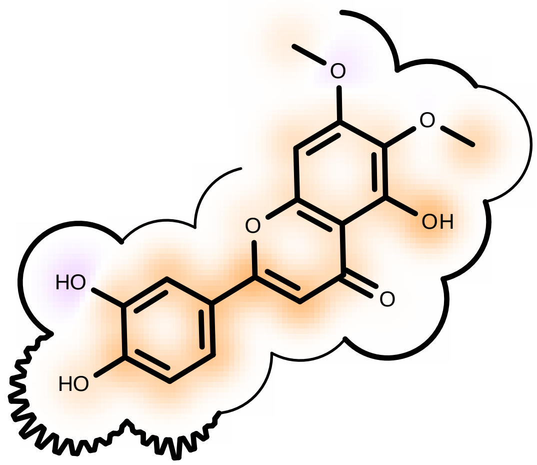

Score compared to other molecules

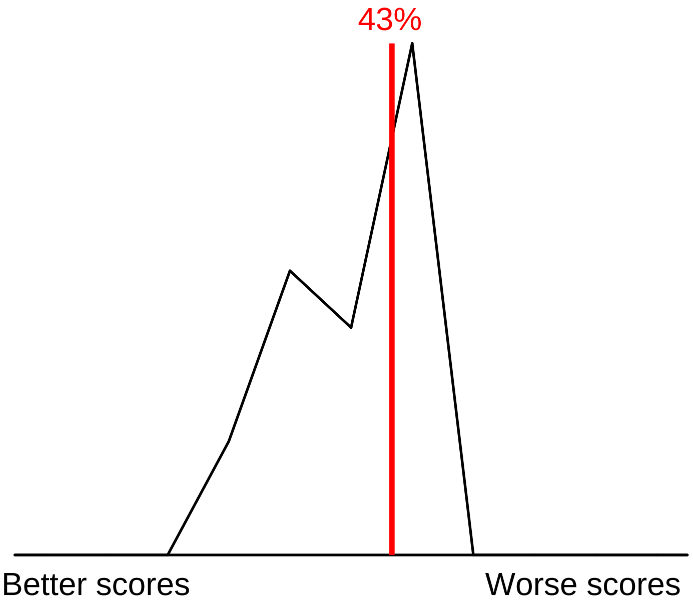

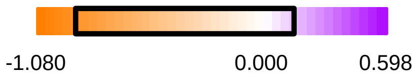

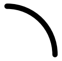 Protein Contact

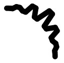 Protein Cavity

Residue Fingerprint

ALA256G	ALA278E
ALA278F	ALA293B
ALA293C	ALA336B
ARG252G	ARG291B
ASP316F	ASP333B
GLN255G	GLU264G
GLU292B	GLU292C
GLY290B	ILE263G
LYS260G	THR259G
VAL279E	VAL279F

Acceptor	Donor
Metal	Contact

Shape -14.82

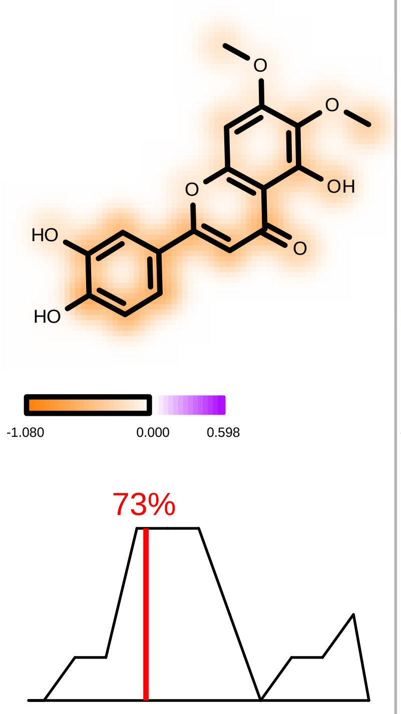

Hydrogen Bond -1.89

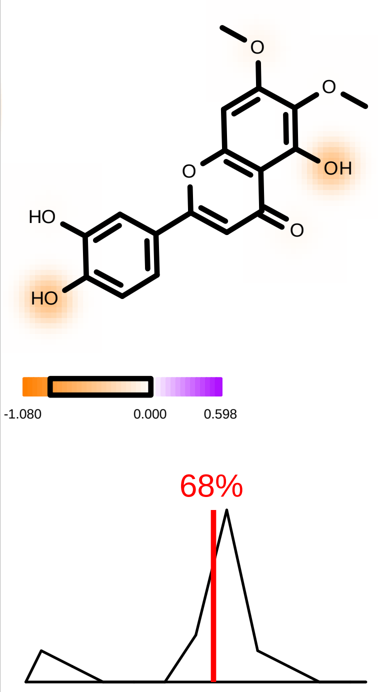

Protein Desolvation 3.38

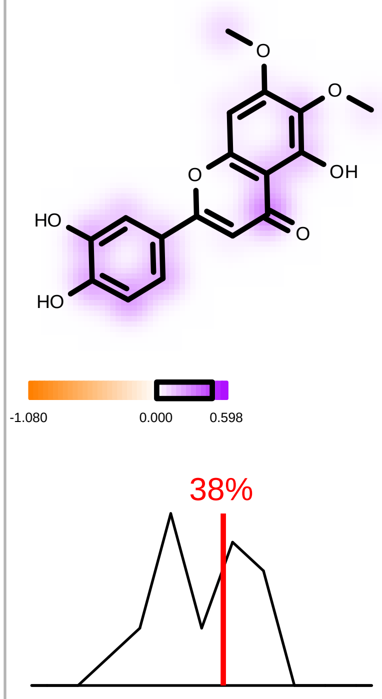

Ligand Desolvation 2.97

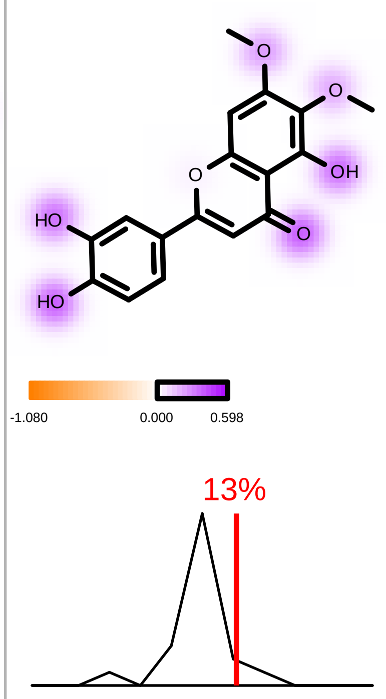

Molecule Name	omega_1_2
Molecular Weight	330.3
XLogP	1.2
PSA	109.4
Heavy Atoms	24
Acceptor Count	7
Donor Count	3
Chelator Count	3
Pose Index	13

Total Score -10.35

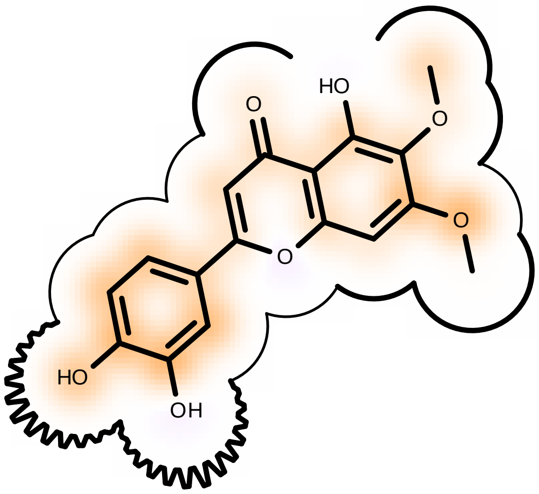

Score compared to other molecules

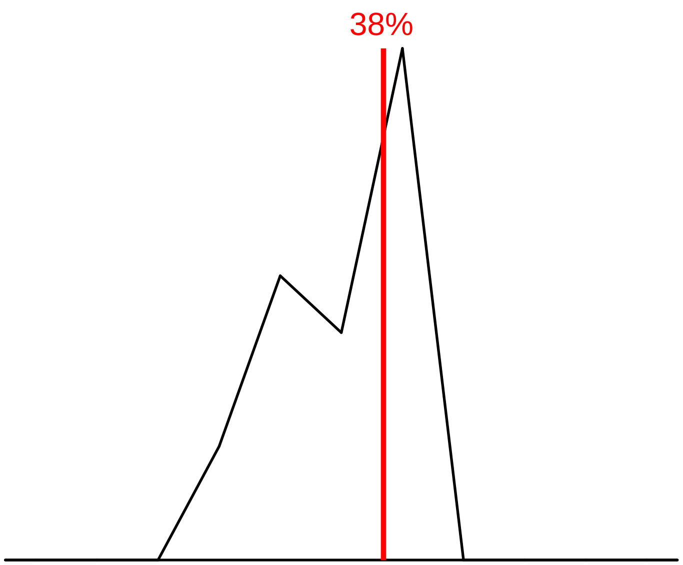

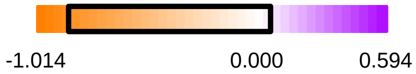

Protein Contact

Protein Cavity

Residue Fingerprint

ALA256G	ALA278E
ALA278F	ALA293B
ALA293C	ALA336B
ARG252G	ARG291B
ASP316F	ASP333B
GLN255G	GLU264G
GLU292B	GLU292C
GLY290B	ILE263G
LYS260G	**THR259G**
VAL279E	**VAL279F**

Acceptor	Donor
Metal	Contact

Shape -14.17

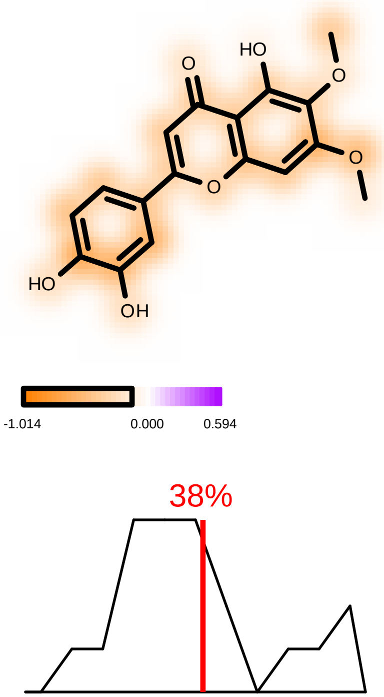

Hydrogen Bond -1.61

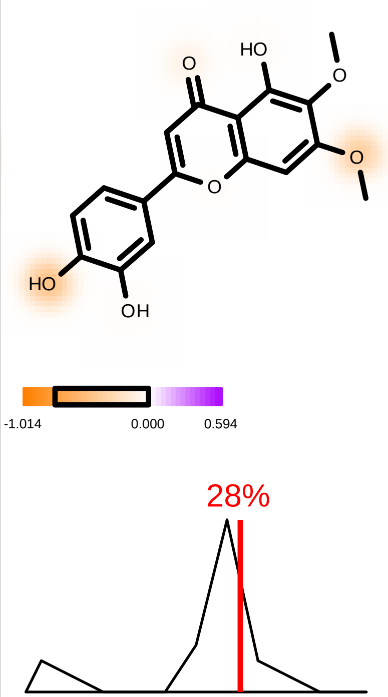

Protein Desolvation 2.78

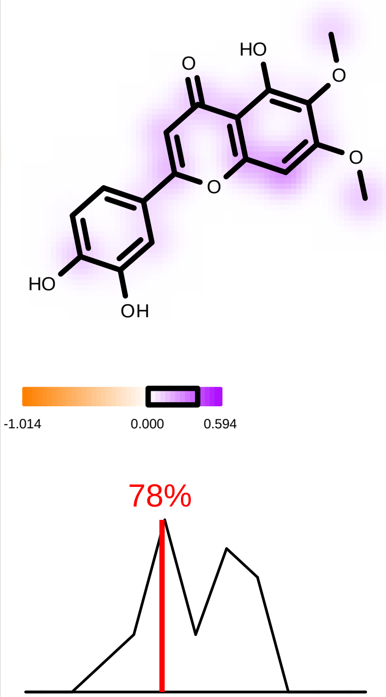

Ligand Desolvation 2.64

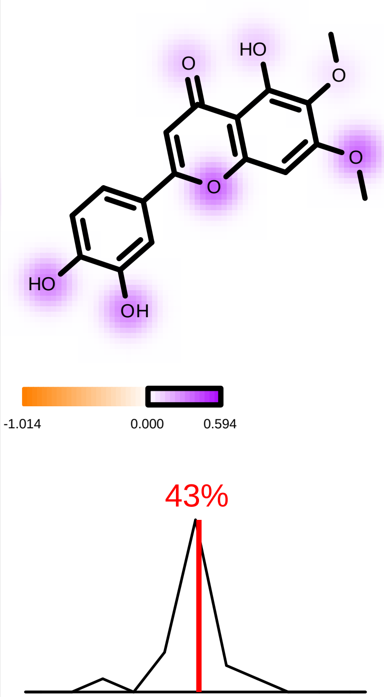

Molecule Name	omega_1_16
Molecular Weight	330.3
XLogP	1.2
PSA	109.4
Heavy Atoms	24
Acceptor Count	7
Donor Count	3
Chelator Count	3
Pose Index	14

Total Score -10.32

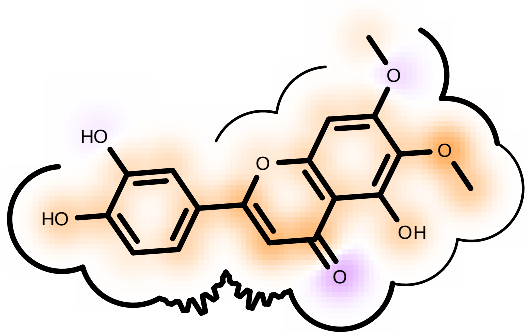

Score compared to other molecules

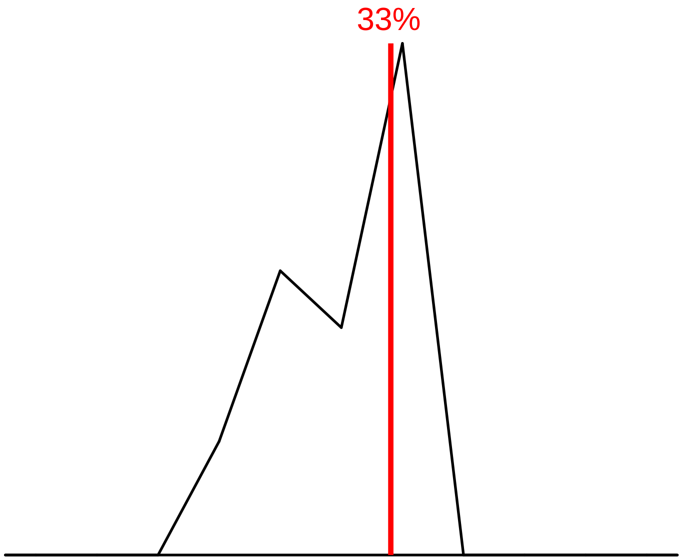

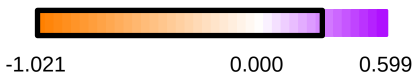

Protein Contact

Protein Cavity

Residue Fingerprint

ALA256G	ALA278E
ALA278F	ALA293B
ALA293C	ALA336B
ARG252G	ARG291B
ASP316F	ASP333B
GLN255G	GLU264G
**GLU292B**	**GLU292C**
**GLY290B**	ILE263G
LYS260G	THR259G
VAL279E	VAL279F

Acceptor	Donor
Metal	Contact

Shape -12.78

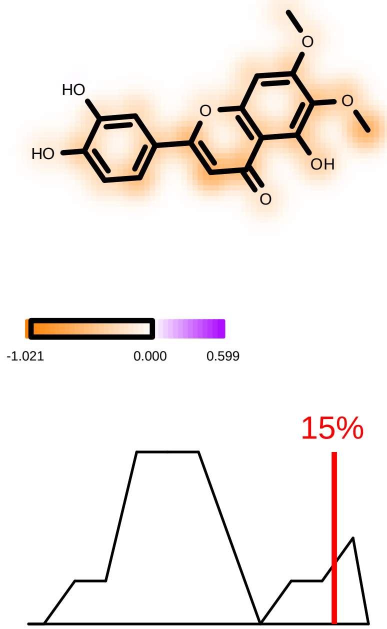

Hydrogen Bond -2.07

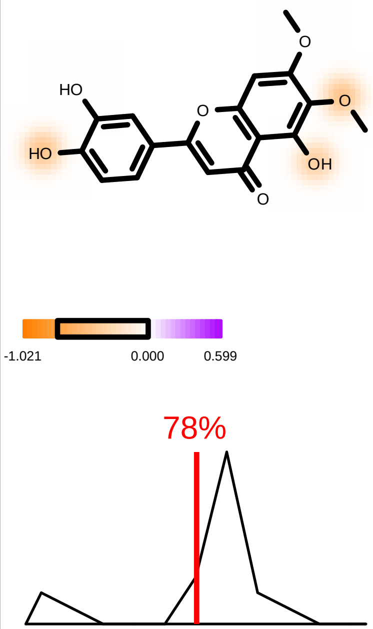

Protein Desolvation 2.18

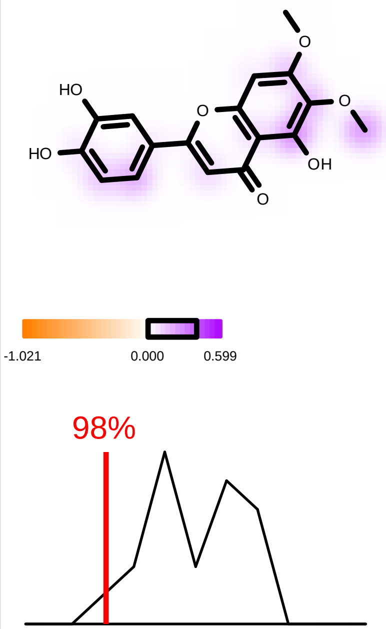

Ligand Desolvation 2.37

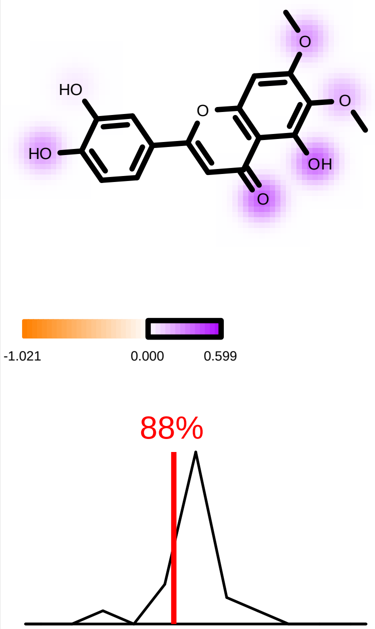

Molecule Name	omega_1_13
Molecular Weight	330.3
XLogP	1.2
PSA	109.4
Heavy Atoms	24
Acceptor Count	7
Donor Count	3
Chelator Count	3
Pose Index	15

Total Score -10.30

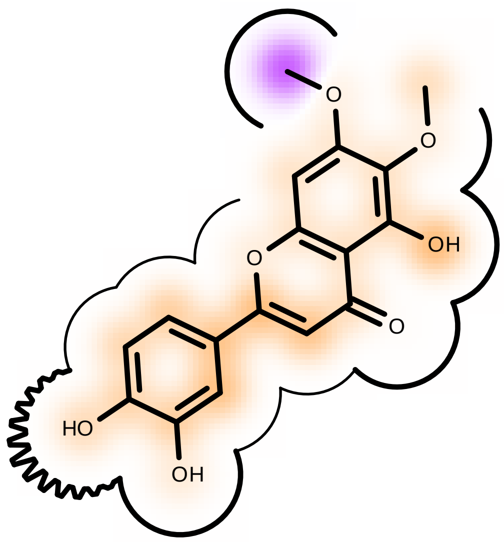

Score compared to other molecules

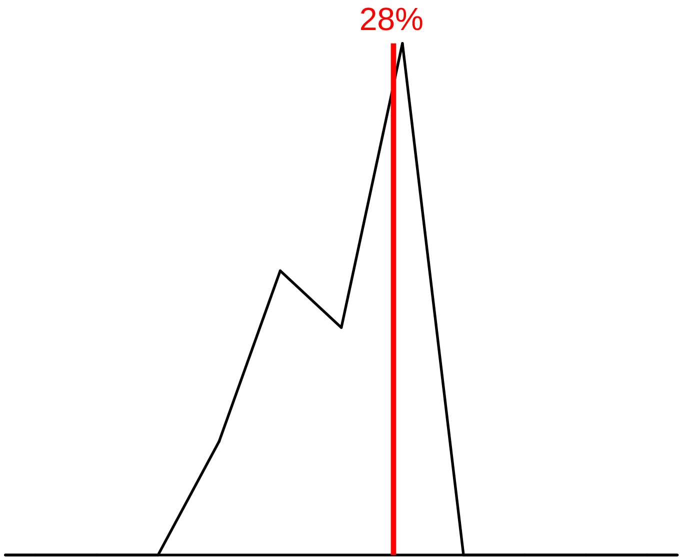

Better scores Worse scores

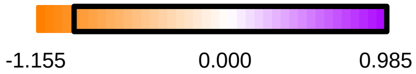

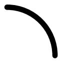 Protein Contact 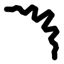 Protein Cavity

Residue Fingerprint

ALA256G	ALA278E
ALA278F	ALA293B
ALA293C	ALA336B
ARG252G	ARG291B
ASP316F	ASP333B
GLN255G	**GLU264G**
**GLU292B**	GLU292C
GLY290B	ILE263G
**LYS260G**	THR259G
VAL279E	VAL279F

Shape -14.74

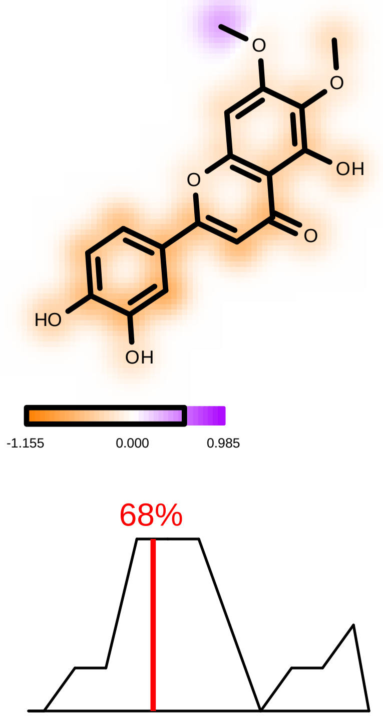

Hydrogen Bond -2.13

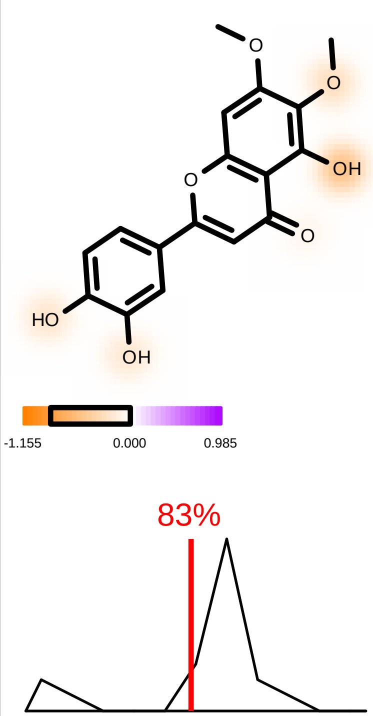

Protein Desolvation 3.86

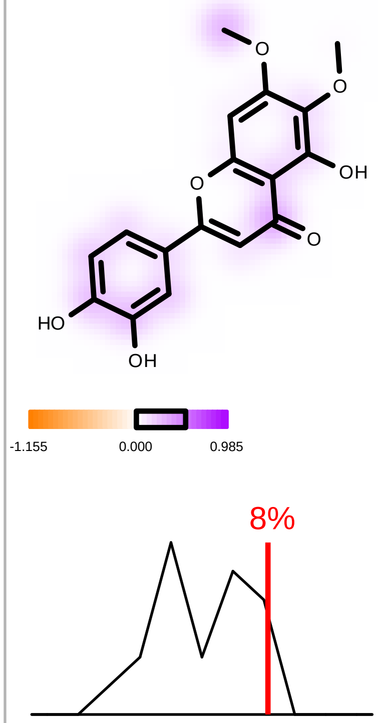

Ligand Desolvation 2.72

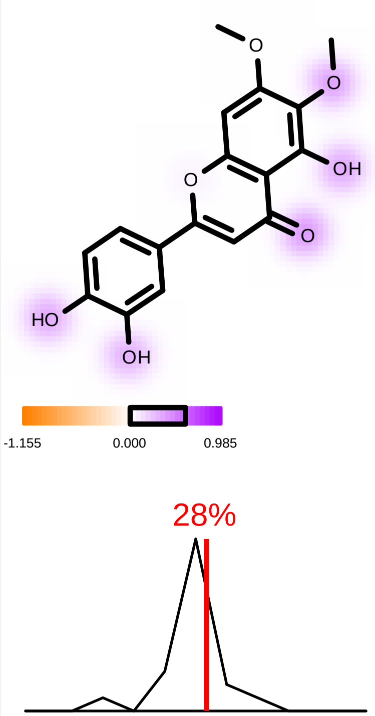

Acceptor	Donor
Metal	Contact

Molecule Name	omega_1_10
Molecular Weight	330.3
XLogP	1.2
PSA	109.4
Heavy Atoms	24
Acceptor Count	7
Donor Count	3
Chelator Count	3
Pose Index	16

Total Score -10.24

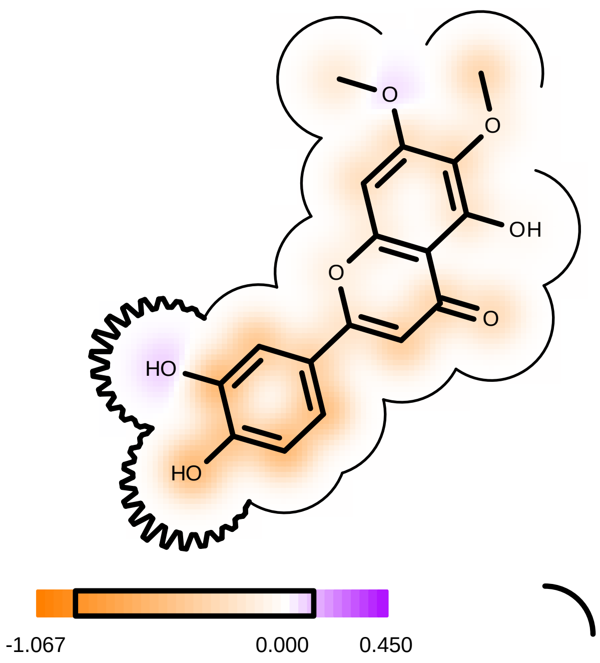

Score compared to other molecules

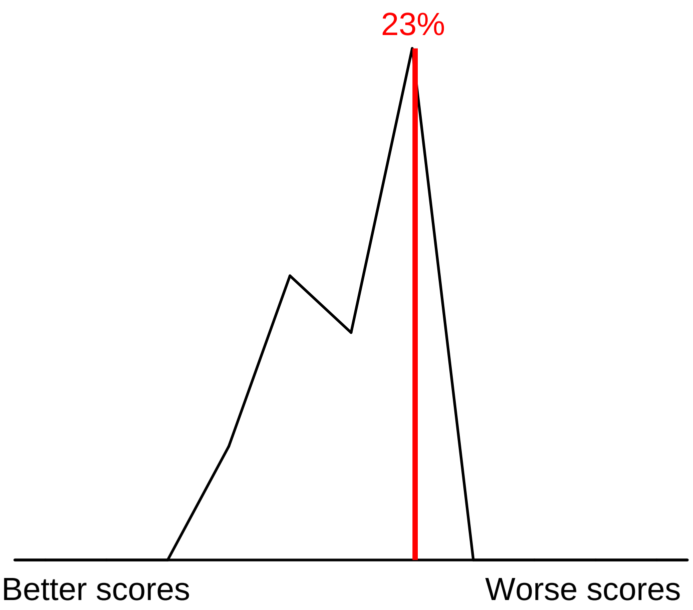

Protein Contact

Protein Cavity

Residue Fingerprint

ALA256G	ALA278E
ALA278F	ALA293B
ALA293C	ALA336B
ARG252G	ARG291B
ASP316F	ASP333B
GLN255G	GLU264G
GLU292B	GLU292C
GLY290B	ILE263G
LYS260G	THR259G
VAL279E	**VAL279F**

Acceptor	Donor
Metal	Contact

Shape -13.29

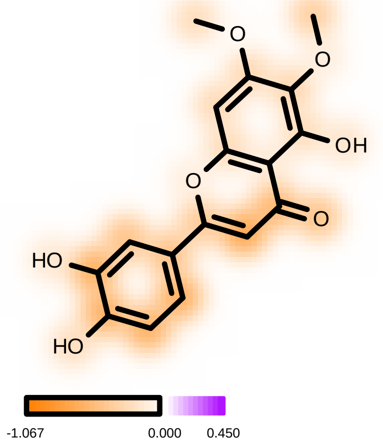

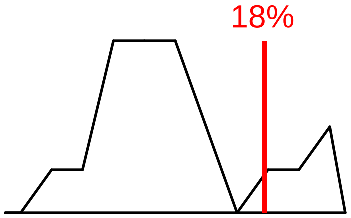

Hydrogen Bond -1.18

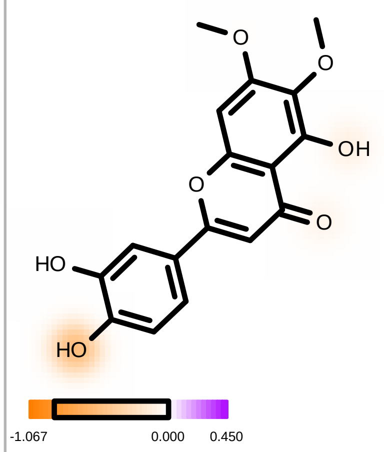

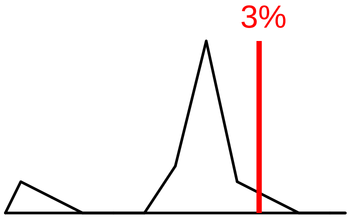

Protein Desolvation 2.47

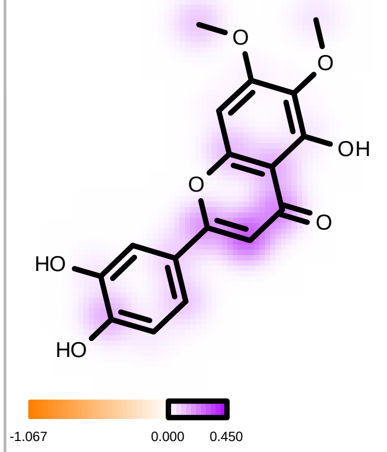

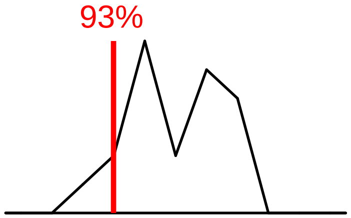

Ligand Desolvation 1.75

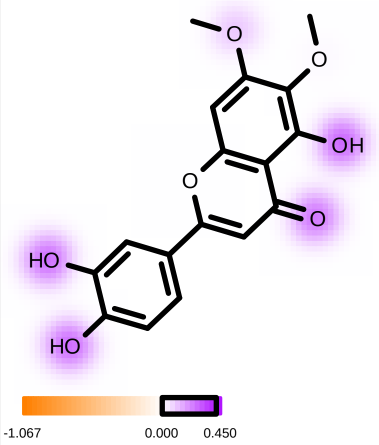

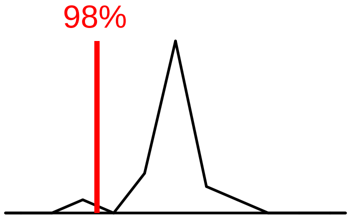

Molecule Name	omega_1_4
Molecular Weight	330.3
XLogP	1.2
PSA	109.4
Heavy Atoms	24
Acceptor Count	7
Donor Count	3
Chelator Count	3
Pose Index	17

Total Score -10.23

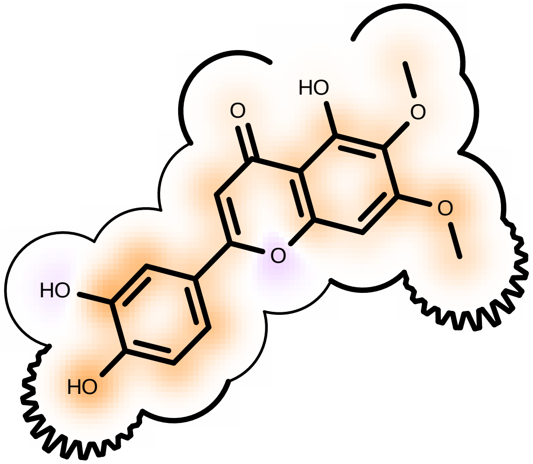

Score compared to other molecules

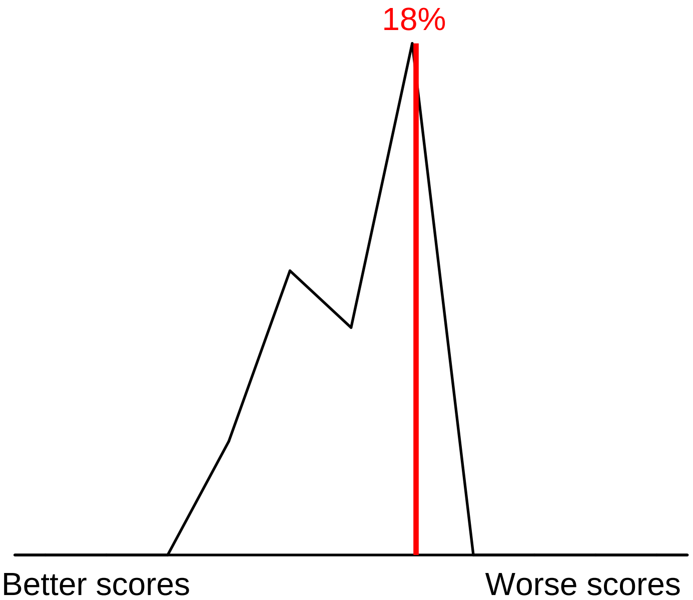

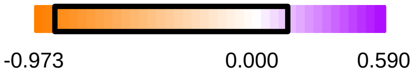

Protein Contact

Protein Cavity

Residue Fingerprint

ALA256G	ALA278E
ALA278F	ALA293B
ALA293C	ALA336B
ARG252G	ARG291B
ASP316F	ASP333B
GLN255G	GLU264G
GLU292B	GLU292C
GLY290B	ILE263G
LYS260G	**THR259G**
VAL279E	**VAL279F**

Shape -13.95

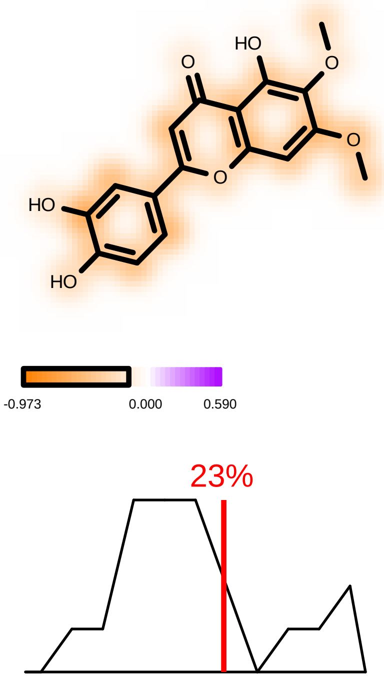

Hydrogen Bond -1.93

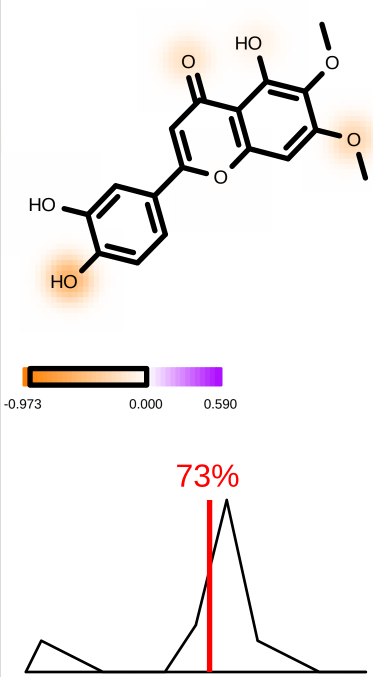

Protein Desolvation 3.04

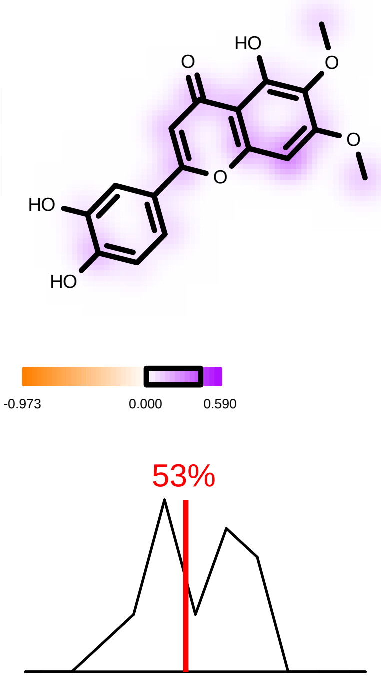

Ligand Desolvation 2.61

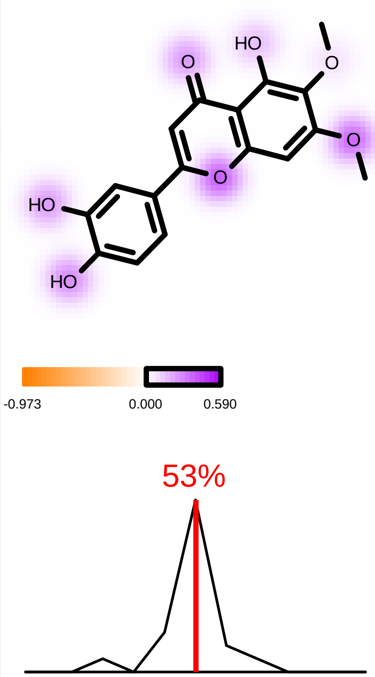

Acceptor Metal **Donor** Contact

Molecule Name	omega_1_14
Molecular Weight	330.3
XLogP	1.2
PSA	109.4
Heavy Atoms	24
Acceptor Count	7
Donor Count	3
Chelator Count	3
Pose Index	18

Total Score -10.22

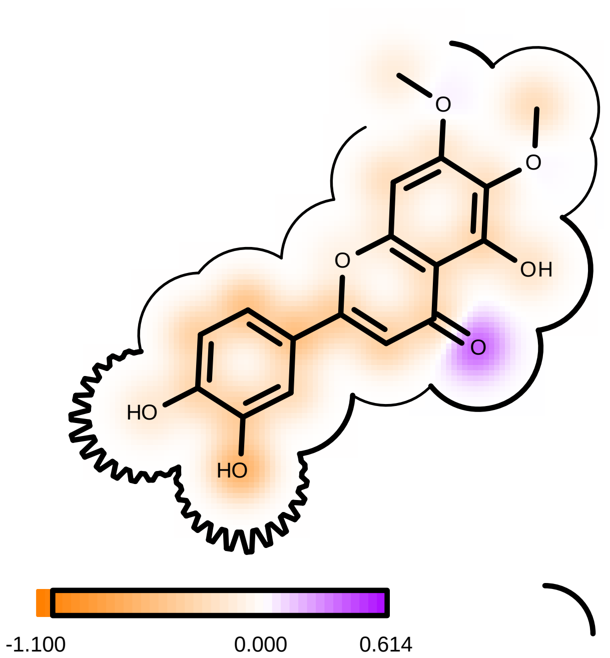

Score compared to other molecules

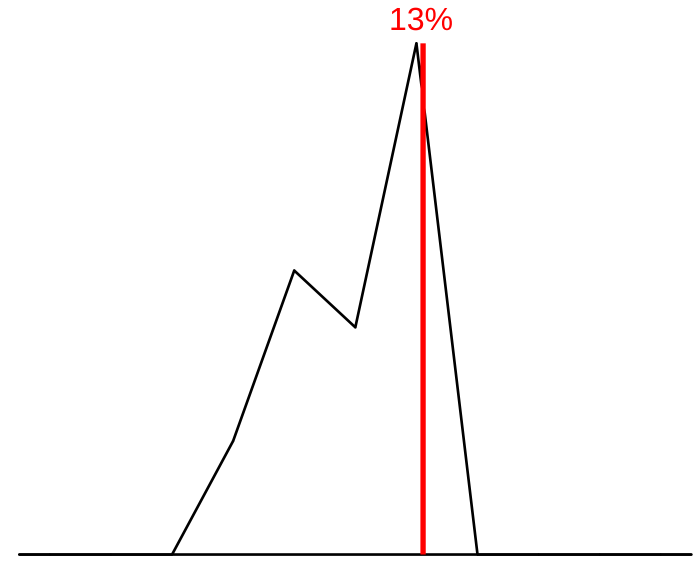

Protein Contact

Protein Cavity

Residue Fingerprint

ALA256G	ALA278E
ALA278F	ALA293B
ALA293C	ALA336B
ARG252G	ARG291B
ASP316F	ASP333B
GLN255G	GLU264G
**GLU292B**	GLU292C
GLY290B	ILE263G
**LYS260G**	THR259G
VAL279E	VAL279F

Acceptor Donor
Metal Contact

Shape -14.66

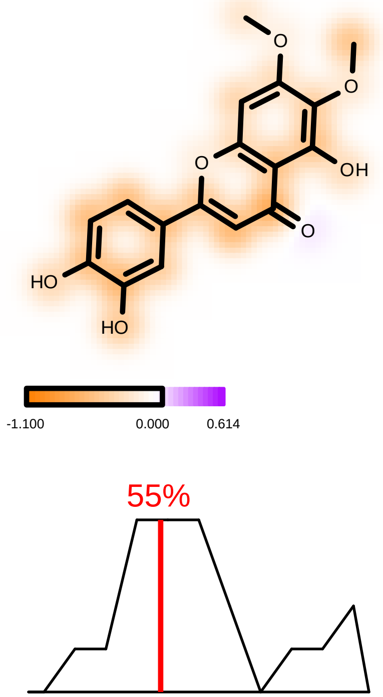

Hydrogen Bond -1.73

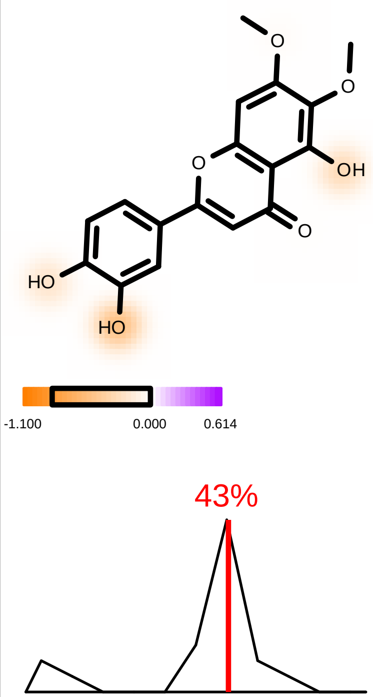

Protein Desolvation 3.43

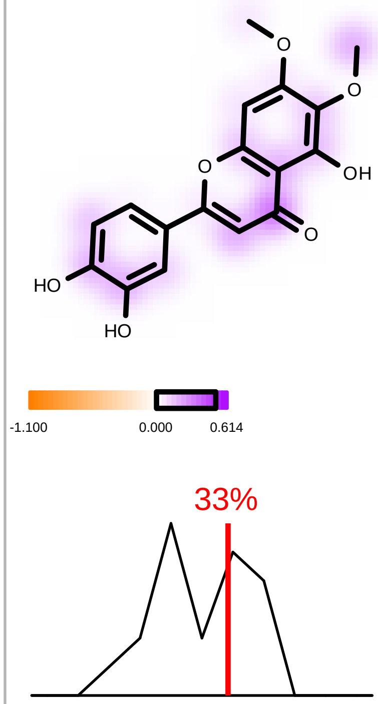

Ligand Desolvation 2.75

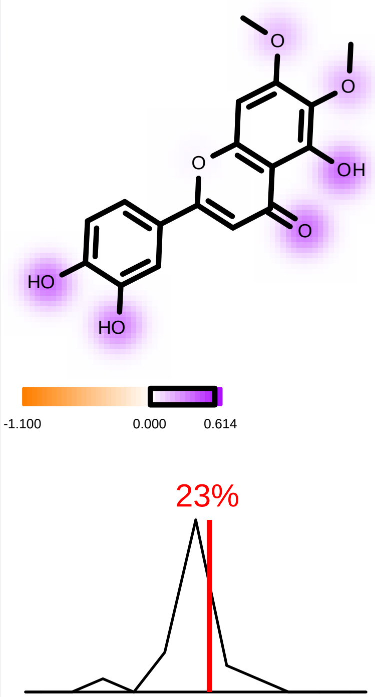

Molecule Name	omega_1_12
Molecular Weight	330.3
XLogP	1.2
PSA	109.4
Heavy Atoms	24
Acceptor Count	7
Donor Count	3
Chelator Count	3
Pose Index	19

Total Score -10.20

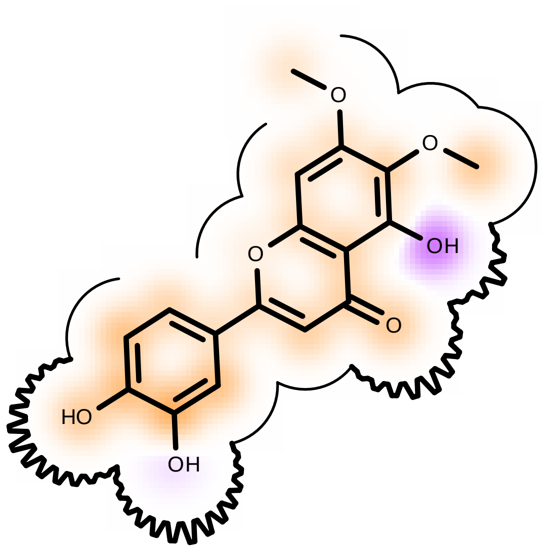

-1.045 0.000 0.482

Score compared to other molecules

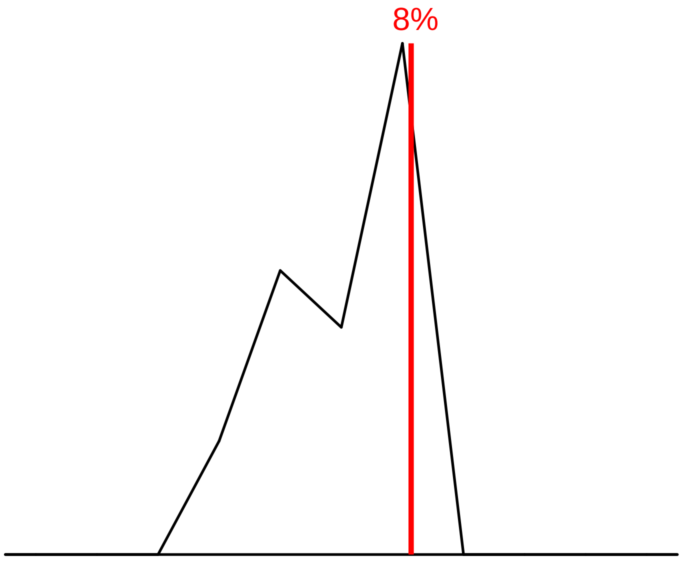

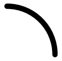 Protein Contact

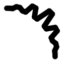 Protein Cavity

Residue Fingerprint

ALA256G	ALA278E
ALA278F	ALA293B
ALA293C	ALA336B
ARG252G	ARG291B
ASP316F	ASP333B
GLN255G	GLU264G
GLU292B	GLU292C
GLY290B	ILE263G
LYS260G	**THR259G**
VAL279E	**VAL279F**

Acceptor **Donor**
Metal Contact

Shape -14.23

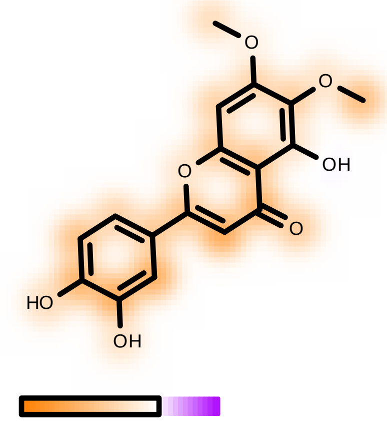

-1.045 0.000 0.482

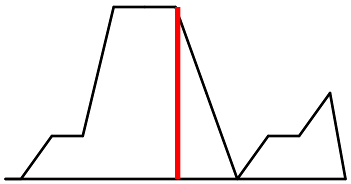

Hydrogen Bond -1.40

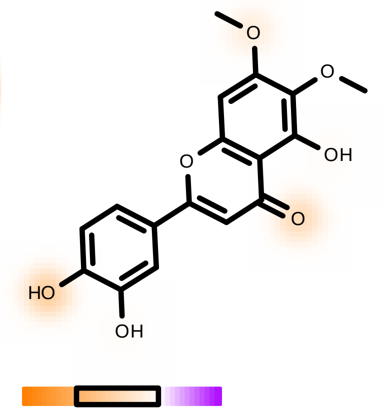

-1.045 0.000 0.482

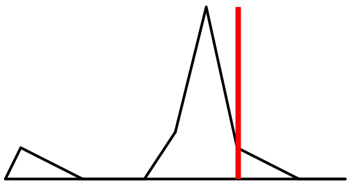

Protein Desolvation 2.92

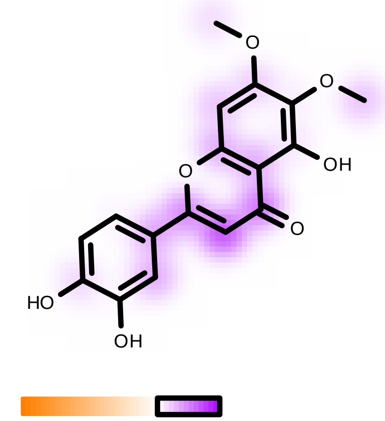

-1.045 0.000 0.482

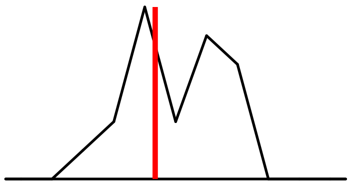

Ligand Desolvation 2.50

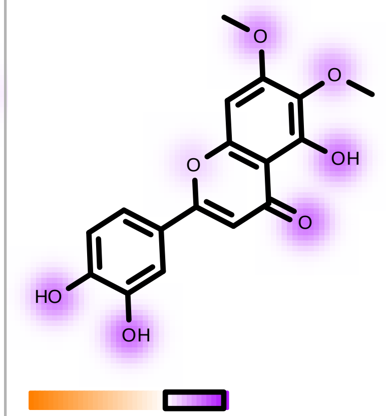

-1.045 0.000 0.482

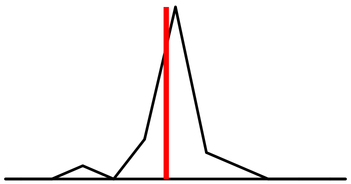

Molecule Name	omega_1_12
Molecular Weight	330.3
XLogP	1.2
PSA	109.4
Heavy Atoms	24
Acceptor Count	7
Donor Count	3
Chelator Count	3
Pose Index	20

Total Score -10.14

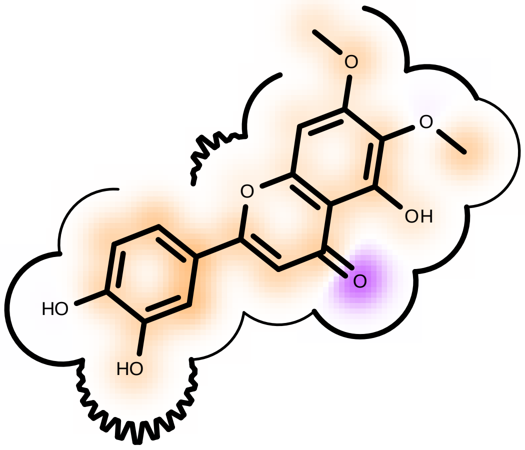

Score compared to other molecules

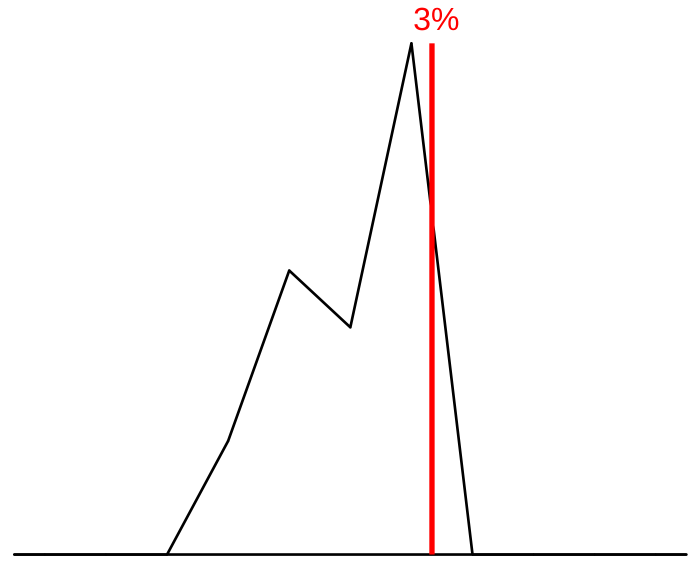

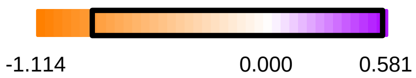

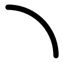 Protein Contact

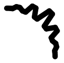 Protein Cavity

Residue Fingerprint

ALA256G	ALA278E
ALA278F	ALA293B
ALA293C	ALA336B
ARG252G	ARG291B
ASP316F	ASP333B
GLN255G	GLU264G
**GLU292B**	GLU292C
GLY290B	ILE263G
LYS260G	THR259G
VAL279E	**VAL279F**

Acceptor	Donor
Metal	Contact

Shape -14.00

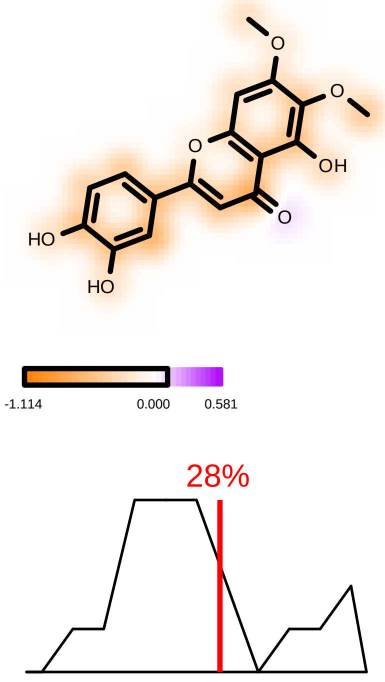

Hydrogen Bond -1.81

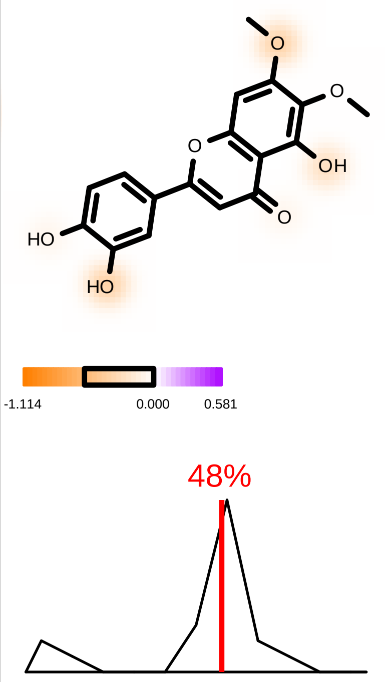

Protein Desolvation 2.91

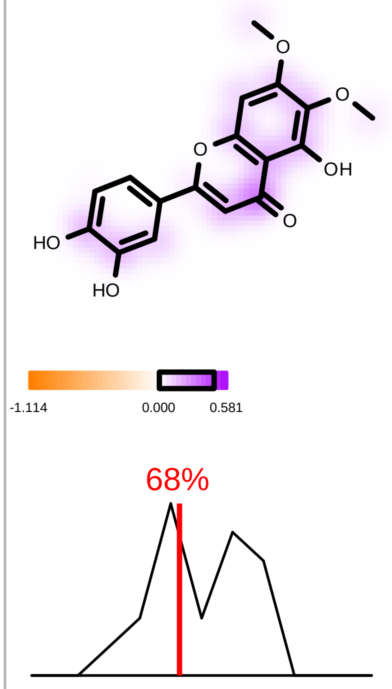

Ligand Desolvation 2.76

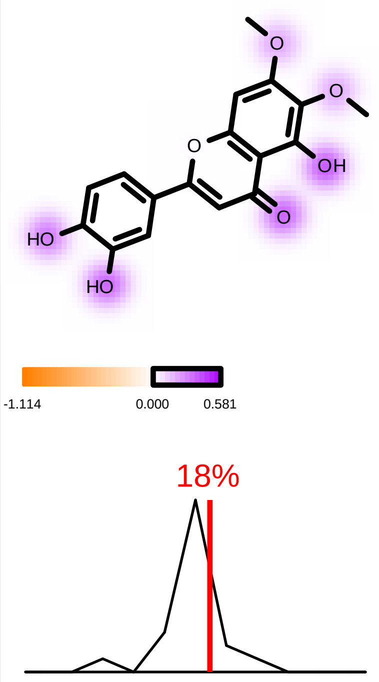

Supplement: Supplementary file 1 [file cells-11-03169-s001.zip › Carlini et al_Supplementary Data S1_FRED_2jj2.pdf]
