# Supplementary material for: The Flavone Cirsiliol from Salvia x jamensis Binds the F1 Moiety of ATP Synthase, Modulating Free Radical Production
Source: Cells. 2022 Oct 9;11(19):3169. doi: 10.3390/cells11193169 (PMC9562182; doi:10.3390/cells11193169)

Molecule Name	omega_1_4
Molecular Weight	330.3
XLogP	1.2
PSA	109.4
Heavy Atoms	24
Acceptor Count	7
Donor Count	3
Chelator Count	3
Pose Index	1

Total Score -10.70

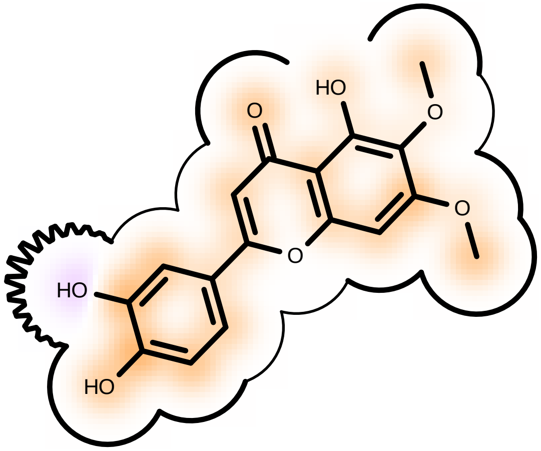

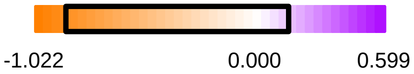

Score compared to other molecules

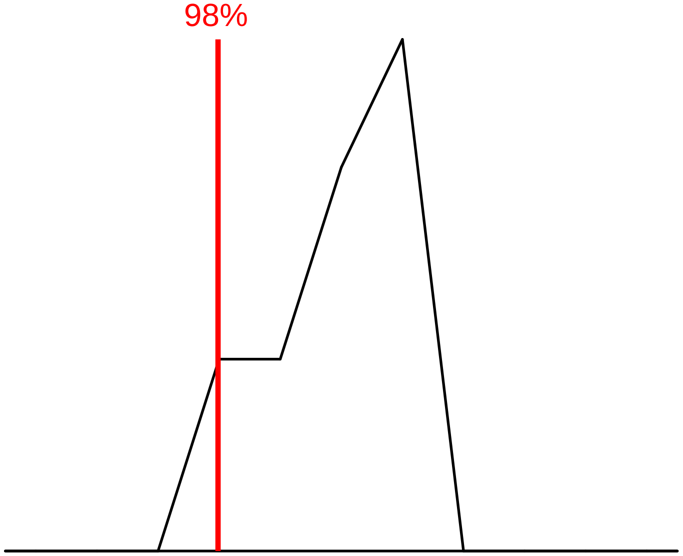

Protein Contact

Protein Cavity

Residue Fingerprint

ALA256G	ALA278E
ALA278F	ALA293B
ALA293C	ALA336B
ARG252G	ARG279C
**ARG291B**	ASP316F
ASP333B	GLN255G
GLU264G	GLU292B
GLU292C	GLY290B
GLY290C	ILE263G
ILE338C	LYS260G
PRO276F	PRO289C
SER267G	SER277F
**THR259G**	TYR294C
VAL279E	**VAL279F**

Acceptor	Donor
Metal	Contact

Shape -14.75

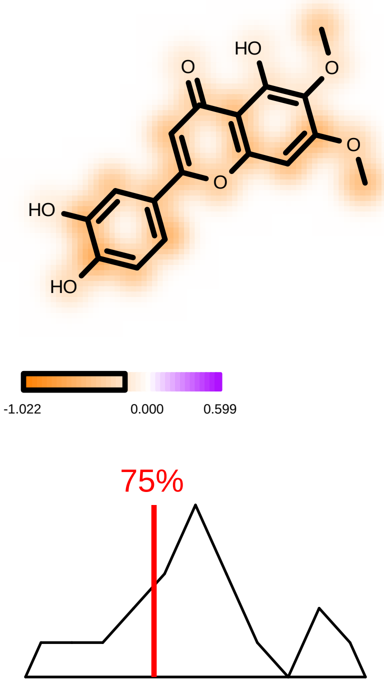

Hydrogen Bond -1.97

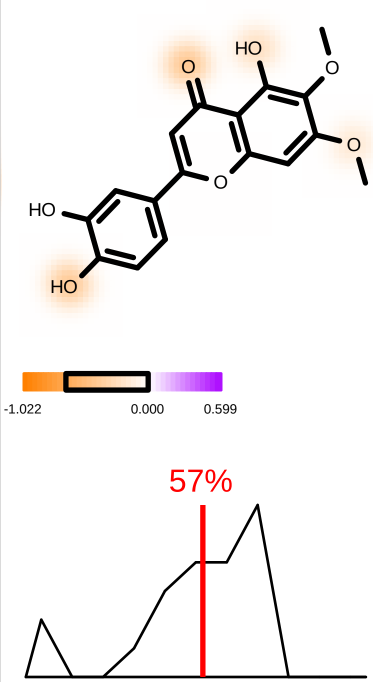

Protein Desolvation 3.33

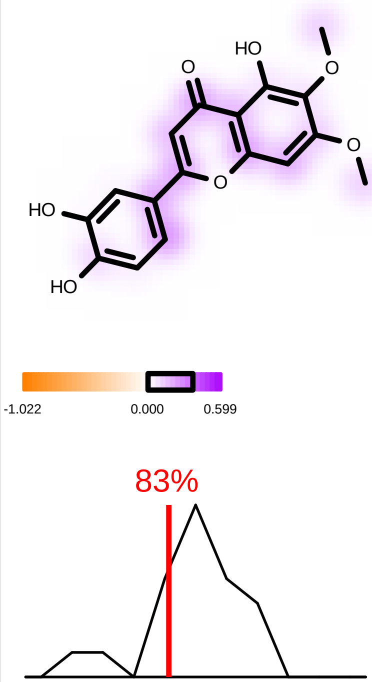

Ligand Desolvation 2.69

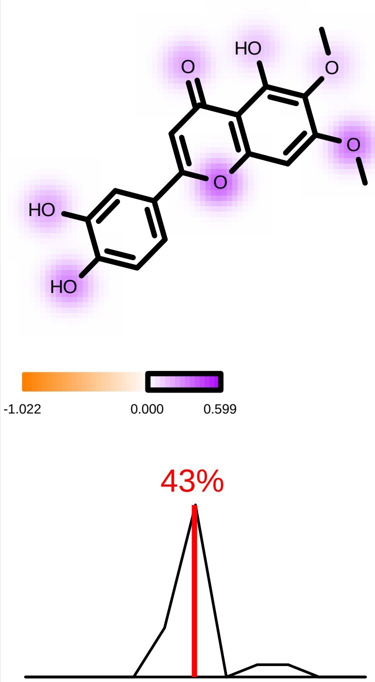

Molecule Name	omega_1_10
Molecular Weight	330.3
XLogP	1.2
PSA	109.4
Heavy Atoms	24
Acceptor Count	7
Donor Count	3
Chelator Count	3
Pose Index	2

Total Score -10.65

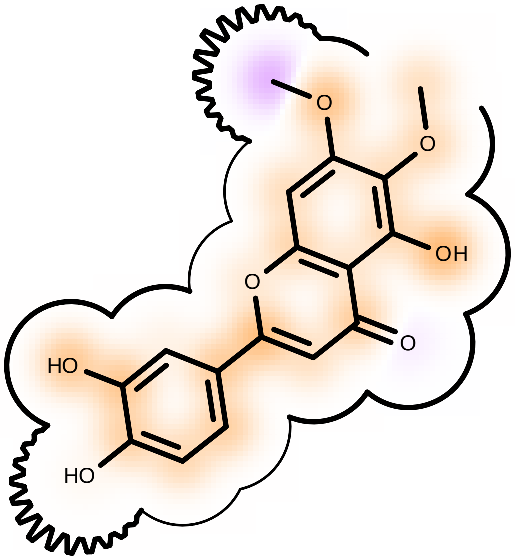

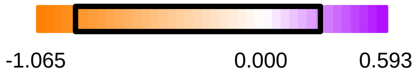

Protein Contact

Protein Cavity

Score compared to other molecules

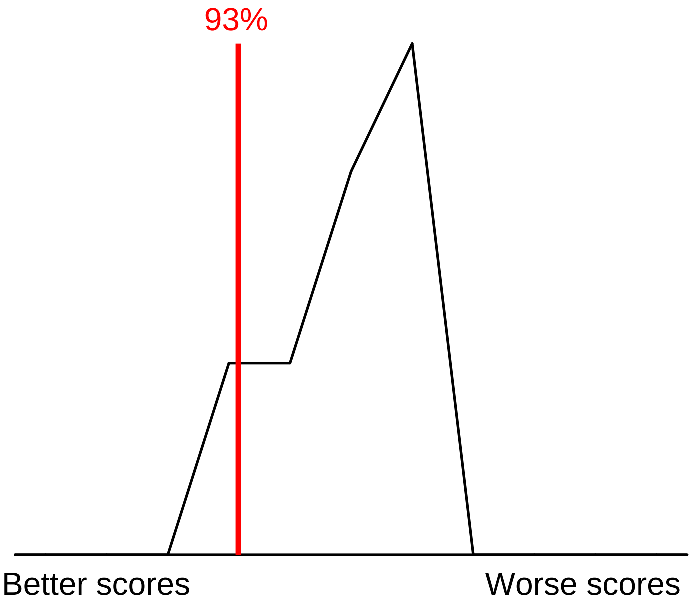

Residue Fingerprint

ALA256G	ALA278E
ALA278F	ALA293B
ALA293C	ALA336B
ARG252G	ARG279C
ARG291B	ASP316F
ASP333B	GLN255G
GLU264G	GLU292B
GLU292C	GLY290B
GLY290C	ILE263G
ILE338C	LYS260G
PRO276F	PRO289C
SER267G	SER277F
THR259G	TYR294C
VAL279E	VAL279F

Acceptor Donor
Metal Contact

Shape -14.44

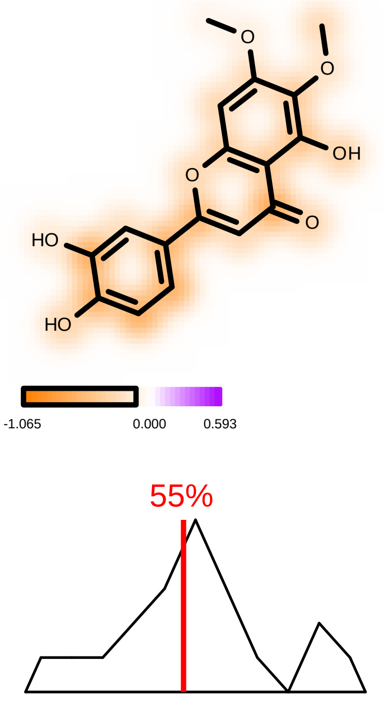

Hydrogen Bond -3.58

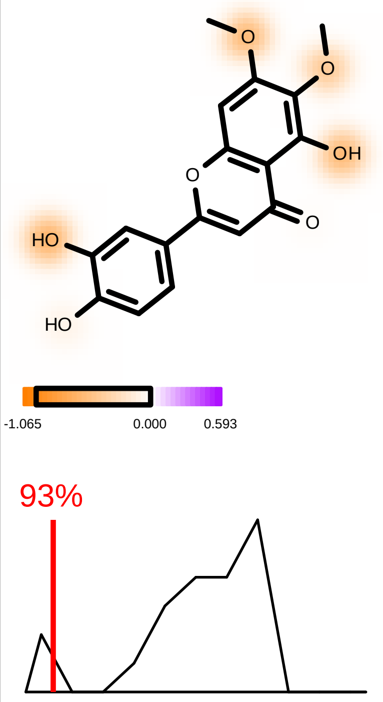

Protein Desolvation 4.10

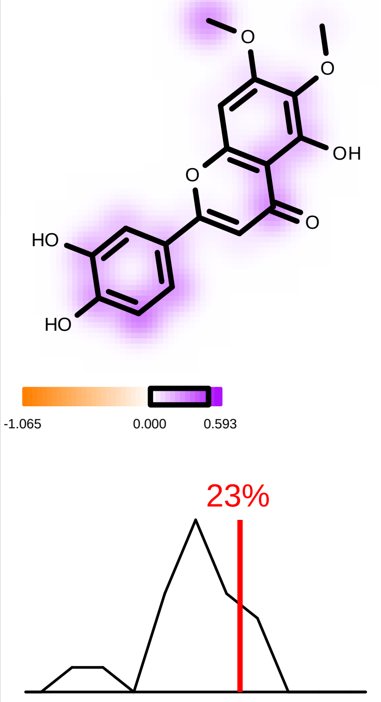

Ligand Desolvation 3.27

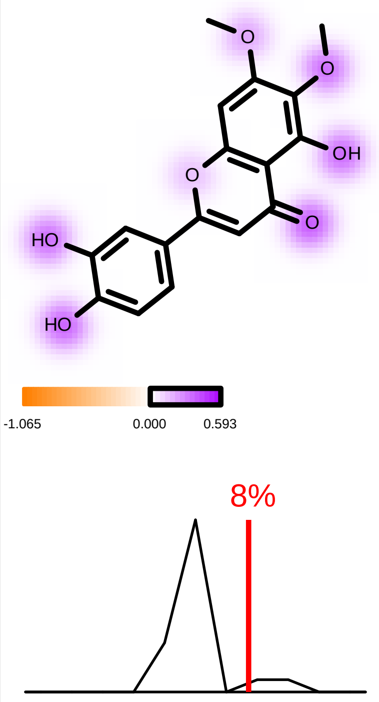

Molecule Name	omega_1_4
Molecular Weight	330.3
XLogP	1.2
PSA	109.4
Heavy Atoms	24
Acceptor Count	7
Donor Count	3
Chelator Count	3
Pose Index	3

Total Score -10.61

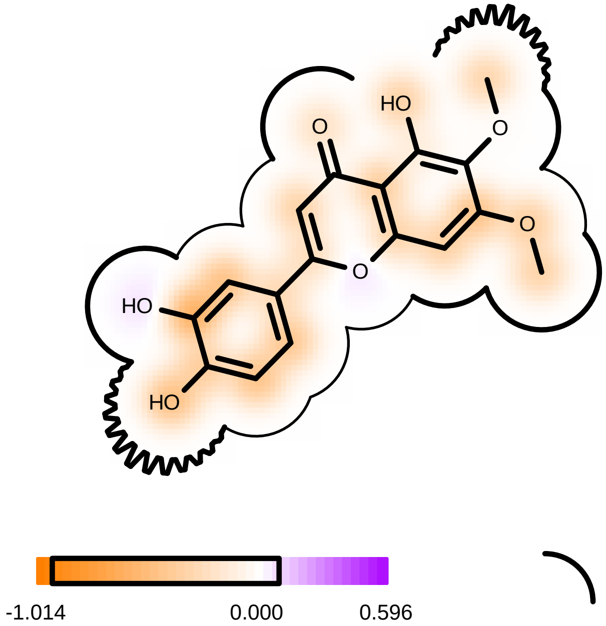

Score compared to other molecules

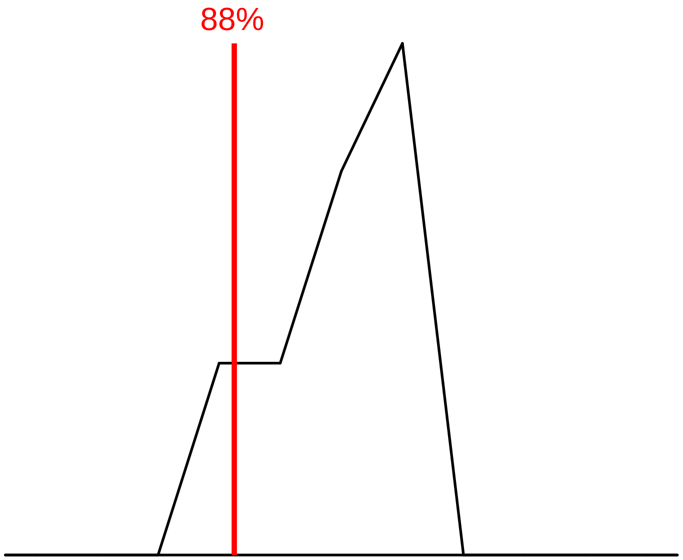

Protein Contact

Protein Cavity

Residue Fingerprint

ALA256G	ALA278E
ALA278F	ALA293B
ALA293C	ALA336B
ARG252G	ARG279C
ARG291B	ASP316F
ASP333B	GLN255G
GLU264G	GLU292B
GLU292C	GLY290B
GLY290C	ILE263G
ILE338C	LYS260G
PRO276F	PRO289C
SER267G	SER277F
THR259G	TYR294C
VAL279E	VAL279F

Acceptor	Donor
Metal	Contact

Shape -14.34

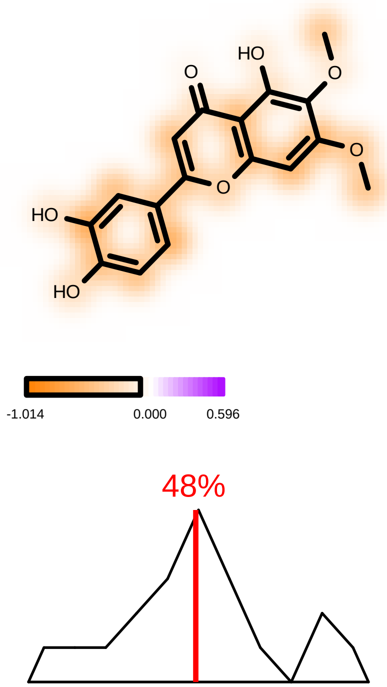

Hydrogen Bond -2.40

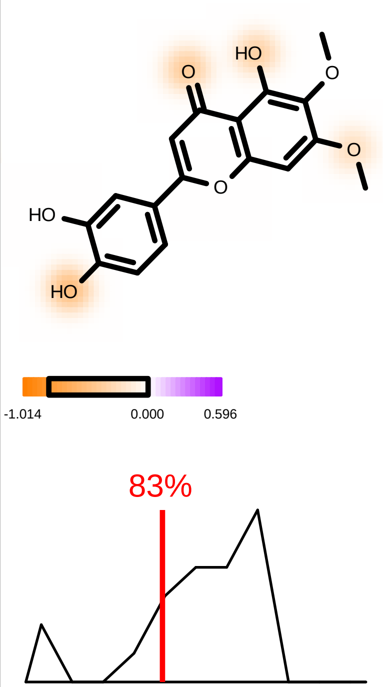

Protein Desolvation 3.36

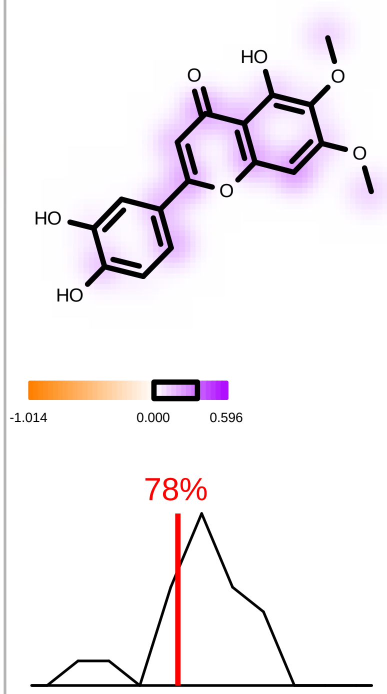

Ligand Desolvation 2.76

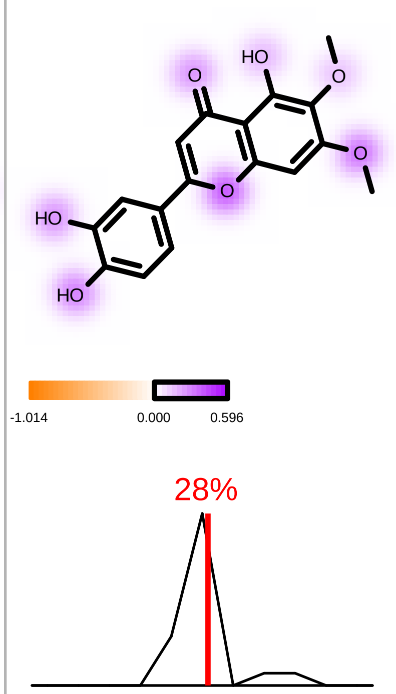

Molecule Name	omega_1_13
Molecular Weight	330.3
XLogP	1.2
PSA	109.4
Heavy Atoms	24
Acceptor Count	7
Donor Count	3
Chelator Count	3
Pose Index	4

Total Score -10.41

Score compared to other molecules

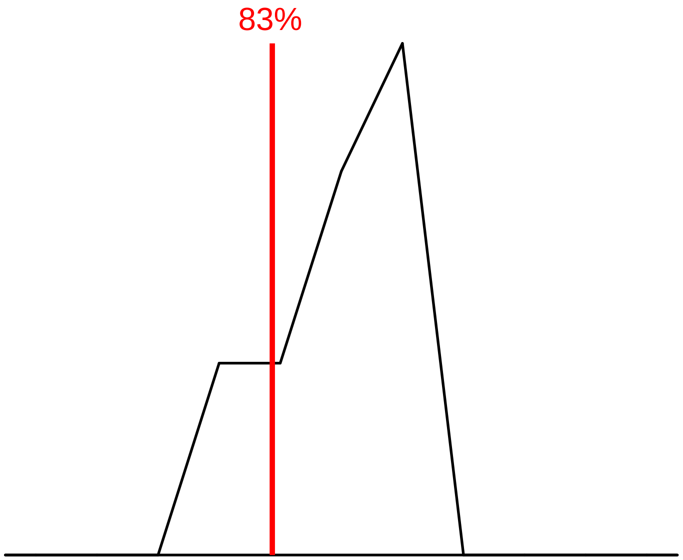

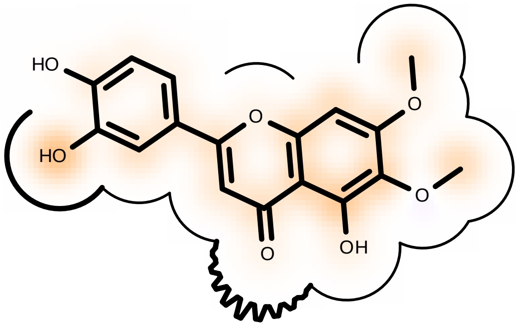

-1.484 0.000 0.599

Protein Contact

Protein Cavity

Residue Fingerprint

ALA256G	ALA278E
ALA278F	ALA293B
ALA293C	ALA336B
ARG252G	ARG279C
ARG291B	ASP316F
ASP333B	GLN255G
GLU264G	GLU292B
GLU292C	GLY290B
GLY290C	ILE263G
ILE338C	LYS260G
PRO276F	PRO289C
SER267G	SER277F
THR259G	TYR294C
VAL279E	VAL279F

Acceptor Donor
Metal Contact

Shape -12.97

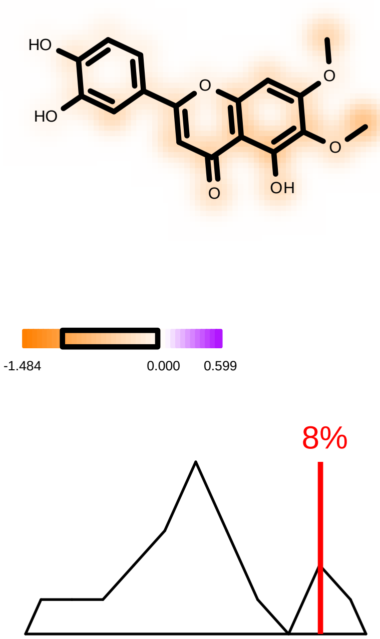

Hydrogen Bond -1.90

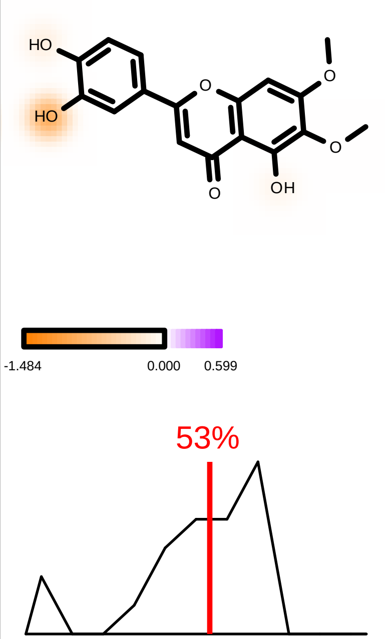

Protein Desolvation 2.14

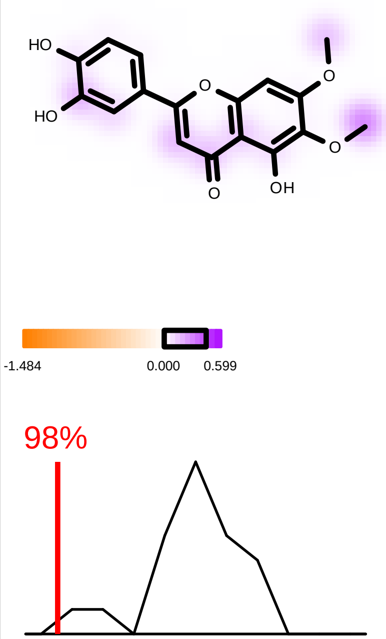

Ligand Desolvation 2.32

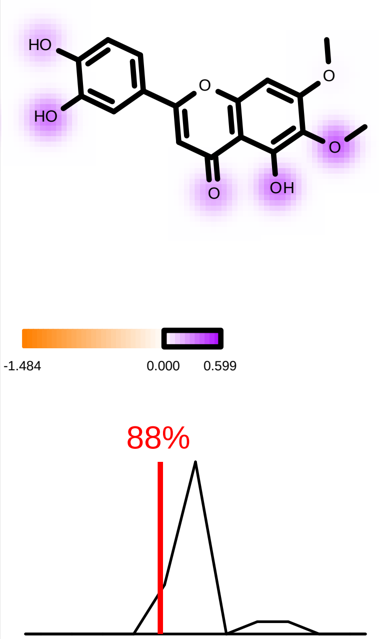

Molecule Name	omega_1_1
Molecular Weight	330.3
XLogP	1.2
PSA	109.4
Heavy Atoms	24
Acceptor Count	7
Donor Count	3
Chelator Count	3
Pose Index	5

Total Score -10.38

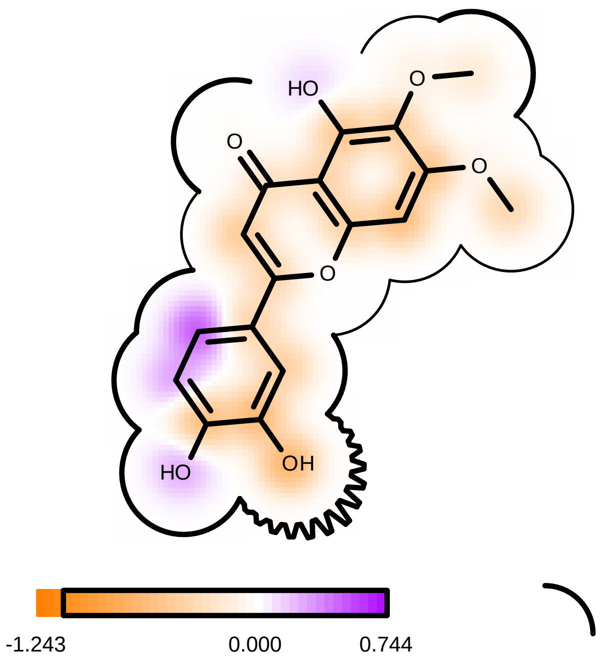

Score compared to other molecules

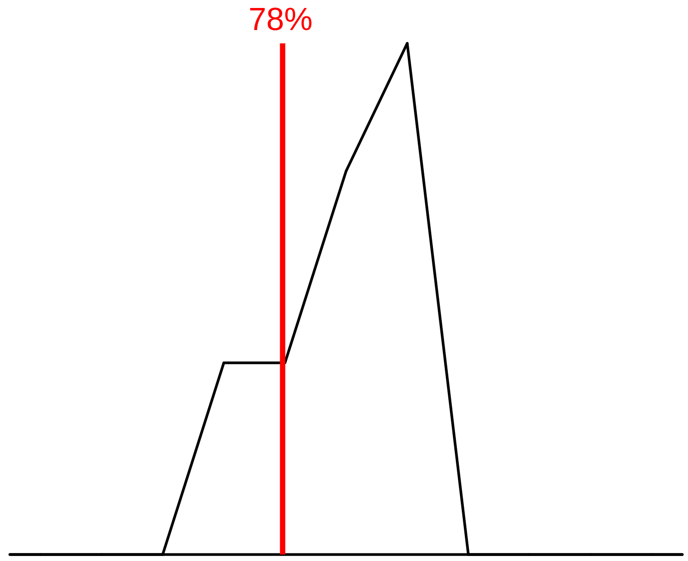

Protein Contact

Protein Cavity

Residue Fingerprint

ALA256G	ALA278E
ALA278F	ALA293B
ALA293C	ALA336B
ARG252G	ARG279C
ARG291B	ASP316F
ASP333B	GLN255G
GLU264G	GLU292B
GLU292C	GLY290B
GLY290C	ILE263G
ILE338C	LYS260G
PRO276F	PRO289C
SER267G	SER277F
THR259G	TYR294C
VAL279E	VAL279F

Acceptor	Donor
Metal	Contact

Shape -15.83

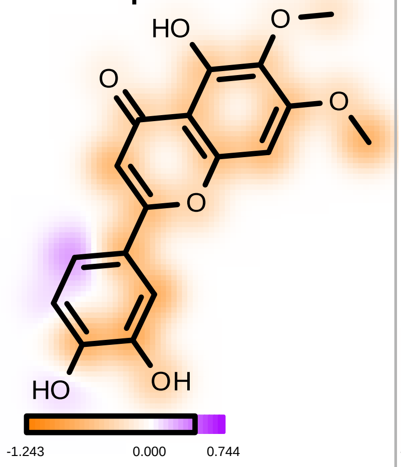

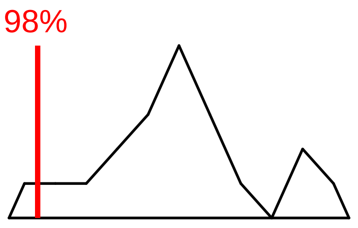

Hydrogen Bond -1.32

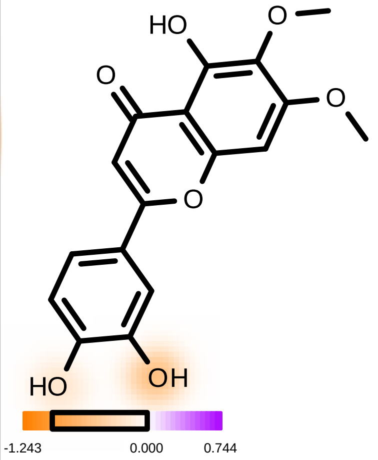

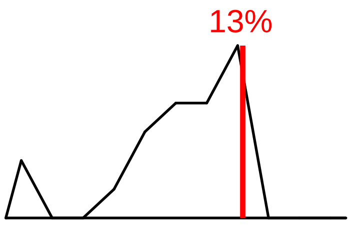

Protein Desolvation 4.11

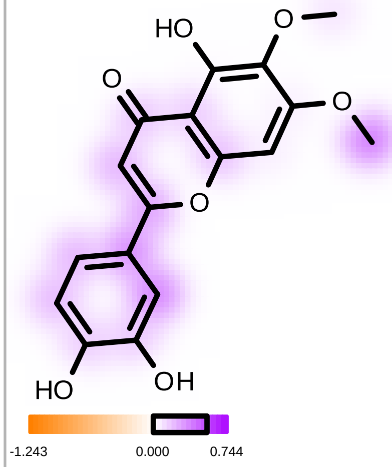

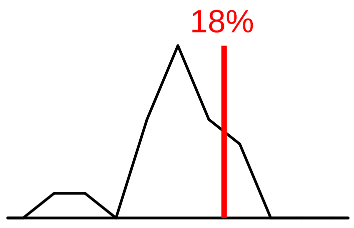

Ligand Desolvation 2.66

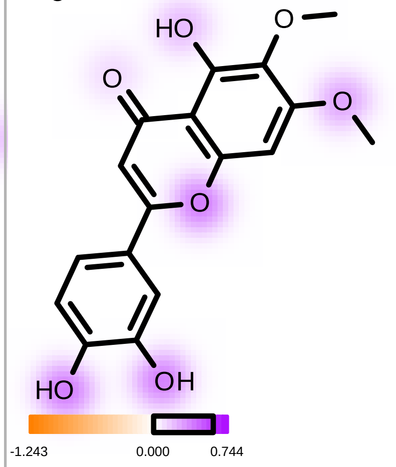

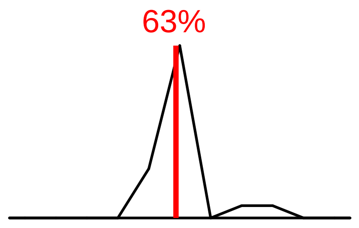

Molecule Name	omega_1_1
Molecular Weight	330.3
XLogP	1.2
PSA	109.4
Heavy Atoms	24
Acceptor Count	7
Donor Count	3
Chelator Count	3
Pose Index	6

Total Score -10.23

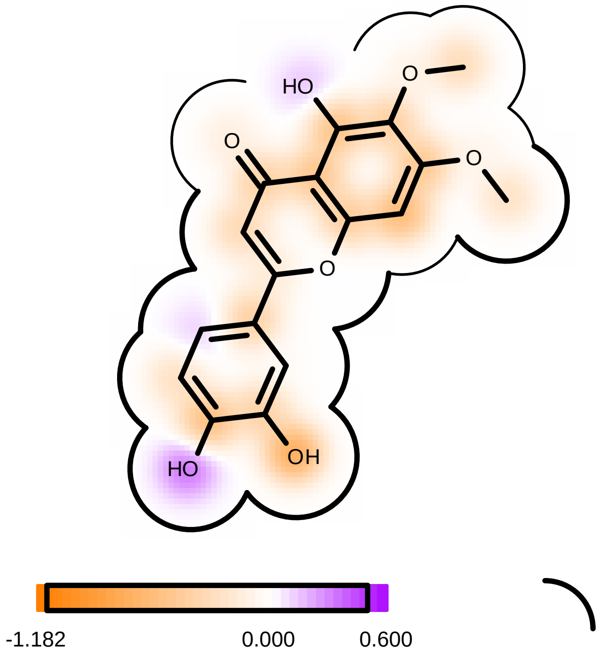

Score compared to other molecules

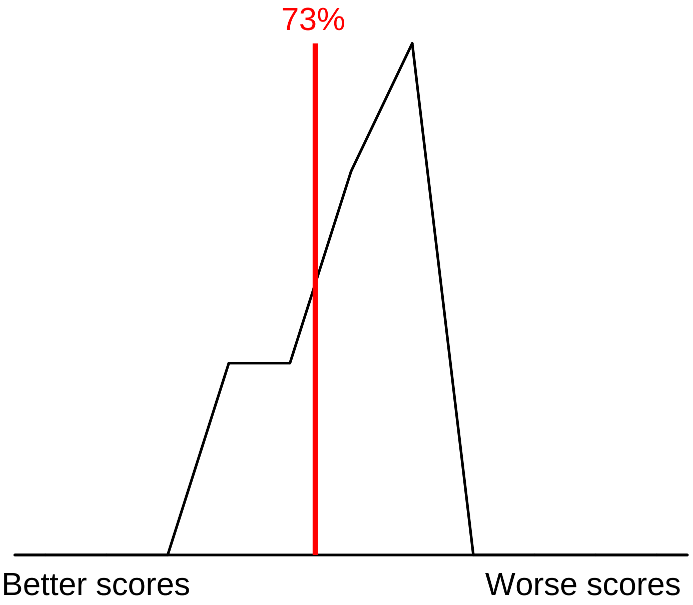

Protein Contact

Protein Cavity

Residue Fingerprint

ALA256G	ALA278E
ALA278F	ALA293B
ALA293C	ALA336B
ARG252G	ARG279C
ARG291B	ASP316F
ASP333B	GLN255G
GLU264G	GLU292B
GLU292C	GLY290B
GLY290C	ILE263G
ILE338C	LYS260G
PRO276F	PRO289C
SER267G	SER277F
THR259G	TYR294C
VAL279E	VAL279F

Acceptor	Donor
Metal	Contact

Shape -15.78

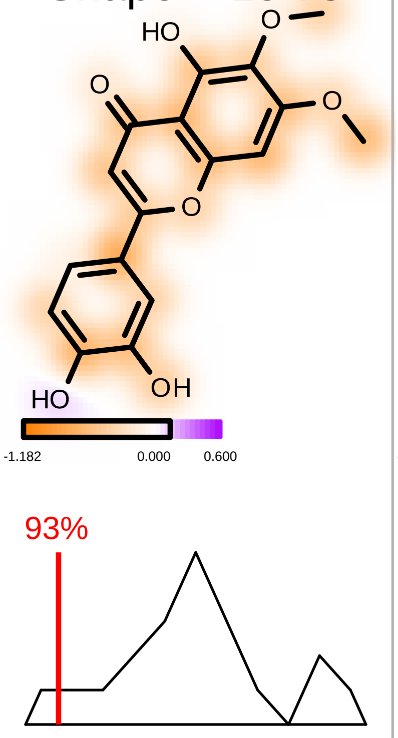

Hydrogen Bond -1.26

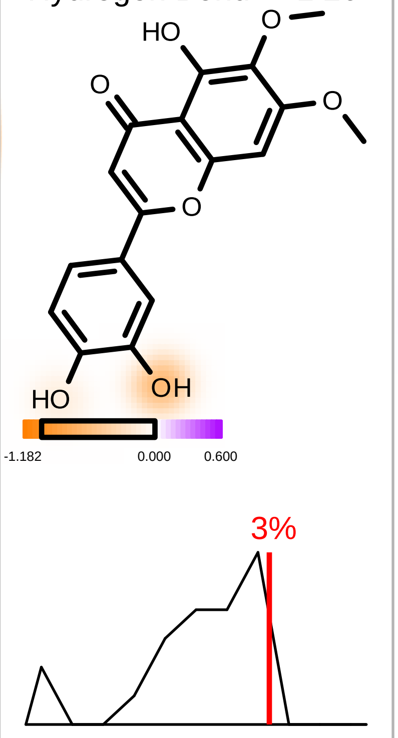

Protein Desolvation 4.27

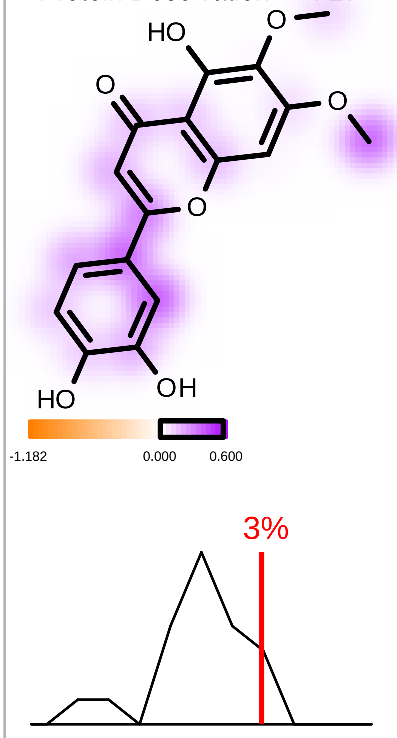

Ligand Desolvation 2.55

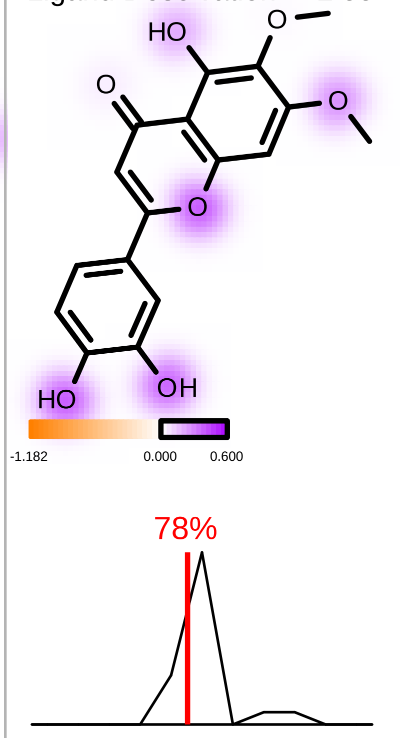

Molecule Name	omega_1_16
Molecular Weight	330.3
XLogP	1.2
PSA	109.4
Heavy Atoms	24
Acceptor Count	7
Donor Count	3
Chelator Count	3
Pose Index	7

Total Score -10.11

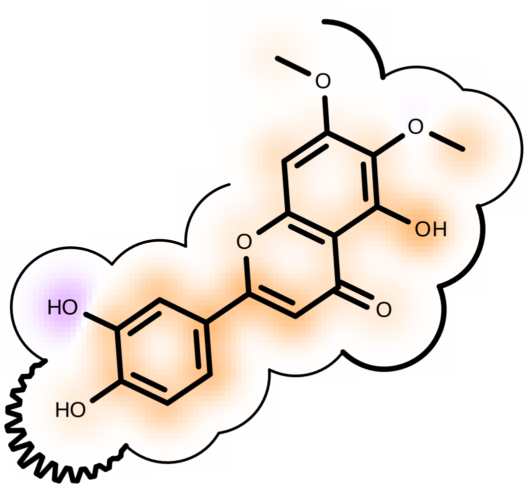

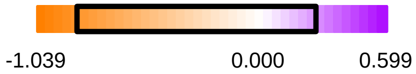

Score compared to other molecules

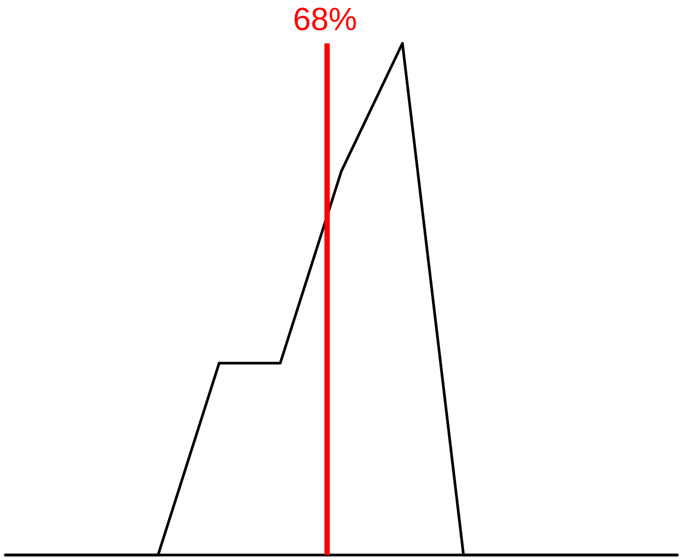

Protein Contact

Protein Cavity

Residue Fingerprint

ALA256G	ALA278E
ALA278F	ALA293B
ALA293C	ALA336B
ARG252G	ARG279C
ARG291B	ASP316F
ASP333B	GLN255G
GLU264G	**GLU292B**
GLU292C	GLY290B
GLY290C	ILE263G
ILE338C	LYS260G
PRO276F	PRO289C
SER267G	SER277F
THR259G	TYR294C
VAL279E	VAL279F

Acceptor	Donor
Metal	Contact

Shape -14.92

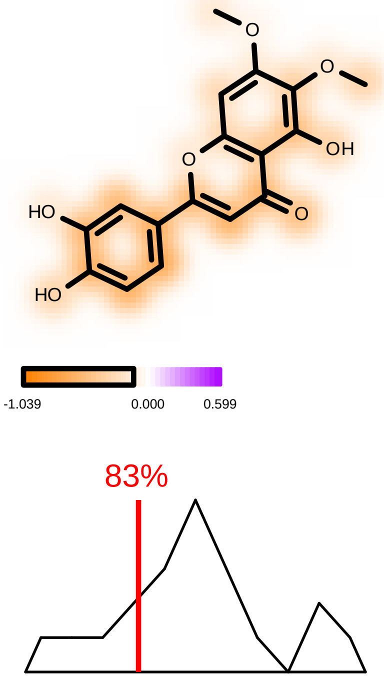

Hydrogen Bond -1.26

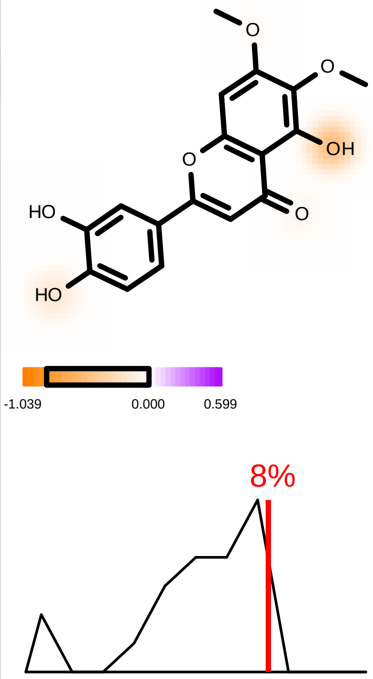

Protein Desolvation 3.41

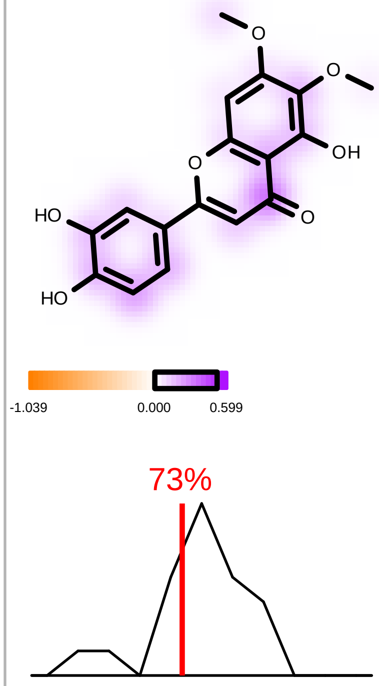

Ligand Desolvation 2.67

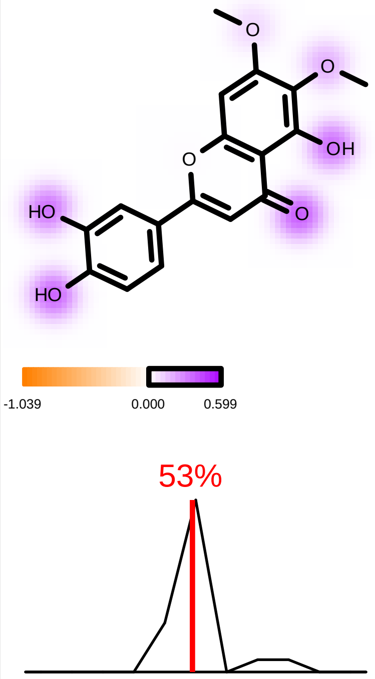

Molecule Name	omega_1_14
Molecular Weight	330.3
XLogP	1.2
PSA	109.4
Heavy Atoms	24
Acceptor Count	7
Donor Count	3
Chelator Count	3
Pose Index	8

Total Score -10.05

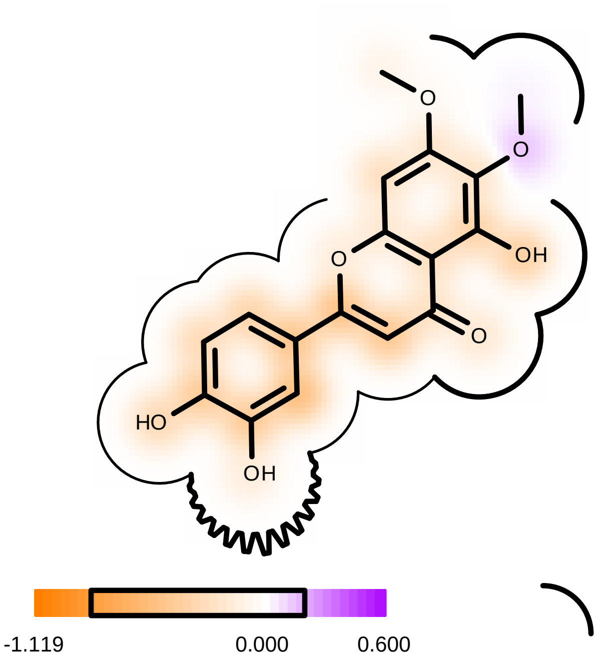

Score compared to other molecules

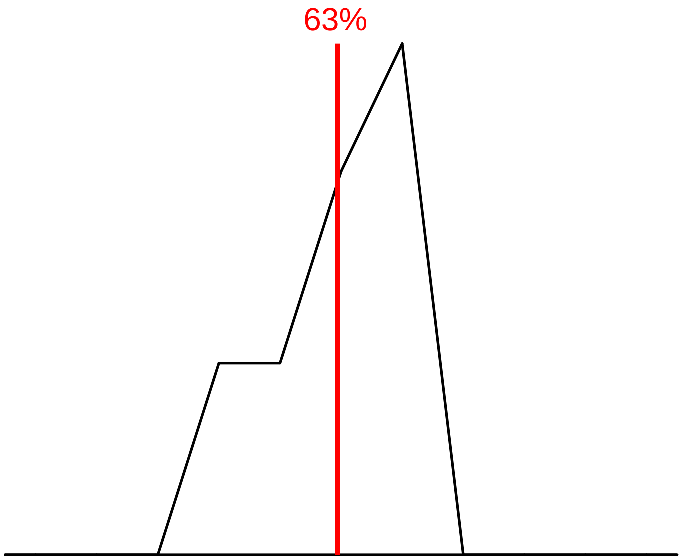

Protein Contact

Protein Cavity

Residue Fingerprint

ALA256G	ALA278E
ALA278F	ALA293B
ALA293C	ALA336B
ARG252G	ARG279C
ARG291B	ASP316F
ASP333B	GLN255G
GLU264G	GLU292B
GLU292C	GLY290B
GLY290C	ILE263G
ILE338C	LYS260G
PRO276F	PRO289C
SER267G	SER277F
THR259G	TYR294C
VAL279E	VAL279F

Acceptor Donor
Metal Contact

Shape -14.55

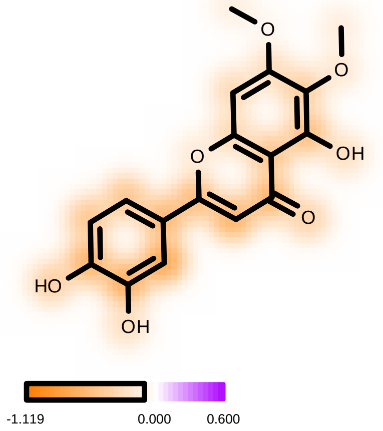

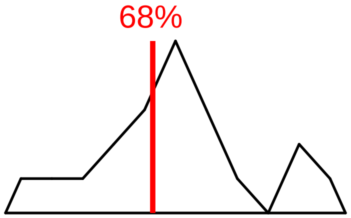

Hydrogen Bond -1.87

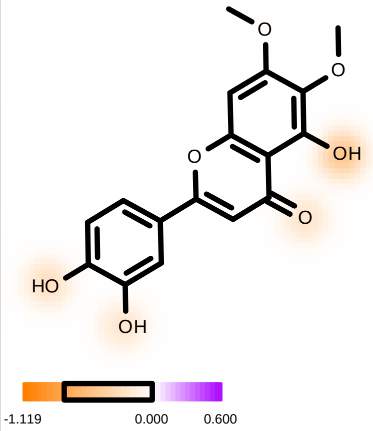

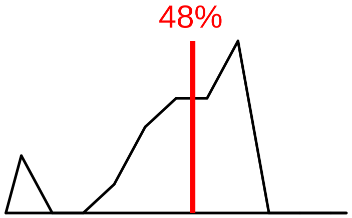

Protein Desolvation 3.69

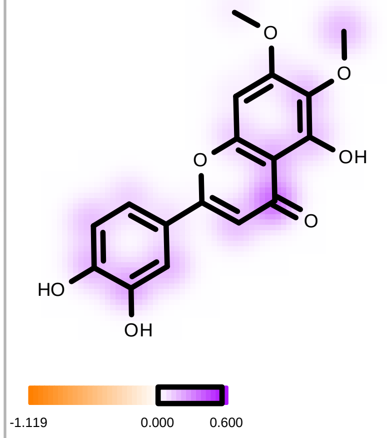

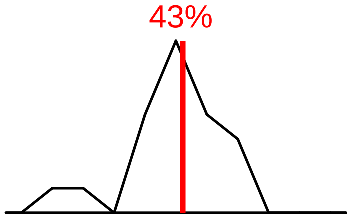

Ligand Desolvation 2.67

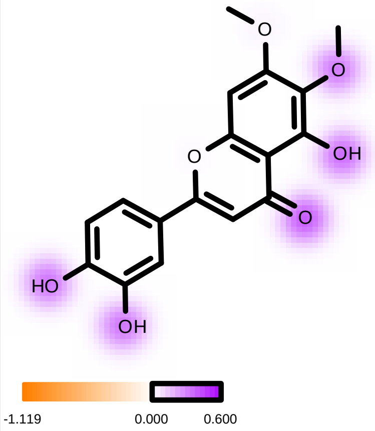

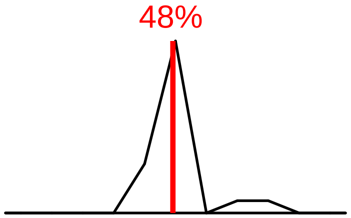

Molecule Name	omega_1_3
Molecular Weight	330.3
XLogP	1.2
PSA	109.4
Heavy Atoms	24
Acceptor Count	7
Donor Count	3
Chelator Count	3
Pose Index	9

Total Score -10.01

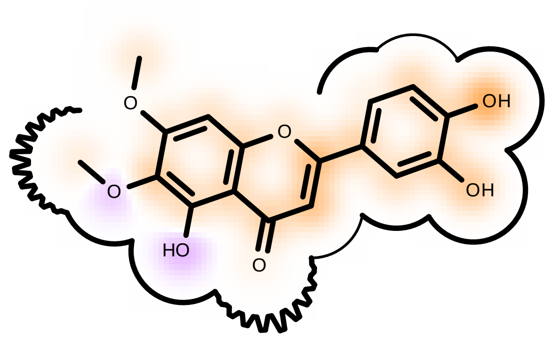

Score compared to other molecules

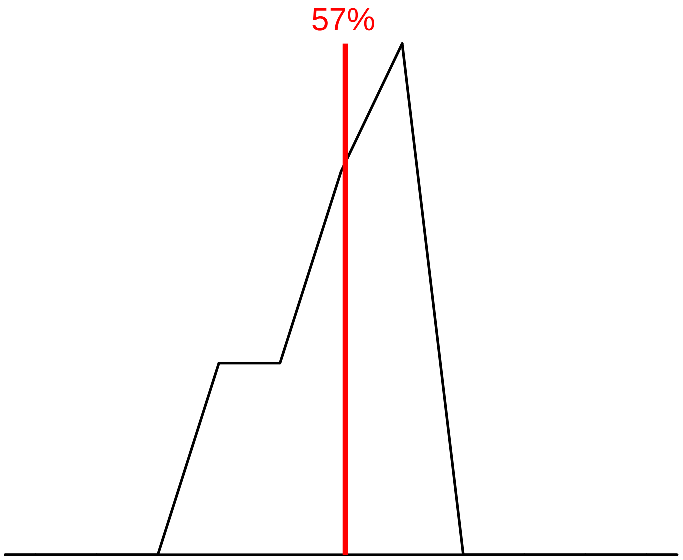

-1.107 0.000 0.548

Protein Contact

Protein Cavity

Residue Fingerprint

ALA256G	ALA278E
ALA278F	ALA293B
ALA293C	ALA336B
ARG252G	ARG279C
ARG291B	ASP316F
ASP333B	GLN255G
GLU264G	GLU292B
GLU292C	GLY290B
GLY290C	ILE263G
ILE338C	LYS260G
PRO276F	PRO289C
SER267G	SER277F
THR259G	TYR294C
VAL279E	VAL279F

Acceptor Donor
Metal Contact

Shape -12.50

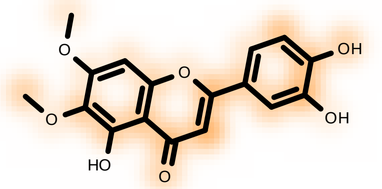

-1.107 0.000 0.548

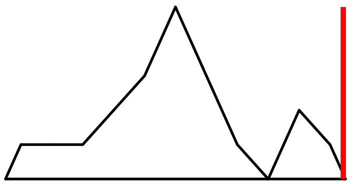

Hydrogen Bond -2.22

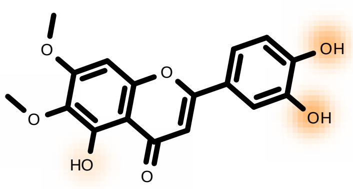

-1.107 0.000 0.548

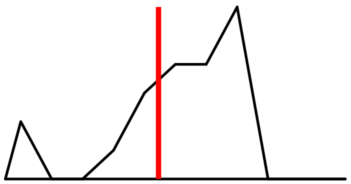

Protein Desolvation 2.50

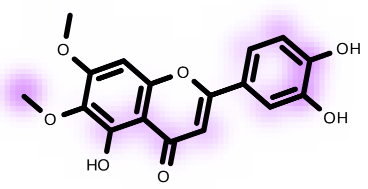

-1.107 0.000 0.548

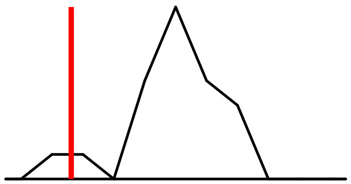

Ligand Desolvation 2.22

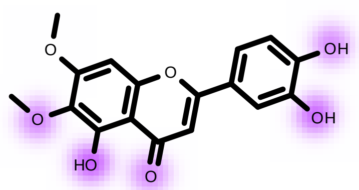

-1.107 0.000 0.548

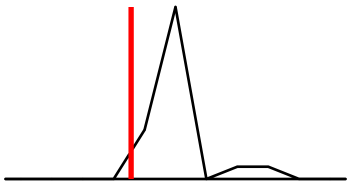

Molecule Name	omega_1_13
Molecular Weight	330.3
XLogP	1.2
PSA	109.4
Heavy Atoms	24
Acceptor Count	7
Donor Count	3
Chelator Count	3
Pose Index	10

Total Score -9.99

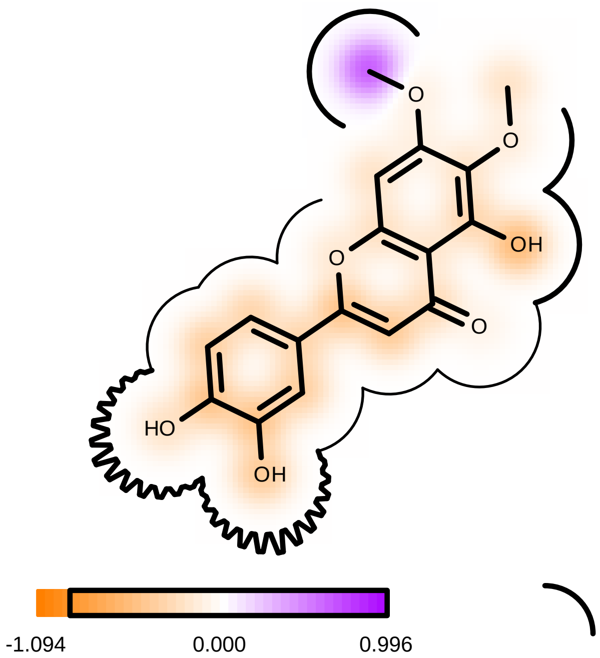

Score compared to other molecules

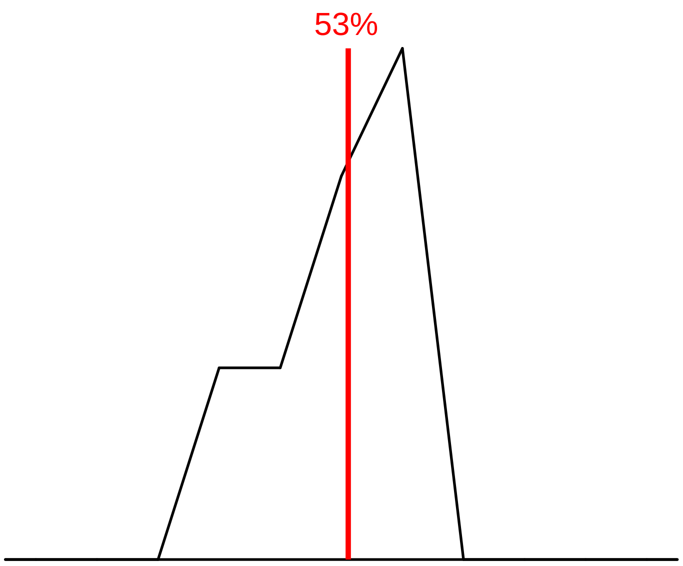

Protein Contact

Protein Cavity

Residue Fingerprint

ALA256G	ALA278E
ALA278F	ALA293B
ALA293C	ALA336B
ARG252G	ARG279C
ARG291B	ASP316F
ASP333B	GLN255G
GLU264G	GLU292B
GLU292C	GLY290B
GLY290C	ILE263G
ILE338C	LYS260G
PRO276F	PRO289C
SER267G	SER277F
THR259G	TYR294C
VAL279E	VAL279F

Acceptor	Donor
Metal	Contact

Shape -13.89

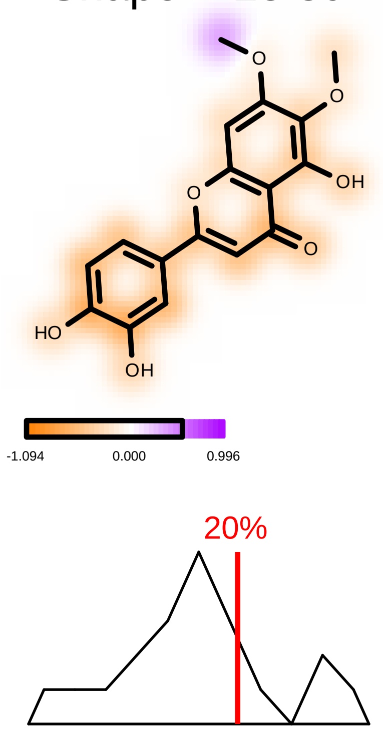

Hydrogen Bond -2.58

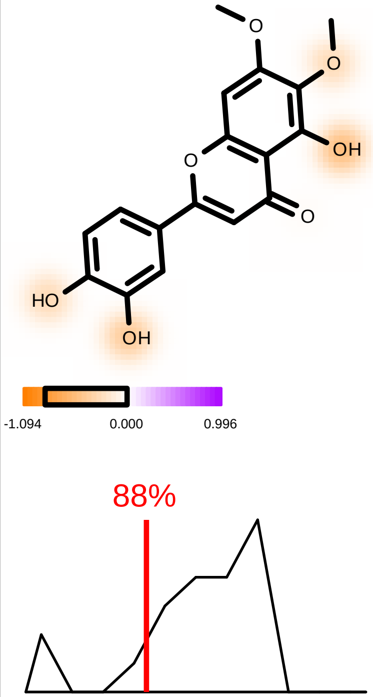

Protein Desolvation 3.67

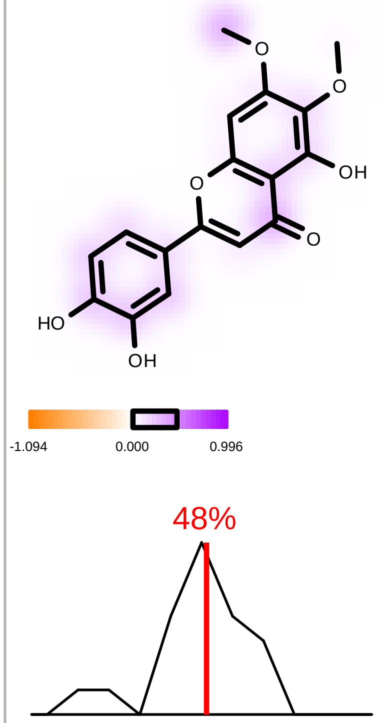

Ligand Desolvation 2.80

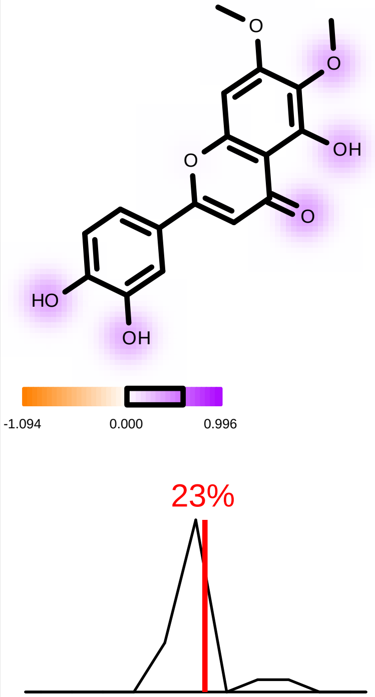

Molecule Name	omega_1_14
Molecular Weight	330.3
XLogP	1.2
PSA	109.4
Heavy Atoms	24
Acceptor Count	7
Donor Count	3
Chelator Count	3
Pose Index	11

Total Score -9.95

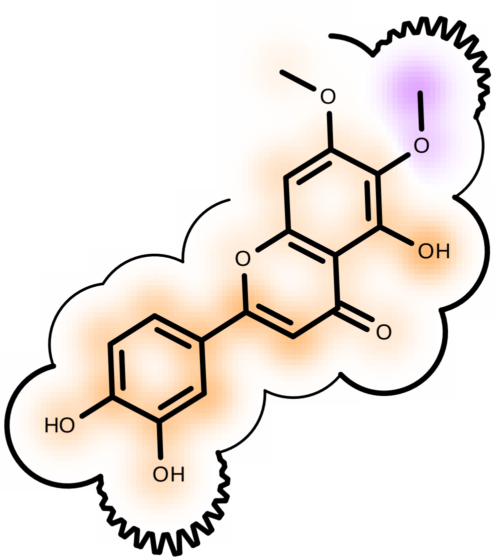

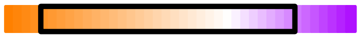

Score compared to other molecules

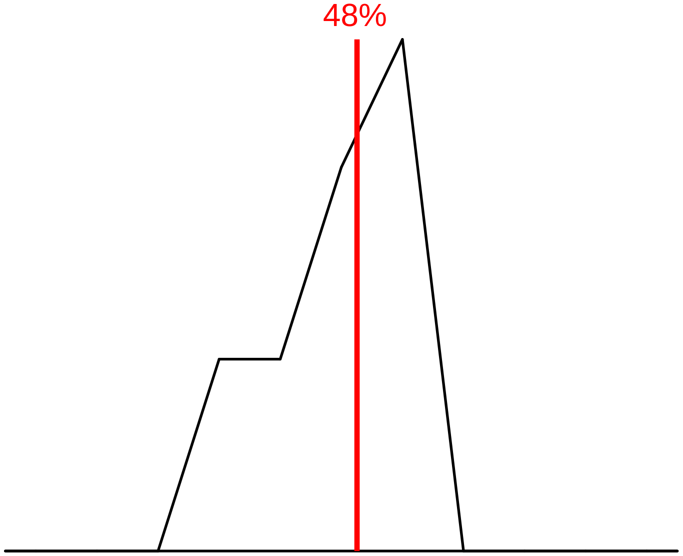

Protein Contact

Protein Cavity

<u>Residue Fingerprint</u>

ALA256G	ALA278E
ALA278F	ALA293B
ALA293C	ALA336B
ARG252G	ARG279C
ARG291B	ASP316F
ASP333B	GLN255G
GLU264G	GLU292B
GLU292C	GLY290B
GLY290C	ILE263G
ILE338C	LYS260G
PRO276F	PRO289C
SER267G	SER277F
THR259G	TYR294C
VAL279E	VAL279F

Acceptor	Donor
Metal	Contact

Shape -14.31

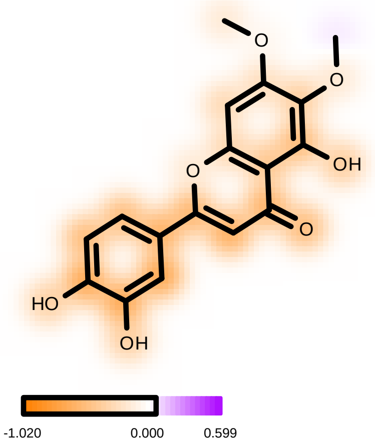

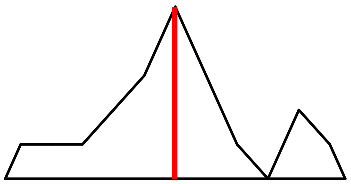

Hydrogen Bond -1.77

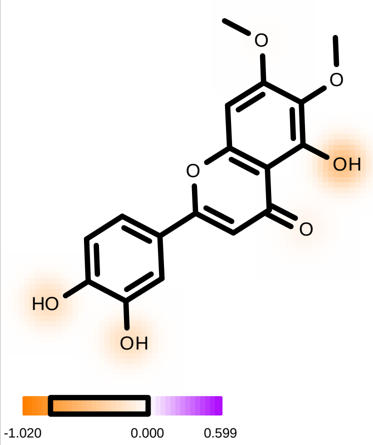

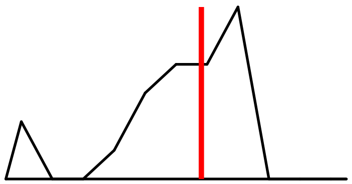

Protein Desolvation 3.48

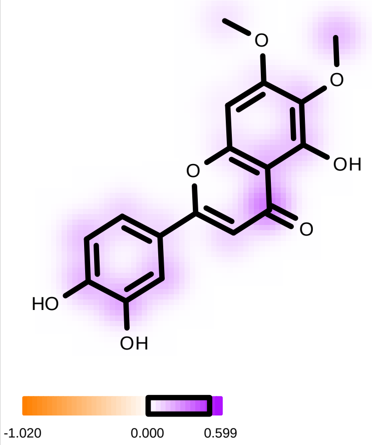

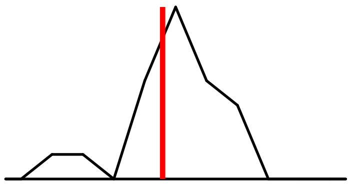

Ligand Desolvation 2.66

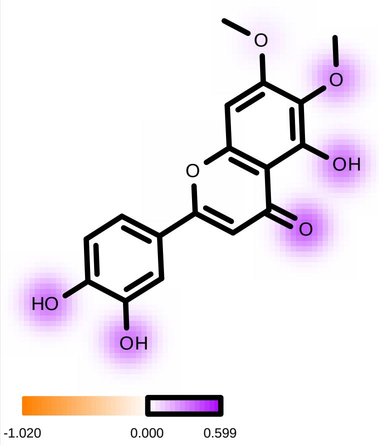

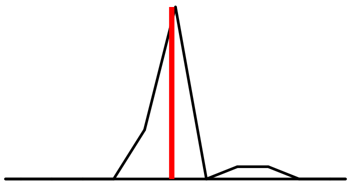

Molecule Name	omega_1_13
Molecular Weight	330.3
XLogP	1.2
PSA	109.4
Heavy Atoms	24
Acceptor Count	7
Donor Count	3
Chelator Count	3
Pose Index	12

Total Score -9.87

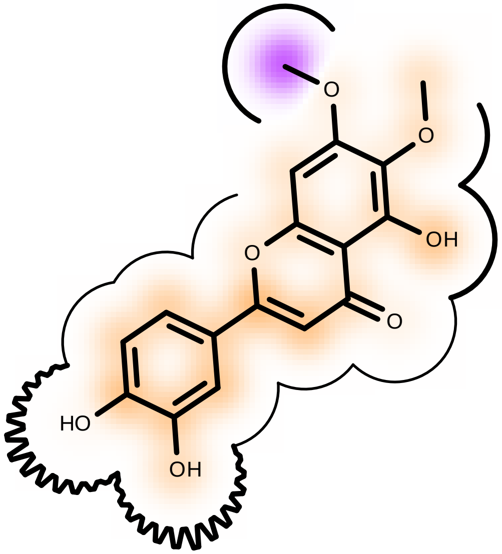

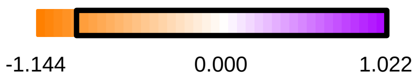

Score compared to other molecules

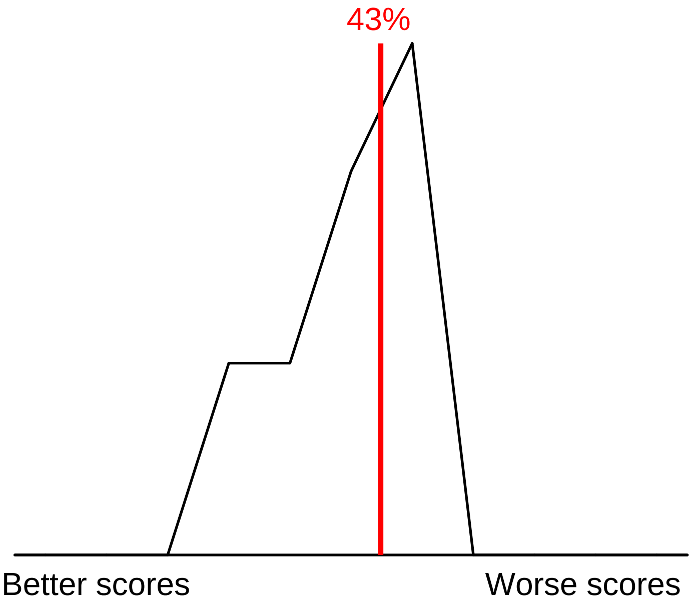

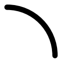
Protein Contact

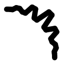
Protein Cavity

Residue Fingerprint

ALA256G	ALA278E
ALA278F	ALA293B
ALA293C	ALA336B
ARG252G	ARG279C
ARG291B	ASP316F
ASP333B	GLN255G
GLU264G	GLU292B
GLU292C	GLY290B
GLY290C	ILE263G
ILE338C	LYS260G
PRO276F	PRO289C
SER267G	SER277F
THR259G	TYR294C
VAL279E	VAL279F

Acceptor	Donor
Metal	Contact

Shape -13.93

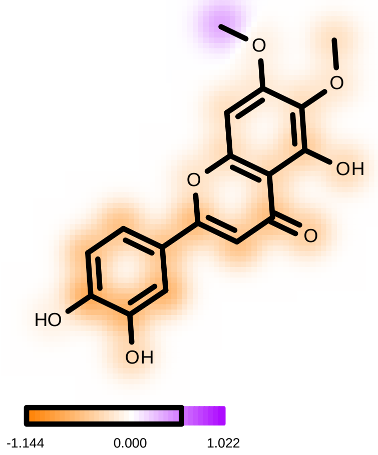

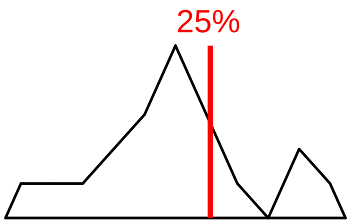

Hydrogen Bond -2.32

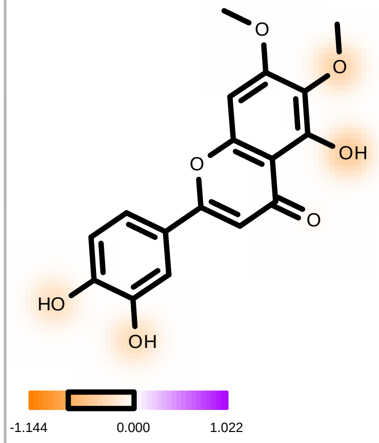

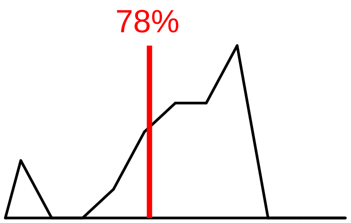

Protein Desolvation 3.64

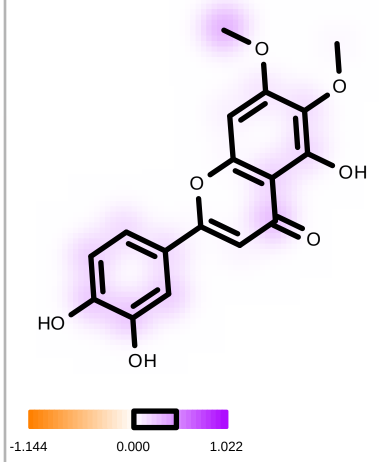

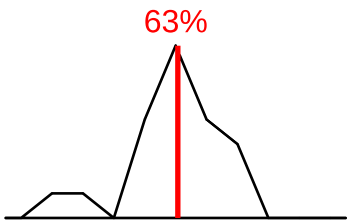

Ligand Desolvation 2.74

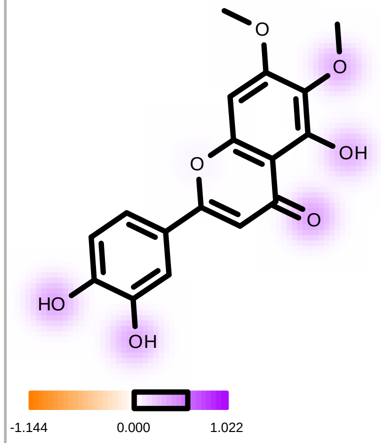

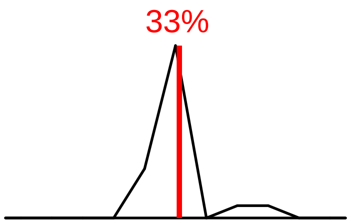

Molecule Name	omega_1_1
Molecular Weight	330.3
XLogP	1.2
PSA	109.4
Heavy Atoms	24
Acceptor Count	7
Donor Count	3
Chelator Count	3
Pose Index	13

Total Score -9.86

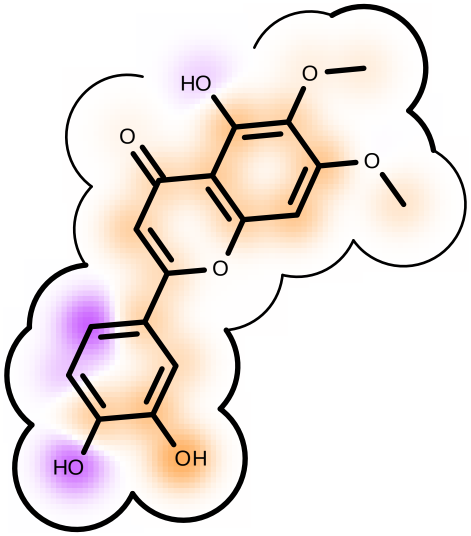

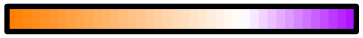

-1.230 0.000 0.608

Score compared to other molecules

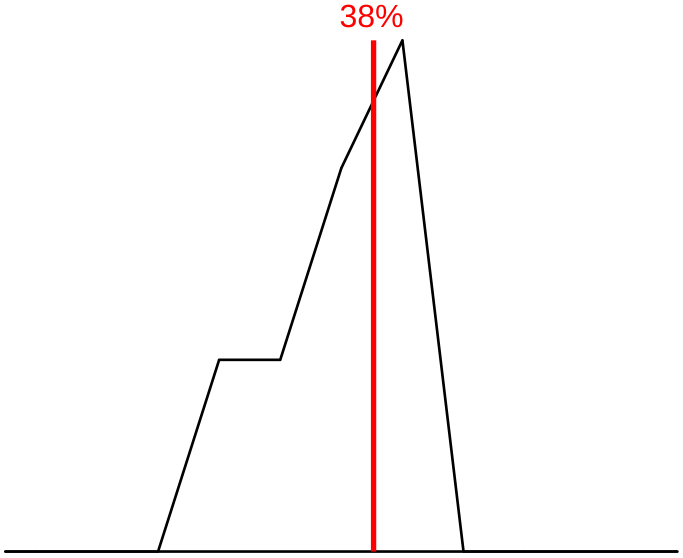

Better scores Worse scores

Protein Contact Protein Cavity

Residue Fingerprint

ALA256G	ALA278E
ALA278F	ALA293B
ALA293C	ALA336B
ARG252G	ARG279C
ARG291B	ASP316F
ASP333B	GLN255G
**GLU264G**	GLU292B
GLU292C	GLY290B
GLY290C	ILE263G
ILE338C	LYS260G
PRO276F	PRO289C
**SER267G**	SER277F
THR259G	TYR294C
VAL279E	VAL279F

Acceptor	Donor
Metal	Contact

Shape -15.44

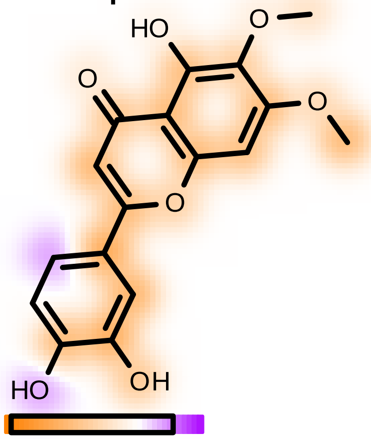

-1.230 0.000 0.608

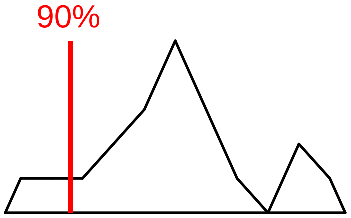

Hydrogen Bond -1.39

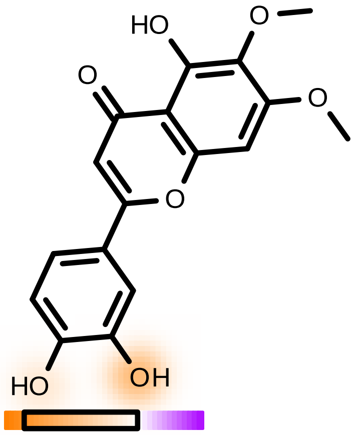

-1.230 0.000 0.608

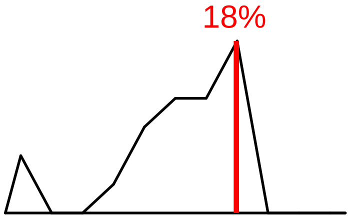

Protein Desolvation 4.26

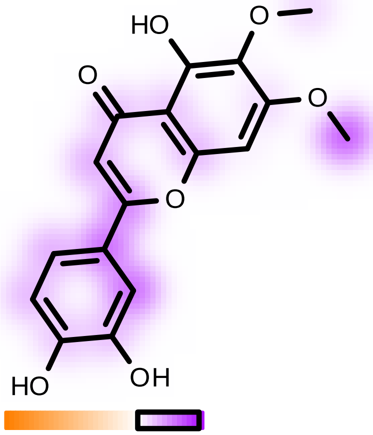

-1.230 0.000 0.608

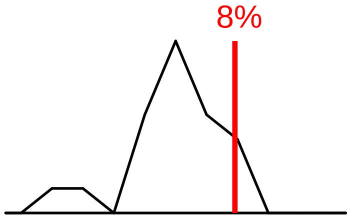

Ligand Desolvation 2.71

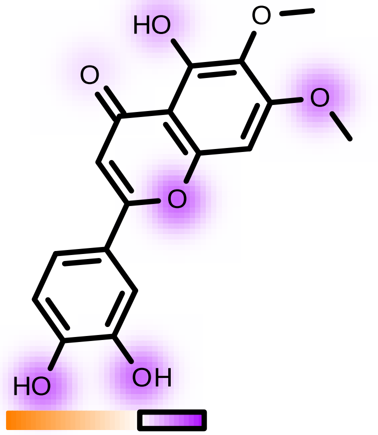

-1.230 0.000 0.608

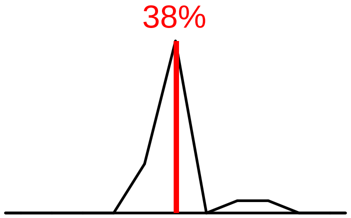

Molecule Name	omega_1_16
Molecular Weight	330.3
XLogP	1.2
PSA	109.4
Heavy Atoms	24
Acceptor Count	7
Donor Count	3
Chelator Count	3
Pose Index	14

Total Score -9.84

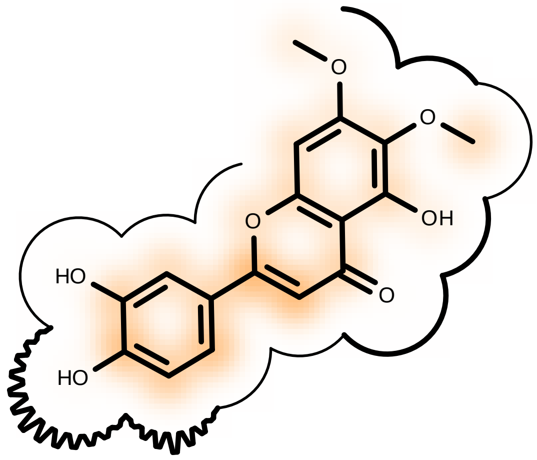

Score compared to other molecules

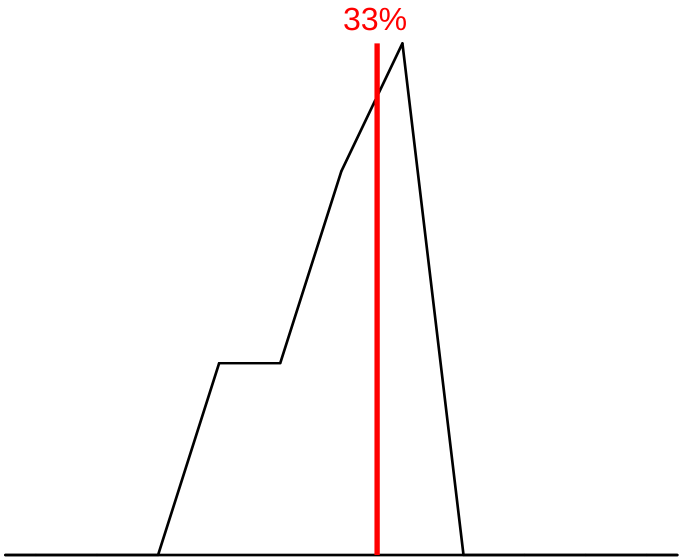

-1.135 0.000 0.600

Protein Contact

Protein Cavity

Residue Fingerprint

ALA256G	ALA278E
ALA278F	ALA293B
ALA293C	ALA336B
ARG252G	ARG279C
ARG291B	ASP316F
ASP333B	GLN255G
GLU264G	GLU292B
GLU292C	GLY290B
GLY290C	ILE263G
ILE338C	LYS260G
PRO276F	PRO289C
SER267G	SER277F
THR259G	TYR294C
VAL279E	VAL279F

Acceptor Donor
Metal Contact

Shape -14.49

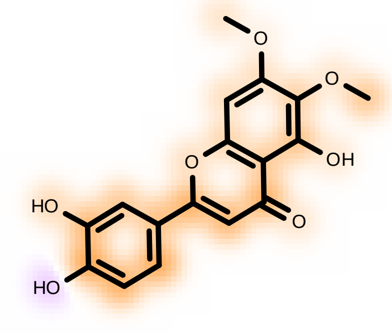

-1.135 0.000 0.600

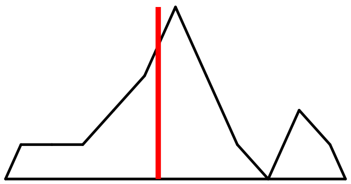

Hydrogen Bond -1.86

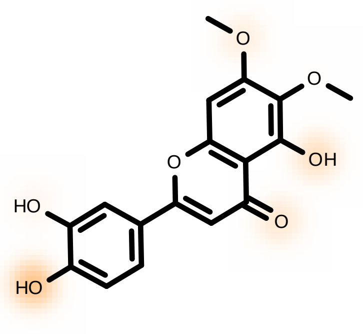

-1.135 0.000 0.600

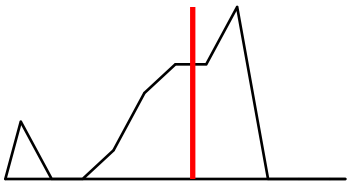

Protein Desolvation 3.65

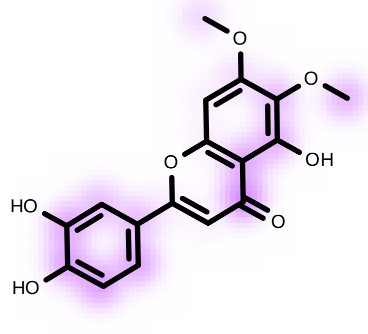

-1.135 0.000 0.600

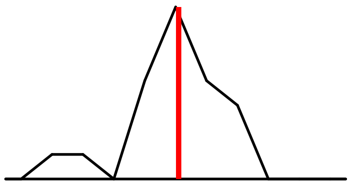

Ligand Desolvation 2.86

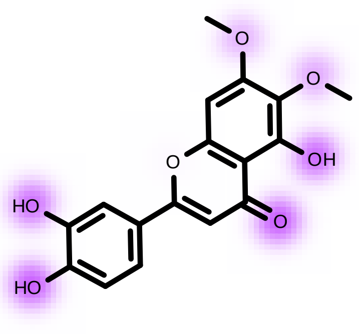

-1.135 0.000 0.600

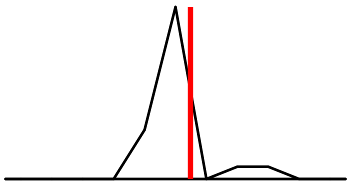

Molecule Name	omega_1_16
Molecular Weight	330.3
XLogP	1.2
PSA	109.4
Heavy Atoms	24
Acceptor Count	7
Donor Count	3
Chelator Count	3
Pose Index	15

Total Score -9.75

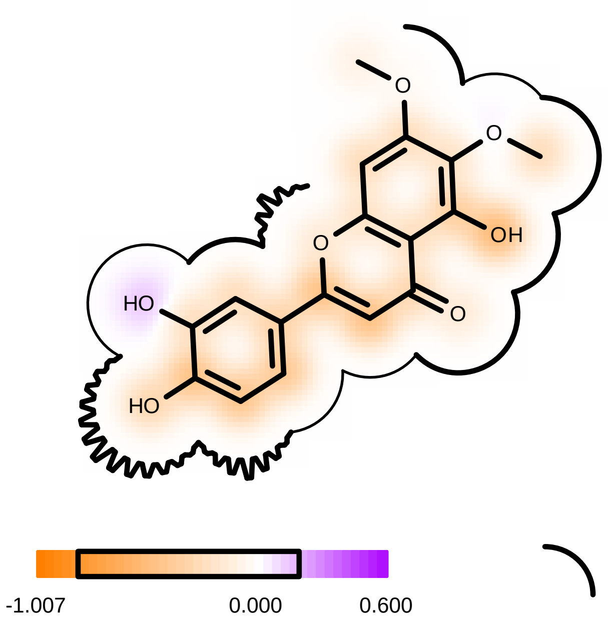

Score compared to other molecules

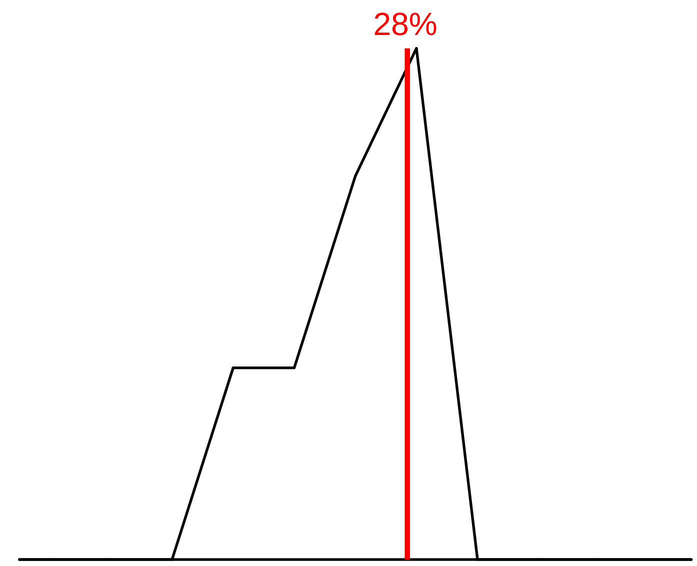

Protein Contact

Protein Cavity

Residue Fingerprint

ALA256G	ALA278E
ALA278F	ALA293B
ALA293C	ALA336B
ARG252G	ARG279C
ARG291B	ASP316F
ASP333B	GLN255G
GLU264G	GLU292B
GLU292C	GLY290B
GLY290C	ILE263G
ILE338C	LYS260G
PRO276F	PRO289C
SER267G	SER277F
THR259G	TYR294C
VAL279E	VAL279F

Acceptor	Donor
Metal	Contact

Shape -14.01

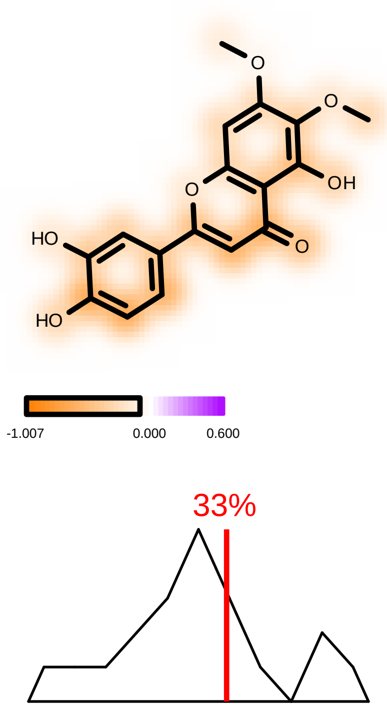

Hydrogen Bond -1.58

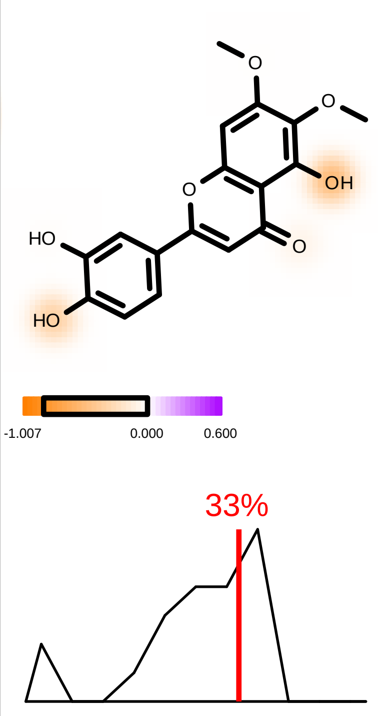

Protein Desolvation 3.28

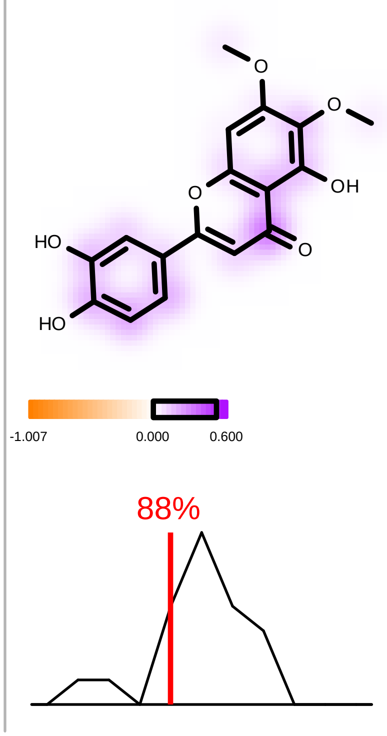

Ligand Desolvation 2.55

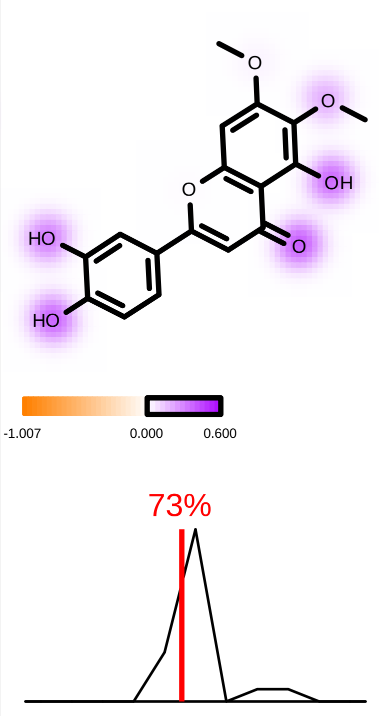

Molecule Name	omega_1_15
Molecular Weight	330.3
XLogP	1.2
PSA	109.4
Heavy Atoms	24
Acceptor Count	7
Donor Count	3
Chelator Count	3
Pose Index	16

Total Score -9.74

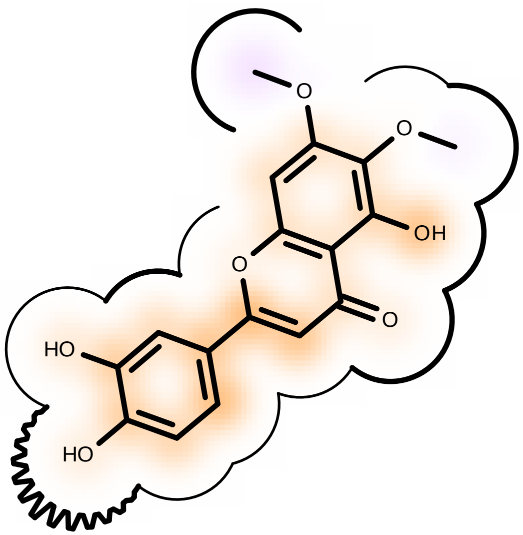

Score compared to other molecules

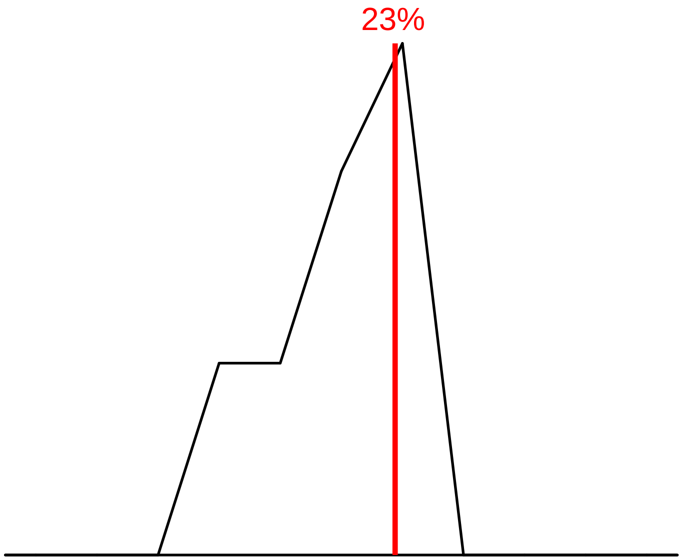

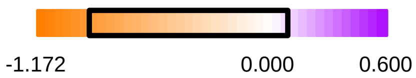

Protein Contact

Protein Cavity

Residue Fingerprint

ALA256G	ALA278E
ALA278F	**ALA293B**
ALA293C	ALA336B
ARG252G	ARG279C
ARG291B	ASP316F
ASP333B	GLN255G
GLU264G	**GLU292B**
GLU292C	GLY290B
GLY290C	ILE263G
ILE338C	LYS260G
PRO276F	PRO289C
SER267G	SER277F
THR259G	TYR294C
VAL279E	VAL279F

Acceptor Donor
Metal Contact

Shape -14.82

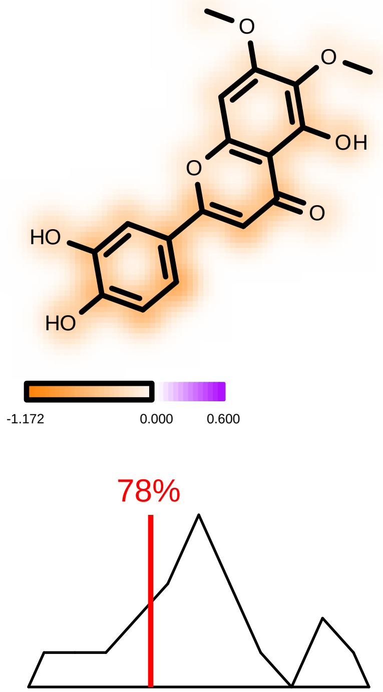

Hydrogen Bond -1.53

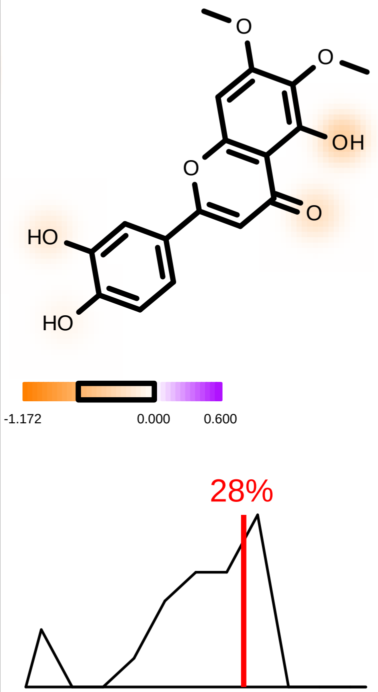

Protein Desolvation 3.98

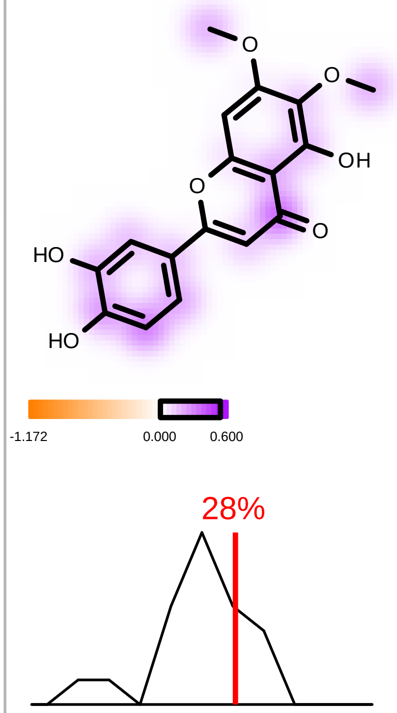

Ligand Desolvation 2.64

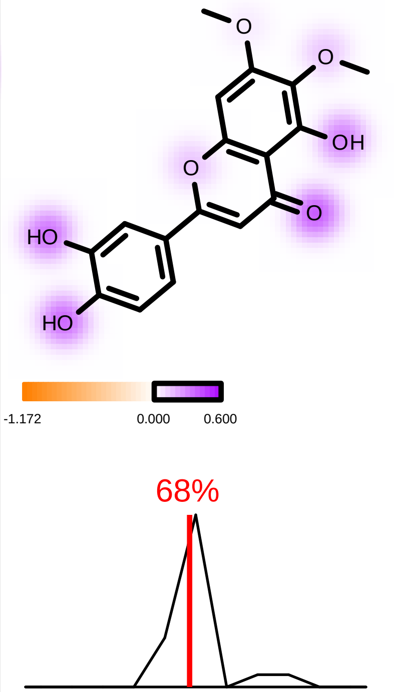

Molecule Name	omega_1_13
Molecular Weight	330.3
XLogP	1.2
PSA	109.4
Heavy Atoms	24
Acceptor Count	7
Donor Count	3
Chelator Count	3
Pose Index	17

Total Score -9.72

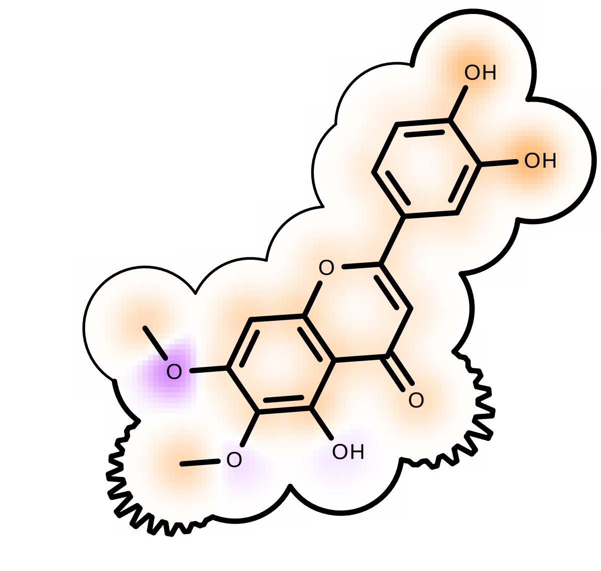

Score compared to other molecules

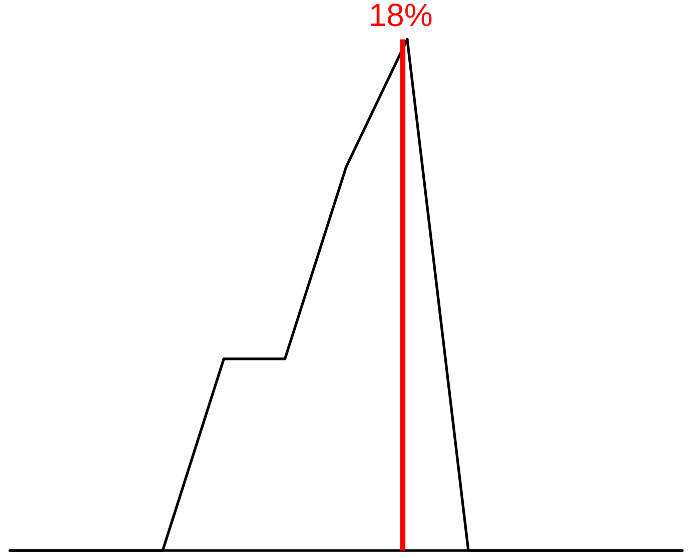

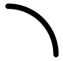
Protein Contact

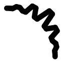
Protein Cavity

Residue Fingerprint

**ALA256G**	ALA278E
ALA278F	ALA293B
ALA293C	ALA336B
ARG252G	ARG279C
ARG291B	ASP316F
**ASP333B**	GLN255G
GLU264G	GLU292B
GLU292C	GLY290B
GLY290C	ILE263G
ILE338C	LYS260G
PRO276F	PRO289C
SER267G	SER277F
**THR259G**	TYR294C
VAL279E	VAL279F

Acceptor	Donor
Metal	Contact

Shape -13.07

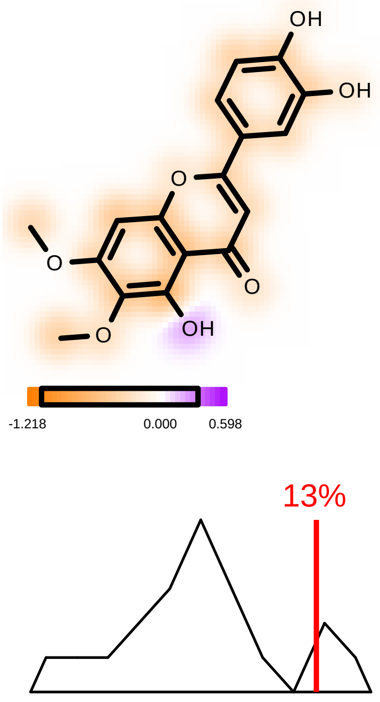

Hydrogen Bond -4.57

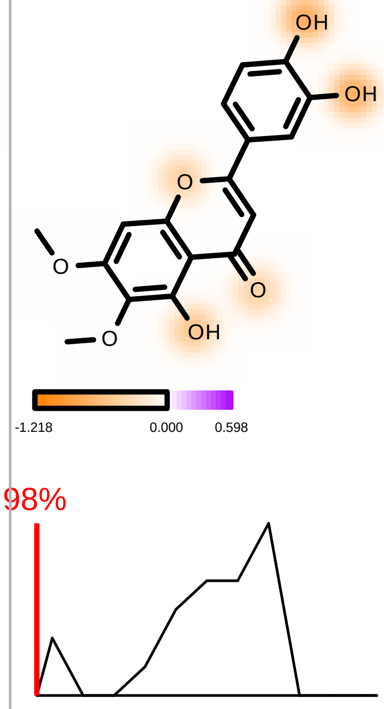

Protein Desolvation 4.24

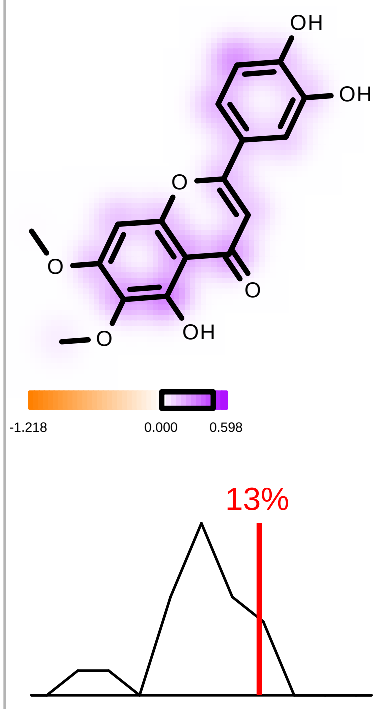

Ligand Desolvation 3.67

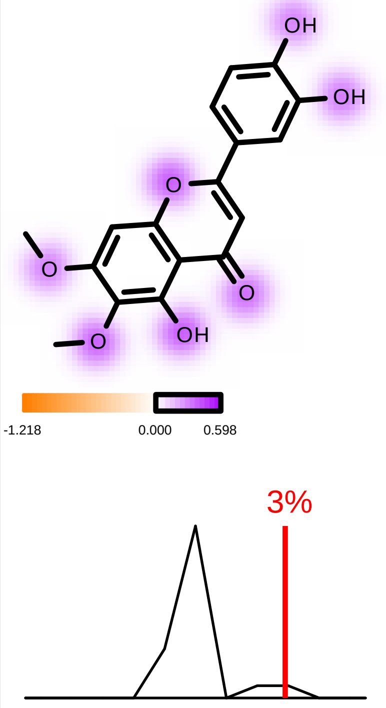

Molecule Name	omega_1_4
Molecular Weight	330.3
XLogP	1.2
PSA	109.4
Heavy Atoms	24
Acceptor Count	7
Donor Count	3
Chelator Count	3
Pose Index	18

Total Score -9.67

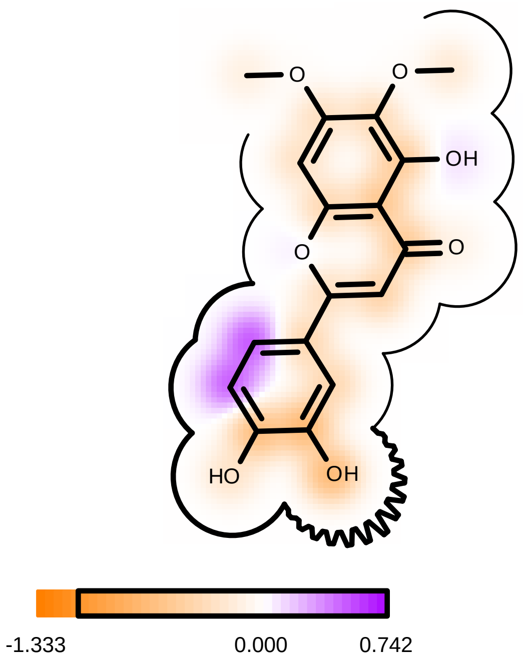

Score compared to other molecules

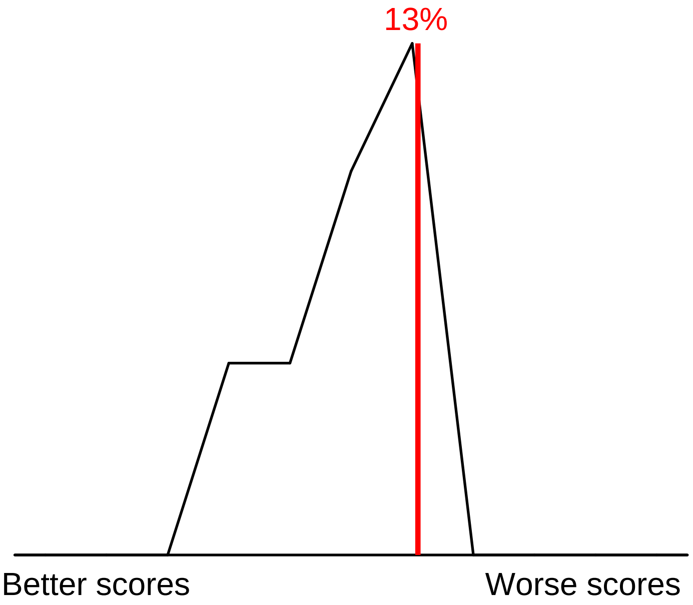

Protein Contact

Protein Cavity

Residue Fingerprint

ALA256G	ALA278E
ALA278F	ALA293B
ALA293C	ALA336B
ARG252G	ARG279C
ARG291B	ASP316F
ASP333B	GLN255G
GLU264G	GLU292B
GLU292C	GLY290B
**GLY290C**	ILE263G
ILE338C	LYS260G
PRO276F	PRO289C
**SER267G**	SER277F
THR259G	TYR294C
VAL279E	VAL279F

Acceptor	Donor
Metal	Contact

Shape -14.46

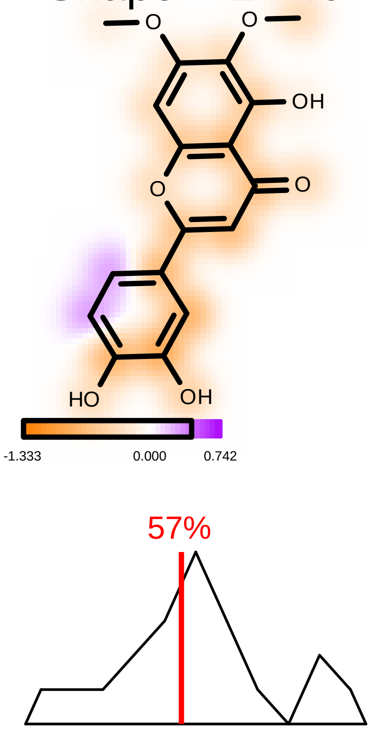

Hydrogen Bond -1.45

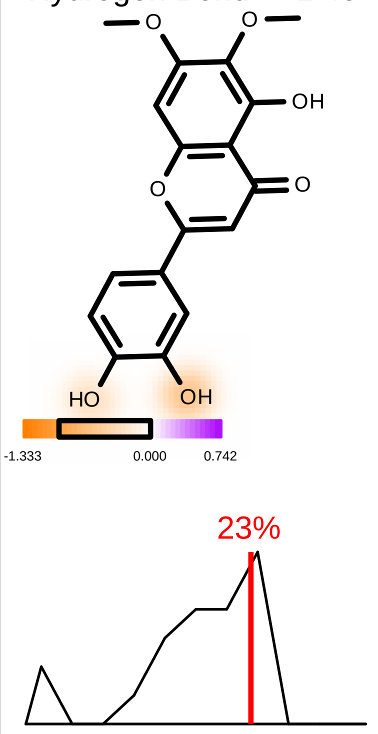

Protein Desolvation 3.75

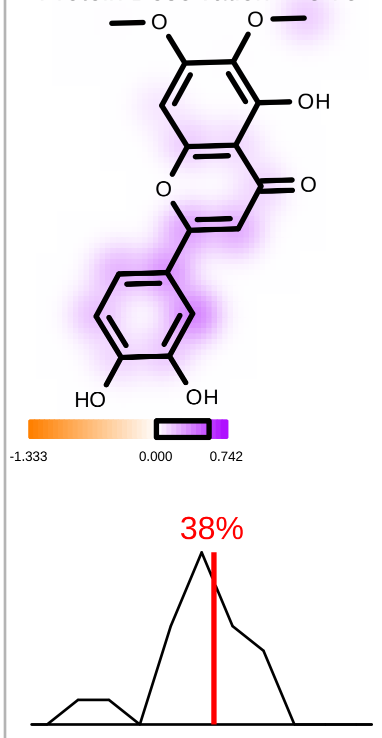

Ligand Desolvation 2.49

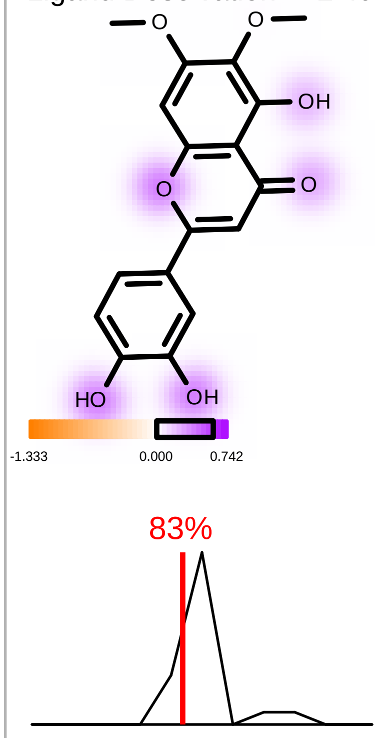

Molecule Name	omega_1_13
Molecular Weight	330.3
XLogP	1.2
PSA	109.4
Heavy Atoms	24
Acceptor Count	7
Donor Count	3
Chelator Count	3
Pose Index	19

Total Score -9.57

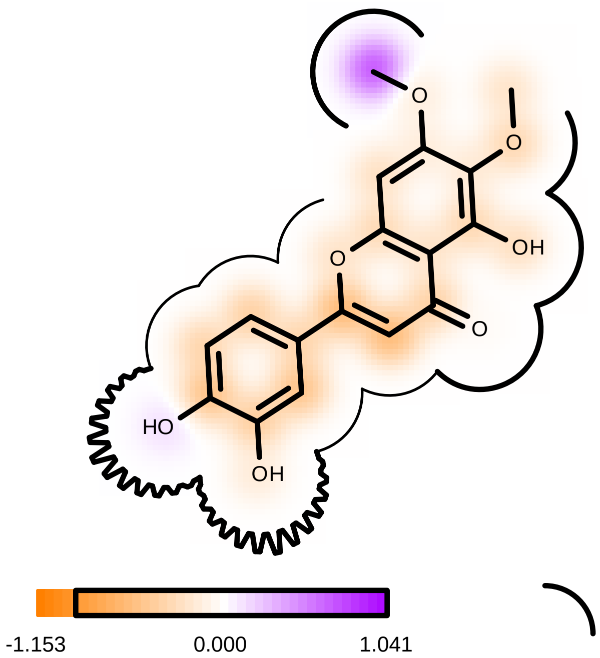

Score compared to other molecules

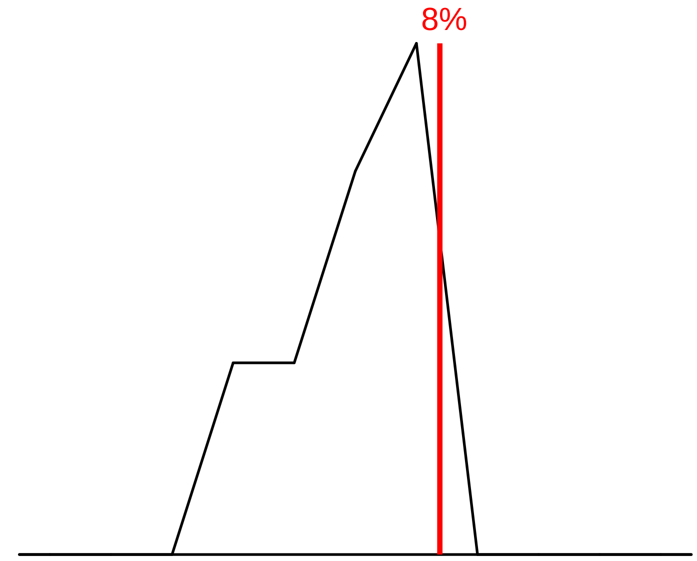

Protein Contact

Protein Cavity

Residue Fingerprint

ALA256G	ALA278E
ALA278F	ALA293B
ALA293C	ALA336B
ARG252G	ARG279C
ARG291B	ASP316F
ASP333B	GLN255G
GLU264G	GLU292B
GLU292C	GLY290B
GLY290C	ILE263G
ILE338C	LYS260G
PRO276F	PRO289C
SER267G	SER277F
THR259G	TYR294C
VAL279E	VAL279F

Acceptor Donor
Metal Contact

Shape -14.15

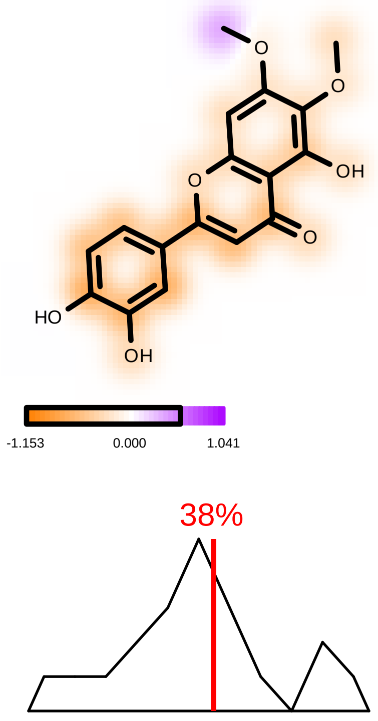

Hydrogen Bond -2.09

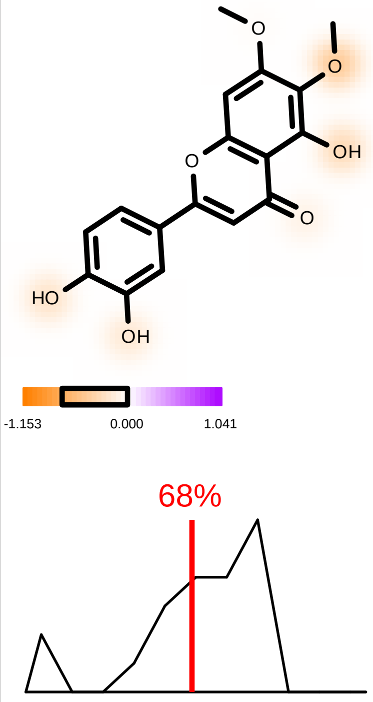

Protein Desolvation 3.85

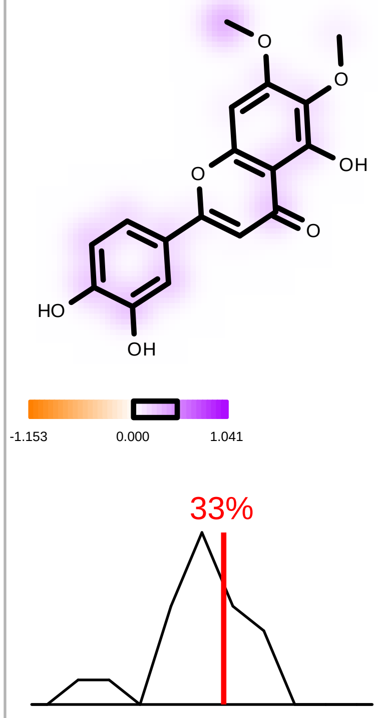

Ligand Desolvation 2.81

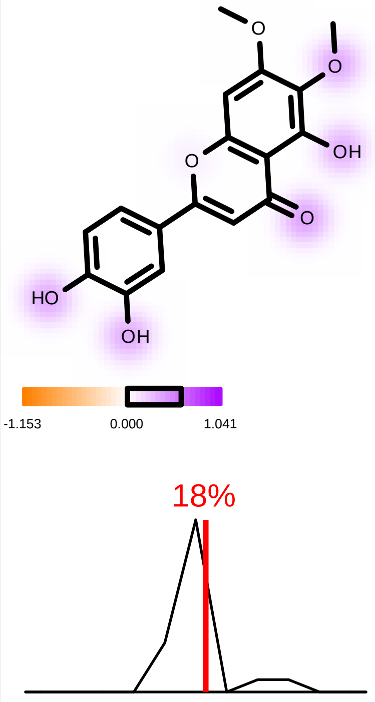

Molecule Name	omega_1_2
Molecular Weight	330.3
XLogP	1.2
PSA	109.4
Heavy Atoms	24
Acceptor Count	7
Donor Count	3
Chelator Count	3
Pose Index	20

Total Score -9.54

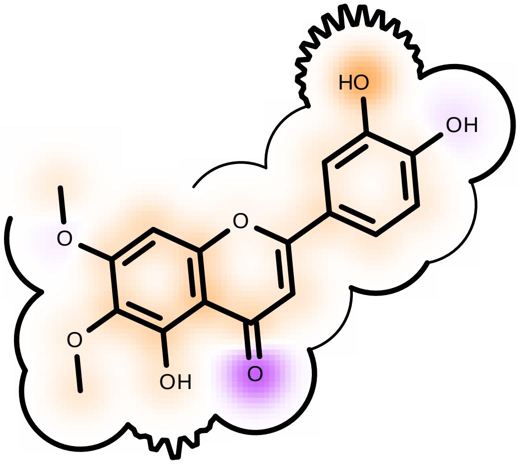

Score compared to other molecules

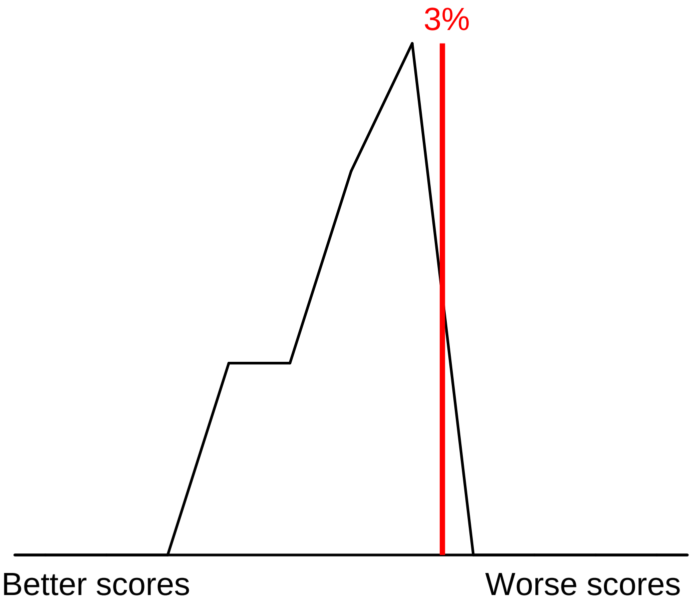

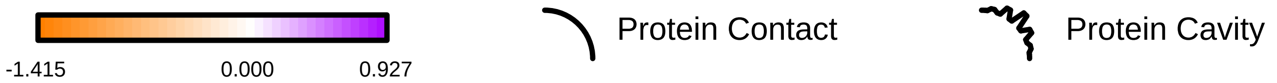

Residue Fingerprint

ALA256G	ALA278E
ALA278F	ALA293B
ALA293C	ALA336B
ARG252G	ARG279C
**ARG291B**	ASP316F
ASP333B	GLN255G
GLU264G	**GLU292B**
GLU292C	GLY290B
GLY290C	ILE263G
ILE338C	LYS260G
PRO276F	PRO289C
SER267G	SER277F
THR259G	TYR294C
VAL279E	**VAL279F**

Acceptor	Donor
Metal	Contact

Shape -13.50

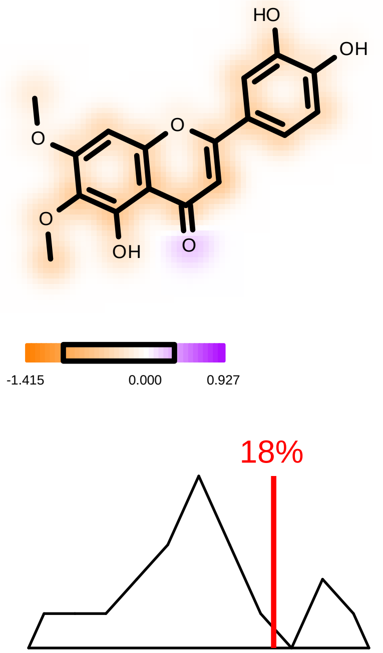

Hydrogen Bond -1.97

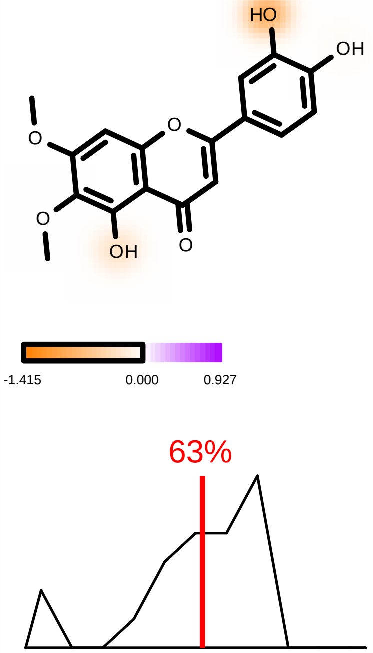

Protein Desolvation 3.67

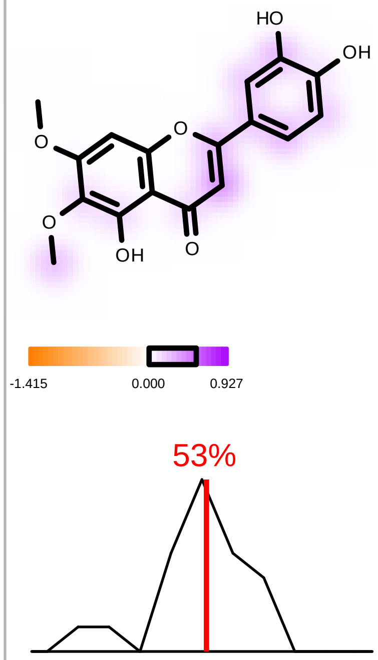

Ligand Desolvation 2.27

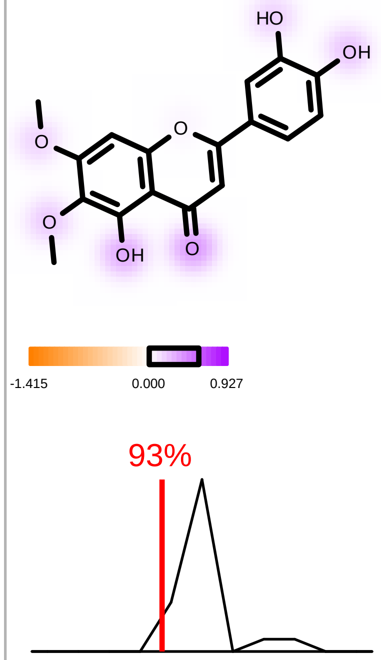

Supplement: Supplementary file 1 [file cells-11-03169-s001.zip › Carlini et al_Supplementary Data S2_FRED_2jiz.pdf]
