# Supplementary material for: The Flavone Cirsiliol from Salvia x jamensis Binds the F1 Moiety of ATP Synthase, Modulating Free Radical Production
Source: Cells. 2022 Oct 9;11(19):3169. doi: 10.3390/cells11193169 (PMC9562182; doi:10.3390/cells11193169)
Supplement: Supplementary file 1 [file cells-11-03169-s001.zip › cells-1852613 - Ssupplementary Figure S1.pdf]

cells

MDPI

Supplementary Materials

The Flavone Cirsiliol from *Salvia x jamensis* Binds the F_1 Moiety of ATP Synthase, Modulating Free Radical Production

Lavinia Carlini [1], Gabriele Tancreda [2], Valeria Iobbi [2], Federico Caicci [3], Silvia Bruno [4], Alfonso Esposito [5], Daniela Calzia [2], Stefano Benini [1], Angela Bisio [2], Lucia Manni [3], Anna Schito [6], Carlo Enrico Traverso [7], Silvia Ravera [4] and Isabella Panfoli [2,*]

[1] Faculty of Science and Technology, Free University of Bolzano, Piazza Università 5, 39100 Bolzano, Italy
[2] Dipartimento di Farmacia (DIFAR), University of Genoa, V.le Benedetto XV 3, 16132 Genova, Italy
[3] Department of Biology, Università di Padova, via U. Bassi 58/B, 35121 Padova, Italy
[4] Department of Experimental Medicine, University of Genoa, Via De Toni 14, 16132 Genoa, Italy
[5] International Centre for Genetic Engineering and Biotechnology (ICGEB), 34149 Trieste, Italy
[6] Dipartimento di Scienze Chirurgiche e Diagnostiche Integrate (DISC), Sezione di Microbiologia, Università di Genova, Largo Rosanna Benzi 8, 16145 Genova, Italy
[7] Clinica Oculistica, (DINOGMI) University of Genoa, V.le Benedetto XV 6, 16132 Genova, Italy
[*] Correspondence: isabella.panfoli@unige.it; Tel.: +39-0103537397

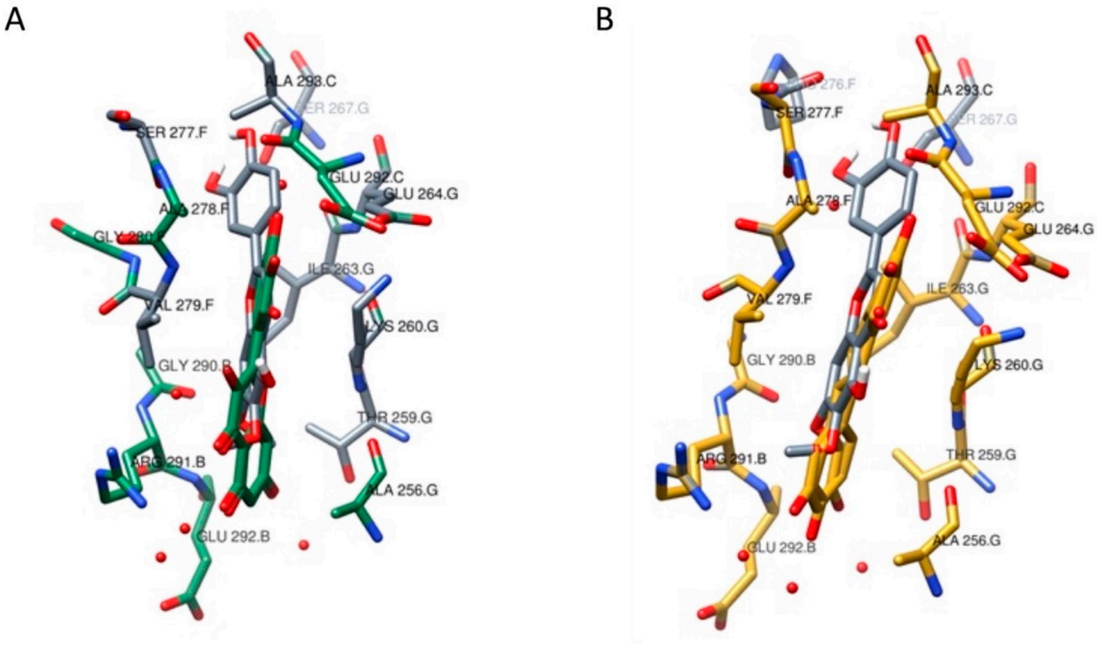

Figure S1. Comparison of the interacting amino acids in the binding of the three different polyphenols with bovine F_1 moiety of ATP synthase. (**A**) Major binding modes of cirsiliol (gray) and cis-quercetin (green). (**B**) Major binding mode of cirsiliol (gray) and resveratrol (yellow). Amino acids Thr-259.G, Lys-260.G, Ile-263.G, Glu-264.G,Ser-277.F, Val-279.F, Ala-293.C and Arg-291.B are always involved in the binding. Amino acids labeled in gray are specific to cirsiliol binding.
